# Epidemiology of soil-transmitted helminths using quantitative PCR and risk factors for hookworm and *Necator americanus* infection in school children in Dak Lak province, Vietnam

**DOI:** 10.1186/s13071-023-05809-x

**Published:** 2023-06-27

**Authors:** Angus Hughes, Dinh Ng-Nguyen, Naomi E. Clarke, Clare E. F. Dyer, Sze Fui Hii, Archie C. A. Clements, Roy M. Anderson, Darren J. Gray, Luc E. Coffeng, John M. Kaldor, Rebecca J. Traub, Susana Vaz Nery

**Affiliations:** 1 School of Population Health, University of New South Wales, Sydney, Australia; 2 Faculty of Animal Sciences and Veterinary Medicine, Tay Nguyen University, Buon Ma Thuot, Dak Lak Vietnam; 3 The Kirby Institute for Infection and Immunity in Society, University of New South Wales, Sydney, Australia; 4 Faculty of Veterinary and Agricultural Sciences, University of Melbourne, Parkville, Australia; 5 Faculty of Health Sciences, Curtin University, Perth, Australia; 6 Telethon Kids Institute, Perth, Australia; 7 Department of Infectious Disease Epidemiology, School of Public Health, Faculty of Medicine, Imperial College London, London, UK; 8 Research School of Population Health, Australian National University, Canberra, Australia; 9 Department of Public Health, Erasmus MC, University Medical Centre Rotterdam, Rotterdam, The Netherlands

**Keywords:** Soil-transmitted helminths, Vietnam, Prevalence survey, Risk factors, Quantitative polymerase chain reaction, School-age children

## Abstract

**Background:**

Soil-transmitted helminth (STH) infection is driven by a complex interaction of demographic, socioeconomic and behavioural factors, including those related to water, sanitation and hygiene (WASH). Epidemiological studies that measure both infection and potential risk factors associated with infection help to understand the drivers of transmission in a population and therefore can provide information to optimise STH control programmes.

**Methods:**

During October and November 2019, we conducted a cross-sectional survey of the prevalence and intensity of STH infection and associated risk factors among 7710 primary-school-age children from 64 primary schools across 13 districts in Dak Lak province, Vietnam. Quantitative PCR (qPCR) was used to detect and quantify STH infections.

**Results:**

The predominant STH species was the hookworm *Necator americanus* (overall cluster-adjusted prevalence of 13.7%), and its prevalence was heterogeneously distributed across surveyed schools (0% to 56.3%). All other STH species had a prevalence of less than 1%. Using mixed-effects logistic regression, we found that the adjusted odds ratio (aOR) was significantly higher for both infection and moderate-to-heavy-intensity infection with *N. americanus* among children from multiple ethnic minority groups, compared to children from the majority group (Kinh). Adjusted odds of infection with *N. americanus* were also higher in children who reported practising open defecation at school (aOR 1.42, 95% CI 1.05, 1.93, *P* = 0.02) and in those who had an unimproved household water supply (aOR 1.28, 95% CI 1.04, 1.57, *P* = 0.02). Conversely, children with a flushing household toilet had a reduced risk of infection (aOR 0.58, 95% CI 0.47, 0.70, *P* < 0.01), as did those whose primary female carer attended secondary (aOR 0.65, 95% CI 0.51, 0.84, *P* < 0.01) or tertiary education (aOR 0.39, 95% CI 0.24, 0.63, *P* < 0.01).

**Conclusions:**

This study is the largest reported prevalence survey of STH infections conducted using qPCR as a diagnostic technique. The findings of higher adjusted odds of infection amongst ethnic minority children highlight that STH control programmes may not be reaching certain population groups and that additional culturally appropriate approaches may be required. Additionally, the associations between specific WASH factors and infection indicate potential programmatic targets to complement preventive chemotherapy programmes.

**Graphical Abstract:**

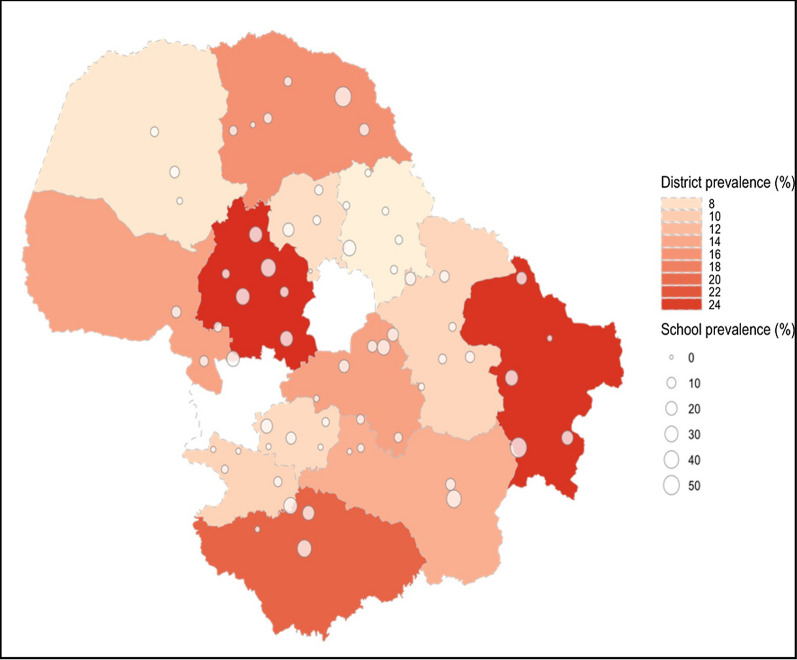

**Supplementary Information:**

The online version contains supplementary material available at 10.1186/s13071-023-05809-x.

## Background

Soil-transmitted helminth (STH) infections are the most prevalent of the neglected tropical diseases (NTDs) globally, infecting approximately 900 million people and contributing more than 1.8 million years lived with disability (YLD) in 2019 [1]. STHs are parasitic intestinal worms, and include hookworms (*Necator americanus*, *Ancylostoma duodenale* and *Ancylostoma ceylanicum)*, *Ascaris lumbricoides*, *Trichuris trichiura* and *Strongyloides stercoralis* [2, 3]. Like other NTDs, they disproportionately affect poorer, rural and indigenous communities in tropical and subtropical regions of the world [2, 4]. High-intensity STH infections are associated with considerable morbidity including malnutrition, anaemia and impaired physical and cognitive development [5–7]. Primary-school-age children along with pre-school-age children and women of reproductive age are groups at greatest risk of STH-associated morbidity [5, 8, 9]. STH control has largely focused on school-based preventive chemotherapy programmes that consist of regular, large-scale administration of anthelminthic medications albendazole and mebendazole, targeting school-age children. The aim is to reduce the prevalence of moderate-to-high-intensity infections, and thus morbidity, in schoolchildren to below 2% by 2030 [8]. While albendazole and mebendazole are effective against hookworm, *A. lumbricoides* and *T. trichiura*, they are not effective against *S. stercoralis*, and therefore the World Health Organization (WHO) recommends the addition of ivermectin to the current programmes in areas where *S. stercoralis* prevalence is higher than 10% [8].

Transmission of STH infection occurs through the uptake, via ingestion or skin penetration depending on the species, of the infective forms of the parasites (eggs or larvae), after they have been introduced to the environment through contamination with human faeces [10]. Improved water, sanitation and hygiene (WASH) services and systems, such as piped water, flushing toilets and handwashing facilities, are therefore key in disrupting the transmission of STHs [10–13], but implementation and uptake of such facilities depend on a complex interaction of socioeconomic, cultural and behavioural factors [11–17]. Understanding current local factors, particularly WASH, is essential to guide STH control strategies that are recommended by WHO to complement preventive chemotherapy programmes [8, 18].

STH infection prevalence and intensity surveys have generally relied upon Kato-Katz microscopy, which remains the main diagnostic technique recommended by WHO [8]. Kato-Katz involves the direct identification and counting of the helminth eggs in fresh stool, and its low cost and simplicity make it attractive to assess STH prevalence in endemic settings [19, 20]. However, the technique is limited by poor sensitivity, particularly in low transmission settings, is highly dependent on operator skill, requires fresh stool to be processed within 60 min to prevent degradation of hookworm eggs, is unable to distinguish between hookworm species and is unable to detect *S. stercoralis* [21–24]. Quantitative polymerase chain reaction (qPCR) is a powerful alternative option [21, 22, 24], which overcomes all of these limitations [21, 22, 24–27]. Furthermore, recent work has enabled the conversion of qPCR-derived cycle-threshold (Ct) values into conventional eggs per gram (EPG) values, allowing for the estimation of STH infection intensity as a proxy indicator of morbidity and demonstrated that this can be applied to epidemiological surveys [27–29].

In Vietnam, including Dak Lak province in the central highlands, STHs have been a long-standing public health problem [2, 30]. Therefore, Vietnam has been scaling up preventive chemotherapy programmes targeting school-age children since 2002 [31]. Dak Lak province has had large-scale preventive chemotherapy programmes for school-age children using albendazole or mebendazole in place since 2007 [32]. Preventive chemotherapy has been conducted twice annually in schools prior to 2019 and annually from 2019 onwards [32]. In Dak Lak province, the setting for this study, there is limited previous literature on STH prevalence and burden. One previous assessment of STH prevalence in school-age children in 2015 sampled 1206 ethnic Êđê children from four schools in a single district and found an overall STH prevalence of 25% with 22.8% due to hookworm (mainly *N. americanus*), 2.0% due to *A. lumbricoides*, 0.33% due to *T. trichiura* and did not assess for *S. stercoralis* [33]. Additionally, the country has made substantial gains over the last 20 years to improve WASH access and services [34]. However, gains have been slower in rural areas such as Dak Lak for people of lower socioeconomic status and for people from ethnic minorities [34, 35].

In this cross-sectional survey, which we believe to be the largest reported survey assessing STH epidemiology using qPCR as the diagnostic method, we aim to quantify STH prevalence and infection intensity and associated risk factors for hookworm and *N. americanus* infection in school-age children.

## Methods

### Study setting, design, and participants

Dak Lak province is located in the central highlands of Vietnam, approximately 1410 km south of the capital, Hanoi. With an area of 13,125 km^2^ and population of approximately 1.9 million [36] representing 44 ethnic groups, Dak Lak is divided into 13 non-urban districts, one city and one town [36]. Districts are made up of 182 communes, wards and towns, with each subdivided into hamlets that will maintain between 100 and 1000 households.

We conducted a cross-sectional survey of primary-school-age students in grades 1–4 from 64 primary schools located in rural, remote or very remote regions as defined by the Department of Education in the 13 non-urban districts of Dak Lak across October and November 2019. One hundred and twenty-nine eligible schools were initially identified, and 64 schools were randomly selected from all eligible schools across the province stratified by district to ensure good geographical coverage across Dak Lak province. This survey was conducted as the baseline assessment for the CoDe-STH (Community Deworming against Soil-Transmitted Helminths) trial, described in detail elsewhere [32]. The CoDe-STH trial is a two-arm 1:1 cluster-randomised control trial comparing community-wide mass treatment with primary school-based targeted treatment [32], in regard to impact on STH prevalence. The sampling strategy and size for this analysis were derived from the CoDe-STH trial [32]. Post hoc power analysis indicated that a sample size of 7680 across 64 clusters with a cluster size of 120 students was sufficient to detect a hookworm prevalence of 20% with a power of 90%, alpha of 0.05 and intra-cluster correlation coefficient of 0.12 [32]. Dak Lak was chosen as the setting for this study due to STH being a significant public health problem in the province and an established collaboration with Tay Nguyen University (TNU), located in Dak Lak province.

Participant recruitment, consent and data collection were conducted by a research field team consisting of a local project manager and six field supervisors from TNU, four local staff recruited from the Dak Lak Department of Health and Department of Education for the district, and one local health worker per hamlet health centre (268 different hamlets) [32]. The procedures for school, parent/caregiver and child engagement and recruitment in the study are discussed in detail in the trial protocol [32]. In brief, schools selected to participate in the trial were notified and invited through the Department of Education and, once consented to participate, agreed dates were set between the school and research field team to visit and explain the trial, obtain consent, and collect data. Parents and caregivers for children were notified by the school via a letter and invited to attend the school on the agreed date to meet with the research team, discuss the trial and participation, obtain consent from parents/caregivers and assent from participating children. Once consent and assent had been obtained, interviews with parents/caregivers and children to collect data and stool sample collection occurred over 2–3 days at the school, details on interviews and stool sample collection are detailed in the following sections.

### Demographic, socioeconomic and WASH data collection

Face-to-face interviews with participating students and their parents/carers were conducted in Vietnamese to collect demographic, socioeconomic and WASH information. Information collected from students included demographic data, receiving recent anthelminthic medication and personal hygiene behaviour (defecation practices, hand hygiene and shoe-wearing). Information collected from parents/carers included water supply at home, household income, female carer education and main carer occupation. Research team members also inspected participating schools with a senior teacher or principal to collect data on school water supply and the number, type, condition, and usability of school toilets. Questionnaires were based on previous surveys conducted in other settings and the WHO/United Nations International Children’s Emergency Fund (UNICEF) Joint Monitoring Programme (JMP) indicators and are provided with the Additional file for the trial protocol [32, 37, 38].

All demographic and WASH data were collected offline using electronic tablets with a REDCap data capture tool and then sent to a secure REDCap database hosted at the University of New South Wales, Sydney [32, 39].

### Specimen collection and analysis

Students were instructed on how to collect an early morning stool sample at home using a supplied kit and asked to bring the sample to school for collection. The stool samples were processed immediately on receipt. An aliquot of stool was prepared by adding 3 g of the stool sample to 3 mL of 5% (*w*/*v*) potassium dichromate and they were then kept chilled on ice and refrigerated upon arrival at the TNU laboratory [32].

Preserved stool samples were sent to the University of Melbourne, Australia for analysis using qPCR [21, 25, 27, 28]. DNA extraction was performed using a Maxwell RSC PureFood GMO and Authentication Kit and a Maxwell RSC 48 Instrument (Promega Corporation, USA). Two probe-based real-time multiplex qPCR assays were used to detect infection with *N. americanus*, *A. ceylanicum*, *A. duodenale*, *A. lumbricoides*, *T. trichiura* and *S. stercoralis*, and quantify infection intensity for *N. americanus*, *A. lumbricoides* and* T. trichiura* [21, 25–28]. Each multiplex PCR was run twice on each sample to obtain two qPCR-derived Ct values. All samples with Ct values in duplicate were deemed positive. The average Ct value for each sample was converted into EPG of stool using linear regression equations derived from stool seeding experiments [27]. In short, this involved serial dilution of known quantities of fully embryonated eggs which were spiked into faeces free from STH, allowing for the determination of standard curves of Ct value versus EPG values [27]. Time in transit during transport of samples from Vietnam to Australia for analysis led to the embryonation of eggs in samples, and therefore the following previously derived linear regression equations for embryonated eggs were used to convert Ct values into EPG values: *N. americanus* EPG = 10^([Ct−32.657]/−3.878)^; *A. lumbricoides* EPG = 10^([Ct−30.048]/−3.2804)^; *T. trichiura* EPG = 10^([Ct−31.888)/−4.048)^ [27, 28]. EPG values were then classified into one of the three infection intensity classes, light, moderate or heavy, according to WHO guidelines [40].

### Georeferencing of schools

Geographical data regarding district boundaries within Dak Lak province were sourced as a shapefile from the open-source Humanitarian Data Exchange platform [41]. Coordinates of each school were obtained using hand-held global positioning system receivers. The coordinates were recorded and used for mapping the schools. Maps were created using the open-source RStudio integrated development environment (version 1.4.1717) statistical software [42, 43] and the R package ggplot2 [44].

### Statistical analysis

All data management and statistical analysis was undertaken using Stata/IC (version 16.1) [45]. The prevalence of infection for all species (*N. americanus*, *A. duodenale* and *A. ceylanicum*, *A. lumbricoides*, *T. trichiura* and *S. stercoralis*), and the prevalence of light-, moderate- and heavy-intensity infection for *N. americanus*, *A. lumbricoides* and *T. trichiura* and 95% confidence intervals (95% CI) were estimated using mixed-effects logistic regression models with school as a random effect term to account for the clustered sampling by school. Prevalence estimates were also stratified by sex, ethnicity, and district. Mean and negative binomial 95% CI were reported for the EPG values for *N. americanus*, *A. lumbricoides* and *T. trichiura.*

Mixed-effects logistic regression with school as a random effect to account for clustering was used to examine demographic, WASH and socioeconomic risk factors associated with STH infections and factors associated with moderate-to-heavy-intensity infections [14, 46].

WASH questionnaire answers were categorised to align with WHO/UNICEF JMP definitions for improved and unimproved water, hygiene and sanitation services in schools and households [38]. We compared the odds of exposure variables in moderate-to-heavy-intensity to light-intensity infections to examine associations with moderate-to-heavy infection amongst infected individuals.

The model-building procedure was similar to previously published STH risk factor analyses [28, 46, 47]. In summary, variables were grouped into the following domains: student demographics, STH co-infection, student anthelminthic status, hygiene and defecation behaviour, household WASH factors and socioeconomic factors, school location and WASH factors. Univariable regression analyses were carried out with each variable, with variables retained if *P* < 0.20. Multivariate regressions were then run for each domain with the inclusion of age and sex covariates along with retained variables. Variables were retained from each domain-level model if *P* < 0.10. Retained variables were tested for multicollinearity utilising variance inflation factors (VIF) with a cut-off of > 5 used to indicate collinear variables. Multiple multivariate models of the retained variables, and including age and sex, were then run, with each collinear variable and the Akaike information criteria (AIC) used to compare models and select variables to retain based on a lower AIC, which indicates model performance, trading off goodness of fit versus model simplicity. To achieve the most parsimonious adjusted model, backward stepwise elimination of variables was then performed until all remaining covariates, except for age and sex, had a *P*-value of < 0.05. Final multivariate models are reported with adjusted odds ratios (aOR) and their 95% confidence intervals. Complete case analysis was used to handle missing data for risk factor analysis, with participants only included at each step if they had data for all predictor variables.

A sensitivity analysis was performed using the same procedure as described above but adjusting for a third level of clustering, at the hamlet level, below the level of school in the hierarchy of data structure.

## Results

### Participant characteristics

From the 64 schools, 14,116 participants (students) were enrolled in the survey, of whom 8730 (61.8%) provided a stool sample (range 70–219 samples per school), 9698 (68.7%) completed a student questionnaire and 10,752 (76.1%) had a completed caregiver questionnaire (Additional file 1: Fig S1). Due to financial restrictions, qPCR was performed on an average of 120 samples per school (range 70–135) resulting in 7710 out of the 8730 stool samples provided being analysed. Of these 7030 had both an individual student and caregiver questionnaire and therefore were included in the data set for the risk factor analysis. Additional file 1: Fig S1 describes participant selection.

The mean age of those included in the risk factor analysis (Table 1) was 7.9 years (SD 1.23 years), and 51.4% were female. The majority identified as ethnic Kinh (39.7%), followed by Êđê (29.2%) (Table 1). Just under half (45.6%) reported receiving anthelminthic medications at school in the last year, and 46.3% and 14.2% reported practising open defecation at home and school respectively (Table 1). The majority of students reported their house having access to a toilet/latrine and handwashing station at home (75.3% and 91.1% respectively), and having an improved household water source (82.3%). Household socioeconomic characteristics were relatively homogeneous among students, with the occupation of the main household income earner being a farmer for over 90% of students and most reporting an annual household income of less than 50 million Vietnamese dong (VND; approximately $2150 US dollars [USD]) (Table 1). Most schools were rural (56.3%), with fewer classed as remote (32.8%) and very remote (10.9%) (Table 1). All schools reported having a toilet, with 96.9% reporting having toilets available for student use at the time of the survey and 95.2% reporting having improved sanitation available (Table 1). Over 88% reported having a handwashing station available, but only around a third had soap available at the station (32.2%) (Table 1). Additional file 1: Table S1 reports full descriptive characteristics for each variable and variable categories and Additional file 1: Table S2 describes the number of children by ethnicity for all participants. Additional file 1: Table S3 compares the demographic, WASH, and socioeconomic characteristics of those who did and did not provide a stool sample and compares those that did and did not have qPCR performed on the stool sample. Generally, most characteristics appeared similar between the groups.

**Table 1:** Selected participant characteristics for students with complete data across all questionnaires and included for risk factor analysis, total *N* = 7030^a^

Variable		%	*n*
Demographics			
Age		7.9 years	±1.23
Female		51.37	(3611)
Ethnicity	Kinh	39.70	(2791)
	Êđê	29.20	(2053)
	Mnông	5.72	(402)
	Nùng	5.85	(411)
	Tày	5.87	(413)
School grade	Grade 1	26.24	(1845)
	Grade 2	27.98	(1967)
	Grade 3	23.41	(1646)
	Grade 4	22.35	(1571)
Student anthelminthic status, hygiene, and defecation behaviour			
Received anthelminthic in last year		45.61	(3202)
Always uses household latrine^b^		80.27	(4243)
Usual place of defecation at home	Toilet/latrine	75.91	(5326)
	On the ground/bushes outside	21.81	(1530)
Reports ever defecating outside at home^c^		46.31	(3235)
Usual place of defecation at school	Toilet/latrine	91.22	(6390)
	On the ground/bushes outside	5.47	(383)
Reports ever defecating outside at school^c^		14.22	(993)
Always wears shoes outside		72.25	(5071)
Always wears shoes defecating		66.95	(4702)
Always washes hands after defecating		59.72	(4192)
Always washes hands before eating		51.55	(3614)
Household WASH			
Household has an improved water source^d^		82.33	(5786)
Household has latrine		75.25	(5286)
Handwashing station at home		91.14	(6377)
Household socioeconomic			
Household income^e^	Less than 20,000,000 VND	45.01	(3161)
	VND 20,000,000–50,000,000	26.04	(1829)
	VND > 50,000,000	7.86	(507)
Main household income earner’s occupation is a farmer		91.20	(6407)
Primary female carer's highest education level completed	Never attended school	8.00	(562)
	Completed or attended primary school	29.49	(2072)
	Completed or attended secondary school	49.60	(3484)
	Completed or attended tertiary education	9.17	(645)
School location and WASH^f^			
Location	Rural	56.25	(36)
	Remote	32.81	(21)
	Very remote	10.94	(7)
School has an improved water source^d^		42.19	(27)
Latrine/toilet available for students to use^g^		96.88	(62)
School has improved sanitation		95.24	(7,351)
Handwashing station with soap available		32.23	(2,410)
Between 1 and 5 toilets available for students to use		62.50	(40)
Between 1 and 5 toilets available for girls		91.94	(57)
Between 1 and 5 toilets available for boys		96.83	(61)

This table reports selected characteristics. The 7030 students were from 6726 unique households, where 6448 students were a single child from their household, 508 students were pairs from their household (254 households), 66 were trios from their household (22 households) and eight were a quartet from their household (two households)

^a^See Additional file 1: Table S1 for complete descriptive characteristics for all variables and categories and Additional file 1: Table S2 for all ethnicities

^b^Responses only recorded for those who responded yes to ‘Household has latrine’, percentage of *N* = 5286

^c^Refers to ever reporting defecating outside at home or school, i.e. practises open defecation

^d^Response for variables collapsed to align with WHO and United Nations International Children’s Emergency Fund (UNICEF) Joint Monitoring Programme (JMP) for Water Supply and Sanitation definitions. Improved water = piped water, protected well, rainwater or public tap. Unimproved water = unprotected well or surface water. Improved sanitation = flush toilet or pit latrine with a slab. Unimproved sanitation = pit latrine without a slab

^e^10 million VND = approximately $430 USD as of 25 May 2022

^f^Percentages reported out of number of schools, *N* = 64

^g^No schools reported having no latrine/toilet

### Prevalence and STH infection intensity

**Fig. 1:**
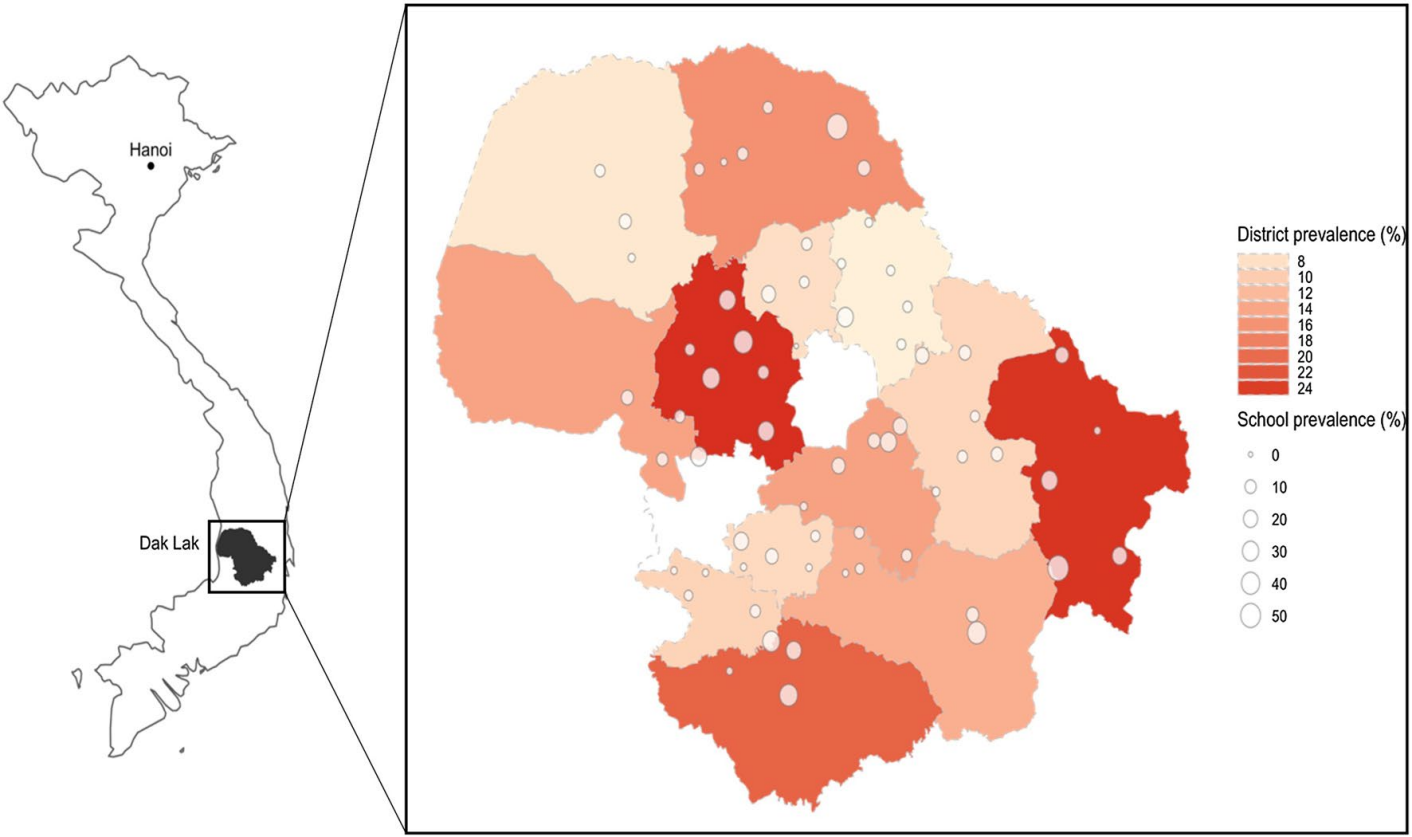
*Necator americanus* prevalence map of 64 schools and 13 districts surveyed in Dak Lak province. Observed prevalence reported for each school. District prevalence calculated as the average observed prevalence of all schools in each district. Unshaded map areas correspond to Buôn Ma Thuột and Buôn Hồ, which were not included in this survey. The base map of Vietnam was sourced from http://www.naturalearthdata.com/about/terms-of-use/ and the shapefile for lower level administrative boundaries for Dak Lak and its districts were sourced from the Humanitarian Data Exchange https://data.humdata.org/dataset/cod-ab-vnm?

The cluster-adjusted prevalence and the distribution of intensity of infection for evaluated STH species are reported in Table 2. The majority of infections were hookworm (cluster-adjusted prevalence 14.2%, 95% CI 10.6–17.7), with *N. americanus* being the most common species, with a cluster-adjusted prevalence of 13.7% (95% CI 10.2–17.22). The geographical distribution of *N. americanus* infections by school and district is shown in Fig. 1. *Necator americanus* prevalence was very heterogeneous across the 64 schools in 13 districts, ranging from 0 to 56.3%, and with 16 (25%) of the total 64 schools having a prevalence greater than 20%. All districts had at least one school with an observed prevalence greater than 10%. All other STH species detected (*A. duodenale*, *A. ceylanicum*, *A. lumbricoides*, *T. trichiura* and *S. stercoralis*) had a cluster-adjusted prevalence of less than 1% (Table 2). Co-infection with two different STH species was rare at 0.9% and there were no students with three or more species. The proportion of *N. americanus* infections that were light, moderate and heavy were 76.3%, 11.3% and 12.4% respectively. The cluster-adjusted prevalence of moderate and heavy-intensity infections of *N. americanus* was 3.5% (95% CI 2.09–4.99). All infections of *T. trichiura* and *A. lumbricoides* detected were light intensity (Table 2). Additionally, Additional file 1: Tables S4, S5 summarise the cluster-adjusted prevalence by district and ethnicity.

**Table 2:** Cluster-adjusted prevalence and intensity of soil-transmitted helminth infection by species

	Study sample % (*n*)			Hookworm							
			Any STH^a^	All hookworm	*N. americanus*	*A. ceylanicum*	*A. duodenale*	*A. lumbricoides*	*T. trichiura*	*S. stercoralis*	All evaluated STH^b^
Prevalence											
Female	51.15 (3.944)	%	12.67	11.87	11.43	0.56	0.10	0.22	0.77	0.51	12.87
		(95% CI)	(9.41, 15.93)	(8.64, 15.09)	(8.24, 14.61)	(0.31, 0.80)	(0.00, 0.22)	(0.00, 0.45)	(0.32, 1.22)	(0.27, 0.75)	(9.57, 16.18)
Male	48.85 (3.766)	%	17.34	16.65	16.24	0.80	0.028	0.34	0.75	0.58	17.62
		(95% CI)	(13.37, 21.31)	(12.65, 20.64)	(12.23, 20.22)	(0.49, 1.10)	(0.00, 0.085)	(0.019, 0.65)	(0.31, 1.20)	(0.32, 0.85)	(13.60, 21.64)
Total	100 (7.710)	%	14.91	14.15	13.73	0.67	0.066	0.28	0.76	0.55	15.15
		(95% CI)	(11.39, 18.42)	(10.64, 17.66)	(10.24, 17.22)	(0.47, 0.88)	(0.00, 0.14)	(0.038, 0.52)	(0.35, 1.16)	(0.36, 0.73)	(11.59, 18.71)
		Range^c^	0–57.48	0–56.30	0–56.30	0–4.24	0–1.67	0–2.54	0–7.56	0–3.33	0–57.78
Infection intensity class											
Light^d^		%	–	–	10.49	–	–	0.28	0.76	–	–
		(95% CI)	–	–	(7.79, 13.20)	–	–	(0.038, 0.52)	(0.35, 1.16)	–	–
Moderate^e^		%	–	–	1.69	–	–	–	–	–	–
		(95% CI)	–	–	(0.88, 2.50)	–	–	–	–	–	–
Heavy^f^		%	–	–	1.81	–	–	–	–	–	–
		(95% CI)	–	–	(1.06, 2.56)	–	–	–	–	–	–
Eggs per gram											
		Mean	–	–	1678.79	–	–	334.23	21.72	–	–
		(95% CI)^g^	–	–	(1489.1, 1892.7)	–	–	(125.8, 888.1)	(14.3, 32.9)	–	–
		Range	–	–	0.031–35737.2	–	–	0.37–4357.03	0.031–232.99	–	–

*95% CI* 95% confidence interval. Prevalence calculated amongst participants who provided a stool sample at baseline in 2019 that had qPCR performed on that sample

^a^‘Any STH’ defined as the detection of at least one of the following STH species: the hookworms (*N. americanus*, *A. ceylanicum*, *A. duodenale*), *A. lumbricoides* or *T. trichiura* in stool by qPCR. This definition of ‘any STH’ is to align with previously published estimates of STH prevalence that relied only upon microscopic techniques and thus could not effectively evaluate for the presence of *S. stercoralis*

^b^‘All evaluated STH’ defined as the detection of at least one of all the evaluated STH species the hookworms (*N. americanus*, *A. ceylanicum*, *A. duodenale*), *A. lumbricoides*, *T. trichiura* or *S. stercoralis* in stool by qPCR

^c^Range reporting the range of observed prevalence for each STH species across schools. 95% CI was calculated using robust standard errors to account for clustering. Infection intensity classes are according to WHO guidelines for classifying infection intensity based on EPG values

^d^Light-intensity infection: *N. americanus* (1–1999 EPG), *A. lumbricoides* (1–4999 EPG), *T. trichiura* (1–999 EPG)

^e^moderate: *N. americanus* (2000–3999 EPG)

^f^heavy: *N. americanus* (≥ 4000 EPG)

^g^Eggs per gram 95% CI reported as negative binomial confidence bounds

### Risk factors for hookworm and *N. americanus* infection

Because the prevalence of *A. duodenale*, *A. ceylanicum*, *A. lumbricoides*, *T. trichiura* and *S. stercoralis* was less than 1%, only *N. americanus* and hookworm (combination of *N. americanus*, *A. duodenale* and *A. ceylanicum*) were considered in the risk factor analysis. Table 3 summarises the results of the multivariate analysis for both all hookworm and *N. americanus* infection (See Additional file 1: Tables S6, S7. For both outcomes, females had reduced odds of infection compared to males (hookworm: aOR 0.56, 95% CI 0.48–0.66, *P* < 0.01; *N. americanus*: aOR 0.55, 95% CI 0.47–0.65, *PP* < 0.01) and every year increase in age, increased the odds of infection (hookworm: aOR 1.26, 95% CI 1.19–1.34, *P* < 0.01; *N. americanus*: aOR 1.28, 95% CI 1.21–1.36, *P* < 0.01) (Table 3). All minority ethnic groups were associated with increased risk of infection for all hookworm and *N. americanus* compared to children of Kinh ethnicity (Table 3).

**Table 3:** Factors associated with all hookworm and *Necator americanus* infection

			All hookworm (*N* = 6964^†^)			*N. americanus* (*N* = 6964^†^)		
Variable		% (*n*)*	aOR	95% CI	*P*-value	aOR	95% CI	*P*-value
Age		7.9 ± 1.23	1.27	1.19–1.35	< 0.01	1.28	1.21–1.36	< 0.01
Female		51.3 (3.611)	0.56	0.48–0.66	< 0.01	0.55	0.47–0.65	< 0.01
Ethnicity^a^	Kinh	39.7 (2.791)	Reference			Reference		
	Dao	2.0 (143)	3.05	1.54–6.04	< 0.01	3.35	1.65–6.82	< 0.01
	Gia-rai	3.2 (225)	8.34	4.40–15.78	< 0.01	9.77	5.09–18.74	< 0.01
	Hmông	3.3 (235)	22.00	12.66–8.21	< 0.01	25.67	14.55–45.26	< 0.01
	Mnông	5.7 (402)	7.45	4.21–13.19	< 0.01	7.83	4.35–14.09	< 0.01
	Nùng	5.9 (411)	3.39	2.09–5.50	< 0.01	3.88	2.35–6.40	< 0.01
	Tày	5.9 (413)	1.87	1.05–3.34	0.034	2.10	1.16–3.83	0.015
	Xơ-đăng	1.2 (84)	44.85	20.49–98.19	< 0.01	59.16	26.42–132.48	< 0.01
	Êđê	29.2 (2.053)	8.88	6.22–12.70	< 0.01	9.77	6.72–14.20	< 0.01
	Other	3.9 (273)	3.00	1.71–5.26	< 0.01	3.37	1.89–6.01	< 0.01
*A. ceylanicum* co-infection		0.7 (49)	–	–	–	4.57	2.33–8.93	< 0.01
*S. stercoralis* co-infection		0.5 (35)	2.78	1.29–5.99	0.010	2.68	1.23–5.88	0.014
Usual place of defecation at school	Toilet/latrine	91.2 (6.390)	Reference			Reference		
	On the ground/in the bush/grass	5.5 (383)	1.35	1.00–1.83	0.048	1.42	1.05–1.93	0.023
	Doesn’t defecate at school	2.1 (147)	0.83	0.43–1.61	0.587	0.89	0.46–1.72	0.723
	Don’t know/refused	1.2 (85)	1.03	0.54–1.99	0.925	0.85	0.43–1.70	0.65
Household water supply^b^	Improved	85.1 (5.969)	Reference			Reference		
	Unimproved	13.1 (917)	1.32	1.07–1.62	< 0.01	1.28	1.04–1.57	0.022
	Don't know/refused	1.8 (128)	0.93	0.47–1.83	0.827	0.96	0.48–1.90	0.902
Household toilet available	No	24.8 (1.739)	Reference			Reference		
	Yes, but no flush	14.8 (1.040)	0.86	0.69–1.09	0.211	0.83	0.66–1.05	0.115
	Yes, flushes	60.4 (4.229)	0.58	0.48–0.71	< 0.01	0.58	0.47–0.70	< 0.01
Primary female carer's highest level of education	Never attended school	8.0 (562)	Reference			Reference		
	Completed or attended primary school	29.5 (2.072)	0.88	0.69–1.11	0.278	0.87	0.69–1.11	0.260
	Completed or attended secondary school	49.6 (3.484)	0.67	0.52–0.86	< 0.01	0.65	0.51–0.84	< 0.01
	Completed or attended tertiary education	9.2 (645)	0.36	0.22–0.59	< 0.01	0.39	0.24–0.63	< 0.01
	Don't know/refused	3.7 (262)	0.55	0.34–0.89	0.014	0.57	0.35–0.93	0.023

Models constructed for all hookworm (*N. americanus*, *A. duodenale* and *A. ceylanicum*) and *N. americanus* only, due to prevalence of all other soil-transmitted helminth species < 1%

*aOR* adjusted odds ratio, *95% CI* 95% confidence interval, *Reference* reference category for categorical variable

*Proportion of the total sample size that had data from all sources (demographic and school questionnaire), *N* = 7030

^†^Number of observations included in each multivariable model, not all 7030 observations included in the analysis due to missingness of specific variables (0.9% of cases had incomplete data for the predictors in these models and therefore not included in the analyses)

^a^Ethnicities where the number of students were less than 200 were grouped together under ‘other’ for risk factor analysis; see Additional file 1: Table S2 for a complete breakdown of student ethnicities

^b^Response for variable collapsed to align with WHO and United Nations International Children’s Emergency Fund (UNICEF) Joint Monitoring Programme (JMP) for Water Supply and Sanitation definitions. Improved = piped water, protected well, rainwater or public tap. Unimproved = unprotected well or surface water

Co-infection with *S. stercoralis* was associated with increased odds of both all hookworm and *N. americanus* (hookworm: aOR 2.78, 95% CI 1.29–5.99, *P* = 0.01; *N. americanus*: aOR 2.68, 95% CI 1.23–5.88, *P* = 0.014). Additionally, co-infection with *A. ceylanicum* was associated with increased odds of *N. americanus* infection (aOR 4.57, 95% CI 2.33–8.93, *P* < 0.01) (Table 3).

Children who reported that they practise open defecation at school were at increased odds of infection (hookworm: aOR 1.35, 95% CI 1.00–1.83, *P* = 0.048; *N. americanus* aOR 1.42, 95% CI 1.05–1.93, *P* = 0.023). Having an unimproved household water source was also associated with increased odds of infection compared to an improved water source (hookworm: aOR 1.32, 95% CI 1.07–1.62, *P* < 0.01; *N. americanus*: aOR 1.28, 95% CI 1.04–1.57, *P* = 0.022) (Table 4). Conversely, having access at home to a toilet/latrine that flushes compared to no toilet reduced the odds of infection for both outcomes (hookworm: aOR 0.58, 95% CI 0.48–0.71, *P* < 0.01; *N. americanus*: aOR 0.58, 95% CI 0.47–0.70, *P* < 0.01) (Table 3).

**Table 4:** Factors associated with moderate-to-heavy-intensity *Necator americanus* infection

Variable			Multivariate model (*N* = 1047^**†**^)		
		% (*n*)*	aOR	95% CI	*P*-value
Age		8.2 ± (1.36)	1.13	1.01–1.26	0.033
Female		43.6 (461)	1.13	0.84–1.52	0.426
Ethnicity^a^	Kinh	6.9 (73)	Reference		
	Dao	1.8 (19)	0.31	0.03–3.10	0.32
	Gia-rai	8.9 (94)	4.50	1.42–14.26	0.011
	Hmông	11.7 (124)	3.73	1.22–11.40	0.021
	Mnông	11.7 (124)	2.34	0.76–7.15	0.138
	Nùng	4.2 (44)	1.93	0.57–6.55	0.293
	Tày	2.1 (22)	2.63	0.59–11.61	0.203
	Xơ-đăng	4.9 (52)	5.06	1.46–17.56	0.011
	Êđê	45.7 (483)	3.14	1.28–7.72	0.013
	Other	2.2 (23)	3.40	0.87–13.22	0.078
School handwashing station	No	9.5 (99)	Reference		
	Yes, but no soap	70.8 (741)	0.30	0.14–0.65	0.002
	Yes, soap available	19.8 (207)	0.31	0.12–0.78	0.013

Factors investigated comparing moderate-to-heavy-intensity infection to light-intensity infection

*Proportion of the total sample size that had qPCR positive for *Necator americanus*, *N* = 1058

^**†**^Number observations included in final multivariable model, not all 1058 observations included due to missingness of specific variables (1% of cases had incomplete data for the predictors in these models and therefore not included in the analyses). Reference = reference category for categorical variable. Infection intensity classes are according to WHO guidelines for classifying infection intensity based on EPG values; Light-intensity infection: *N. americanus* (1–1999 EPG) moderate: *N. americanus* (2000–3999 EPG); heavy: *N. americanus* (≥ 4000 EPG). Moderate and heavy intensity analysed as a single group to align with WHO 2030 targets STH control programme monitoring targets

^a^Ethnicities where the number of students were less than 200 were grouped together under ‘other’ for risk factor analysis, see Additional file 1: Table S2 for a complete breakdown of student ethnicities

For both outcomes having a primary female caregiver who attended or completed secondary education or who attended or completed tertiary education compared to never attending school was associated with reduced odds of infection (hookworm: aOR 0.67, 95% CI 0.52–0.86, *P* < 0.01 and aOR 0.36, 95% CI 0.22–0.59, *P* < 0.01 respectively; *N. americanus*: aOR 0.65, 95% CI 0.51–0.84, *P* < 0.01 and aOR 0.39, 95% CI 0.23–0.63, *P* < 0.01 respectively (Table 3).

### Risk factors for moderate-to-heavy-intensity *Necator americanus* infection

We analysed risk factors for moderate-to-heavy-intensity *N. americanus* infection compared to light-intensity infection to identify potential factors that may be associated with increased odds of developing moderate-to-heavy-intensity infection in infected individuals. The results of the final multivariate model are summarised in Table 4 (See Additional file 1: Table S8).

Among qPCR-positive individuals, there was no difference in the odds of having moderate-to-heavy-intensity infection between females and males (aOR 1.13, 95% CI 0.83–1.53, *P* = 0.426) (Table 4). Increasing age was associated with increased odds of moderate-to-heavy-intensity infection (aOR 1.13, 95% CI 1.01–1.26, *P* = 0.033).

Compared to the Kinh ethnic group, children from several ethnic minority groups had increased odds of moderate-to-heavy-intensity infection: Gia-rai (aOR 4.50, 95% CI 1.42–14.26, *P* = 0.011), Hmông (aOR 3.73, 95% CI 1.22–11.40, *P* = 0.021), Xơ-đăng (aOR 5.06, 95% CI 1.46–17.56, *P* = 0.011) and Êđê (aOR 3.14, 95% CI 1.28–7.72, *P* = 0.013) (Table 4).

Having access to a handwashing station at school with soap (aOR 0.31 95% CI 0.12–0.748 *P* = 0.013) and without soap (aOR 0.30, 95% CI 0.14–0.65, *P* < 0.01), were associated with reduced odds of moderate-to-heavy-intensity infection compared to students who attended schools without a handwashing station (Table 4).

### Sensitivity analysis

A sensitivity analysis was conducted with the addition of another level of clustering, by adding hamlet as an additional random effects term. This included 6968 students for analysis, after excluding 62 participants that were missing data on hamlet. Results for the final multivariate model with respect to each outcome are summarised in Additional file 1: Tables S9, S10, while Additional file 1: Tables S11, S12, S13 summarise the univariate analysis and domain multivariate analysis. Adjusting for clustering at the school and hamlet level resulted in a final multivariate model that was very similar for the outcomes of all hookworm and *N. americanus*, compared to adjusting for school alone (Additional file 1: Table S9). Except for the exclusion of the variable ‘usual place of defecation at school’ from both models, all other included covariates were the same and produced similar estimated effects (aOR) and 95% CI. For the analysis of factors associated with moderate-to-heavy-intensity *N. americanus* infection, adjusting for clustering at the school and hamlet level made no difference to the model (Additional file 1: Table S10).

## Discussion

To our knowledge, this study conducted in 7710 primary school-age children in 64 schools across Dak Lak province is the largest qPCR-based STH prevalence survey conducted to date [46, 48]. While the overall prevalence of *N. americanus* was under 15%, a quarter of surveyed schools and a number of ethnic minority groups had a prevalence of 20% or greater. Additionally, the prevalence of moderate-to-heavy-intensity infections was above WHO’s target of 2% for achieving ‘elimination of STH as a public health problem’ [8]. These findings suggest that some communities in Dak Lak province require additional efforts to control STH.

Dak Lak province has had large-scale targeted deworming of primary school-age children with the anthelminthic drugs mebendazole or albendazole in place since 2007 [32]. Albendazole is highly efficacious against *A. lumbricoides* and hookworm species; however, mebendazole has much lower efficacy against hookworm than albendazole, while maintaining good efficacy against *A. lumbricoides* [49]. Sustained regular mass deworming in the past may have contributed to the observation that hookworm is now more common than *Ascaris* spp. due to the wide use of mebendazole [30]. The finding that most infections (and all moderate-to-heavy-intensity infections) were due to *N. americanus* suggests that albendazole rather than mebendazole should be the anthelminthic of choice for future STH control programmes in the region [49]. The low prevalence of *S. stercoralis* would suggest that ivermectin may not be needed in addition to albendazole [8]. Moreover, the low prevalence of zoonotic hookworm *A. ceylanicum* suggests that STH control efforts can focus on interventions targeted at humans [50].

With respect to associations between risk factors and STH infection, we found female sex to be protective, with lower odds of *N. americanus* infection, as reported by other studies, but no such effect was found with the odds of moderate-to-heavy-intensity compared to light-intensity *N. americanus* infection [46]. This has been hypothesised to be related to behaviour and exposure risk, rather than biological differences in infection susceptibility [7]. Additionally, children whose primary female carer attended secondary school or above, compared to never attending school, had lower odds of *N. americanus* infection, consistent with other studies [46, 51].

Having an improved household water source and having a flushing household toilet/latrine were both found to be associated with reduced odds of *N. americanus* infection. Conversely, practising open defecation at school was associated with increased odds of infection with *N. americanus*. When examining WASH factors associated with the risk of moderate-to-heavy-intensity versus light-intensity *N. americanus* infection, we found the odds were significantly reduced in children who had access to a handwashing station with water at school, with or without the availability of soap. These factors have been shown in other observational studies to be associated with STH infection [18, 19, 52]. We did not find an association between shoe-wearing and the odds of *N. americanus* infection, which would be biologically plausible given that hookworms are transmitted by direct skin penetration of larvae residing in the soil [53]. This lack of association may be due to high self-reported rates of children wearing shoes when outside (> 70%).

The associations between WASH factors and infection in this survey are biologically plausible with respect to the current understanding of STH transmission [54] given that the aim of improved WASH is to separate faeces from the surrounding environment, reducing human exposure and infection transmission [10]. However, many observational studies tend to produce mixed results with respect to associations between WASH and STH infection [10]. The large sample size and the use of qPCR, a more sensitive diagnostic method, in this survey may have allowed us to identify associations more accurately.

These findings indicate that, in addition to preventive chemotherapy programmes, other elements can be included to improve STH control programmes. Facilitating access to improved water supplies and flushing toilets at home requires significant infrastructure that may be beyond the reaches of public health programmes; however, education programmes focusing on promoting the end of open defecation and improving hand hygiene in schoolchildren may help reduce STH transmission and disease burden.

An important finding in the risk factor analysis was the increased odds of infection with *N. americanus* associated with belonging to an ethnic minority group (Gia-rai, Hmong, Xo-dang, Êđê, Dao, Mnông, Nùng, Tày and other) compared to children from the ethnic majority group in Vietnam (Kinh), while some ethnic minorities (Gia-rai, Hmong, Xo-dang and Ede) were also associated with increased odds of moderate-to-heavy-intensity *N. americanus* infection. The burden of STH has been shown to be high amongst indigenous ethnic minority groups throughout the Western Pacific and neighbouring South-East Asia region, namely in the Philippines and Malaysia [4, 55, 56]. There has been one previous study comparing the prevalence of STH between ethnic groups in Vietnam, which found high prevalence in both Kinh and ethnic minority groups [57]. However, this study was conducted in northern Vietnam and prior to the scaling up of preventive chemotherapy programmes [31, 57]. The disparity seen in infection prevalence between ethnic minority groups and the ethnic majority group in this survey could be explained by multiple factors. In Vietnam, ethnic minority communities have lower levels of income and education attainment, and poorer access to health care, potable water and sanitation facilities—all factors which may contribute to increased odds of infection with STH [58]. However, this association was present even when adjusting for the education level of primary female carers and WASH factors in our multivariate model. Therefore, we hypothesise that there are likely additional factors not measured in this analysis, such as behavioural, socioeconomic or geographical factors, that may be increasing exposure to STH infectious stages, reducing access to ongoing STH control programmes, or resulting in their poor acceptability by these communities. Additionally, STHs are focally distributed and transmission is in part driven by local environmental factors, which we have also not accounted for in our analysis [58]. Regardless, these findings help characterise the higher burden of STH infection in Vietnamese ethnic minorities. This highlights that further research is needed to better understand the underlying epidemiological drivers in different ethnic groups in Vietnam, which should include qualitative studies to evaluate barriers to accessing and engaging in STH control programmes (Additional file 2).

There are some important limitations to keep in mind when considering the reported findings. The WASH characteristics and socioeconomic factors were collected by self-reported questionnaires. Such data are prone to recall and social desirability bias and may influence the association or non-association of these factors on the outcome of STH infection [11].

In addition, although qPCR has been demonstrated to be a highly specific and sensitive technique for STH diagnosis [21, 22, 24, 59–61], there is still work needed to improve its use for informing decisions around STH control, particularly to standardise qPCR approaches and validate estimates of prevalence and intensity against established targets generated using Kato-Katz microscopy [8]. In particular, while there have been studies estimating EPG from Ct values using conversion equations derived from seeding experiments [27, 28], further work is needed to determine whether the threshold of 2% prevalence of high and moderate infections needs to be adjusted when using qPCR. Geostatistical analysis that includes environmental variables would be an important next step beyond this current analysis, to better understand the drivers of STH epidemiology and explain the observed spatial variability in Dak Lak province. Our statistical approach using mixed-effects logistic regression to allow for sample clustering for the risk factor analysis is similar to previous STH risk factor analyses [14, 37, 46]. Recently published work has identified other statistical approaches such as Bayesian networks and recursive partitioning as potentially superior methods for evaluating risk factors for STH infection [17]. These methods may have several benefits over mixed-effects logistic regression, but importantly do not rely on the assumption of independence between predictor variables, an assumption which is typically violated for WASH, demographic and socioeconomic variables commonly evaluated in STH studies [17, 62].

## Conclusion

In conclusion, in this largest STH survey using qPCR to date, we found that school-age children from ethnic minorities had significantly higher odds for both prevalence and intensity of *N. americanus* infection compared to ethnic majority children. Additionally, we found evidence of access to improved WASH infrastructure and behaviour, and higher levels of primary female carer education to be protective against *N. americanus* infection for school-age children in Dak Lak province. These findings highlight that control programmes need to be tailored to specific communities to effectively achieve STH control and reduce morbidity.

## Supplementary Information

**Additional file 1.** Supplementary figure 1 and supplementary tables 1–13.

**Additional file 2.** STROBE checklist—reporting checklist for cross-sectional studies.

## Data Availability

Restrictions will apply to the data used in this study. All relevant aggregated data are within the manuscript and supporting files. Individual-level data cannot be made publicly available to respect participant privacy and confidentiality in accordance with the study protocol approved by the research ethics board. Researchers may request access to the data from the Human Research Ethics Committees at the University of New South Wales (umanethics@unsw.edu.au) or Tay Nguyen University (https://www.ttn.edu.vn/).

## Abbreviations

AIC: Akaike information criteria
aOR: Adjusted odds ratio
CI: Confidence interval
CoDe-STH: Community deworming against soil-transmitted helminths
Ct: Cycle-threshold
EPG: Eggs per gram
NTD: Neglected tropical disease
qPCR: Quantitative polymerase chain reaction
STH: Soil-transmitted helminths
USD: United States dollar
WHO: World Health Organization
VIF: Variance inflation factor
VND: Vietnamese dong
WASH: Water, sanitation and hygiene
YLD: Years lived with disability
