# Supplementary material for: Epidemiology of soil-transmitted helminths using quantitative PCR and risk factors for hookworm and Necator americanus infection in school children in Dak Lak province, Vietnam
Source: Parasit Vectors. 2023 Jun 27;16:213. doi: 10.1186/s13071-023-05809-x (PMC10304358; doi:10.1186/s13071-023-05809-x)
Supplement: Supplementary file 1 — Additional file 1. Supplementary figure 1 and supplementary tables 1–13. [file 13071_2023_5809_MOESM1_ESM.docx]

**S1. Supplementary figure 1 and supplementary tables 1 – 13.**

**List of supplementary tables and**

**Supplementary figure 1.** Flowchart describing participants included in analysis

**Supplementary table 1.** Participant characteristics

**Supplementary table 2.** Summary of ethnicity of all participants

**Supplementary table 3.** Selected characteristics stratified by provision of stool sample and qPCR performed on stool sample

**Supplementary table 4.** Clustered-adjusted STH prevalence stratified by ethnicity

**Supplementary table 5.** Clustered-adjusted STH prevalence stratified by district

**Supplementary table 6.** Factors associated with all hookworm infection univariate analysis and multivariate analysis by domain

**Supplementary table 7.** Factors associated with *Necator americanus*, univariate and domain level multivariate analysis

**Supplementary table 8.** Factors associated with moderate-to-heavy intensity *Necator Americanus* infection: univariate analysis and multivariate analysis by domain

**Supplementary table 9.** Sensitivity analysis adjusting for clustering at school and hamlet level: Factors associated with all hookworm and *Necator americanus* infection

**Supplementary table 10.** Sensitivity analysis adjusting for clustering at school and hamlet level: Factors associated with moderate-to-heavy intensity *Necator americanus* infection

**Supplementary table 11.** Sensitivity analysis adjusting for clustering at school and hamlet level: Factors associated with all hookworm, univariate and domain level multivariate analysis

**Supplementary table 12.** Sensitivity analysis adjusting for clustering at school and hamlet level: Factors associated with *Necator americanus*, univariate and domain level multivariate analysis

**Supplementary table 13.** Sensitivity analysis adjusting for clustering at school and hamlet level: Factors associated with moderate-to-heavy intensity *N. americanus* infection, univariate and domain level multivariate

14,116 students enrolled to participate

(Mean number of students per school/cluster = 221, range = 129-374)

8,730 provided stool samples

(Mean number of students per school/cluster = 136, range = 70-219)

9,698 completed a student questionnaire

(Mean number of students per school/cluster = 151 range = 106-289)

10,752 had a completed caregiver questionnaire

(Mean number of students per school/cluster = 168, range = 104-289)

7,710 individual student stool samples underwent qPCR to detect for presence of STH

(Mean number of students per school/cluster = 130, range = 70-135*)

7,030 students had a stool sample with qPCR and had an individual and caregiver questionnaires

Risk factors analysis was conducted using complete case-analysis

(Mean number of students per school/cluster = 110, range = 65-127)

(Refer to results tables for exact number of total individuals included in each multivariate model)

Supplementary figure 1. Flowchart describing participants included in analysis. *Nine schools had less than 120 stool samples submitted. In these nine schools all submitted samples were analysed for presence of STH by qPCR. Five schools submitted 120 or more stool samples but had less than 120 samples analysed by qPCR.

| **Supplementary table 1.** Participant characteristics, total N=7,030 | | | | |
| --- | --- | --- | --- | --- |
| **Variable** |  | **%** | | **n** |
| ***Demographics*** |  |  | |  |
| Age |  | 7.9 years | | ±1.23 |
| Female |  | 51.37 | | (3,611) |
| Ethnicity**^a^** | Kinh | 39.70 | | (2,791) |
|  | Êđê | 29.20 | | (2,053) |
|  | Dao | 2.03 | | (143) |
|  | Gia-rai | 3.20 | | (225) |
|  | Hmông | 3.34 | | (235) |
|  | Mnông | 5.72 | | (402) |
|  | Nùng | 5.85 | | (411) |
|  | Tày | 5.87 | | (413) |
|  | Xơ-đăng | 1.19 | | (84) |
|  | Other | 3.88 | | (273) |
| School grade | Grade 1 | 26.24 | | (1845) |
|  | Grade 2 | 27.98 | | (1967) |
|  | Grade 3 | 23.41 | | (1646) |
|  | Grade 4 | 22.35 | | (1571) |
| ***Student anti-helminthic status, hygiene, and defecation behaviour*** | |  | |  |
| Received anti-helminthic in last year | | 45.61 | | (3,202) |
| Uses household latrine**^b^** | Always | 80.27 | | (4,243) |
|  | Sometimes | 18.63 | | (985) |
|  | Never | 0.83 | | (44) |
|  | Don’t know/refused | 0.11 | | (6) |
| Usual place of defecation at home | Toilet/latrine | 75.91 | | (5326) |
|  | On the ground/bushes outside | 0.95 | | (67) |
|  | Neighbour toilet/latrine | 0.31 | | (22) |
|  | Village toilet/latrine | 21.81 | | (1530) |
|  | Other | 0.74 | | (52) |
|  | Don't know/refused | 0.27 | | (19) |
| Ever defecates outside at home**^c^** | | 46.31 | | (3,235) |
| Usual place of defecation at school | School toilet/latrine | 91.22 | | (6390) |
|  | On the ground or in the bushes/grass | 5.47 | | (383) |
|  | Other | 2.10 | | (147) |
|  | Don't know/refused | 1.21 | | (85) |
| Ever defecates outside at school**^c^** | | 14.22 | | (993) |
| Wears shoes outside | Always | 72.25 | | (5,071) |
|  | Sometimes | 25.89 | | (1,817) |
|  | Never | 1.71 | | (120) |
|  | Don't know/refused | 0.16 | | (11) |
| Wears shoes defecating | Always | 66.95 | | (4,702) |
|  | Sometimes | 18.50 | | (1,299) |
|  | Never | 13.77 | | (967) |
|  | Don't know/refused | 0.78 | | (55) |
| Wash hands after defecating | Always | 59.72 | | (4,192) |
|  | Sometimes | 34.12 | | (2,395) |
|  | Never | 6.04 | | (424) |
|  | Don't know/refused | 0.11 | | (8) |
| Wash hands before eating | Always | 51.55 | | (3,614) |
|  | Sometimes | 42.35 | | (2,969) |
|  | Never | 5.98 | | (419) |
|  | Don't know/refused | 0.13 | | (9) |
| ***Household WASH*** | | | | |
| Household water source | Piped water to house or yard | 5.98 | (420) | |
|  | Protected dug well | 2.79 | (196) | |
|  | Public tap | 71.87 | (5051) | |
|  | Rainwater | 10.73 | (754) | |
|  | Surface water | 1.69 | (119) | |
|  | Unprotected well | 1.88 | (132) | |
|  | Other | 3.86 | (271) | |
|  | Don't know/refused | 1.21 | (85) | |
| Household has latrine |  | 75.25 | (5,286) | |
| Household latrine flushes**^d^** |  | 80.16 | (4,237) | |
| Handwashing station at home |  | 91.14 | (6,377) | |
| Soap at home |  | 68.92 | (4,836) | |
| ***Household socioeconomic*** | | | | |
| Household income | Less than 20,000,000 VND | 45.01 | | (3,161) |
|  | VND 20,000,000 – 50,000,000 | 26.04 | | (1,829) |
|  | VND 50,000,000 – 200,000,000 | 7.22 | | (507) |
|  | More than VND 200,000,000 | 0.64 | | (45) |
|  | Don't know/refused | 21.09 | | (1,481) |
| Occupation main household income earner | Farmer | 91.20 | | (6,407) |
|  | Animal keeper | 1.04 | | (73) |
|  | Clerk/administration | 1.11 | | (78) |
|  | Fisherman | 0.26 | | (18) |
|  | Health worker | 0.54 | | (38) |
|  | Selling at market | 1.74 | | (122) |
|  | Unemployed | 0.50 | | (35) |
|  | Other | 2.56 | | (180) |
|  | Don't know/refused | 1.05 | | (74) |
| Primary female carer's highest education level completed | Never attended school | 8.00 | | (562) |
|  | Attended primary school but did not complete | 14.59 | | (1,025) |
|  | Completed primary school | 14.90 | | (1,047) |
|  | Attended secondary school but did not complete | 16.80 | | (1,180) |
|  | Completed secondary school | 32.80 | | (2,304) |
|  | Attended university but did not complete | 1.12 | | (79) |
|  | Completed university | 2.80 | | (197) |
|  | Completed professional training | 5.25 | | (369) |
|  | Don't know/refused | 3.73 | | (262) |
| ***School location and WASH*^d^** | | | | |
| Location | Rural | 56.25 | | (36) |
|  | Remote | 32.81 | | (21) |
|  | Very remote | 10.94 | | (7) |
| School water source | No water | 28.12 | | (18) |
|  | Piped water | 34.38 | | (22) |
|  | Protected dug well | 7.81 | | (5) |
|  | Unprotected well | 14.06 | | (9) |
|  | Don't know/refused | 15.62 | | (10) |
| Latrine/toilet availble for students to use**^e^** | Yes | 96.88 | | (62) |
|  | No, toilets broken/pitfull/dirty | 1.56 | | (1) |
|  | No, toilets for teachers only | 1.56 | | (1) |
| Has flush toilet |  | 89.06 | | (57) |
| Has pit latrine |  | 17.19 | | (11) |
| Flushes with water (offset pit)**^f^** |  | 72.73 | | (8) |
| Does not flush (direct pit)**^f^** |  | 27.27 | | (3) |
| Has a slab**^f^** |  | 72.73 | | (8) |
| School latrine/toilet observed to be/have**^g^** | Locked | 6.25 | | (4) |
|  | Clean | 59.38 | | (38) |
|  | Water for cleaning self | 48.44 | | (31) |
|  | Dirty | 31.25 | | (20) |
|  | Flies | 15.62 | | (10) |
| Handwashing station available |  | 88.89 | | (56) |
| Soap available**^h^** |  | 35.71 | | (20) |
| Number of school toilets for students to use | 0 | 3.13 | | (2) |
|  | 1 to 5 | 62.50 | | (40) |
|  | 6 to 10 | 28.13 | | (18) |
|  | Greater than 10 | 6.25 | | (4) |
| Number of school toilets for girls | 1 to 5 | 91.94 | | (57) |
|  | 6 to 10 | 6.25 | | (4) |
|  | Greater than 10 | 1.56 | | (1) |
| Number of school toilets for boys | 1 to 5 | 96.83 | | (61) |
|  | 6 to 10 | 1.56 | | (1) |
|  | Greater than 10 | 1.56 | | (1) |
| **Notes:** **^a^**See Supplementary table 1 for list of ethnicities grouped in 'Other'. **^b^**Responses only recorded for those who responded yes to "Household has latrine", percentage out of N=5286. **^c^**Refers to ever reporting defecating outside at home or school, i.e. practices open defecation. **^d^**Percentages reported out of number of schoos, N=64. **^e^**All schools reported having at least one latrine/toilet for students. **^f^**Characteristics only reported for schools that reported having a pit latrine, percentage out of N=11. **^g^**Observations made by research team members for toilets/latrines at school. **^h^**Question only answered, if answered yes to Handwashing station available, percentage out of N=56. | | | | |

| **Supplementary table 2.** Summary of ethnicity of all participants | |
| --- | --- |
| **Ethnicity** | **Number of participants** |
| Ba Na | 20 |
| Cao Lan | 98 |
| Chứt | 32 |
| Dao | 336 |
| Gia-rai | 509 |
| Hmông | 549 |
| Kinh | 5,342 |
| Mnông | 903 |
| Mường | 138 |
| Nùng | 753 |
| Thái | 143 |
| Tày | 730 |
| Xơ-đăng | 331 |
| Êđê | 4,188 |
| Other^a^ | 44 |
| ^a^All ethnicities where student numbers were less than 10 included in ‘other’ to protect participant confidentiality | |

| **Supplementary table 3.** Selected characteristics stratified by provision of stool sample and qPCR performed on stool sample | | | | | | | | | | |
| --- | --- | --- | --- | --- | --- | --- | --- | --- | --- | --- |
| ***Demographics^*^*** | | | | | | | | | | |
|  |  | stool sample | | | |  | qPCR performed on stool sample | | | |
|  |  | Yes | | No | |  | Yes | | No | |
| *Number of participants with data* | | N=8730 | | N=5386 | |  | N=7710 | | N=1020 | |
|  |  | mean (SD) | | | |  | mean (SD) | | |  |
| Age (years) |  | 7.9 | (1.24) | 7.8 | (1.30) |  | 7.9 | (1.23) | 8.1 | (1.27) |
|  |  | % (n) | | | |  | % (n) | | | |
| Female |  | 50.94 | (4447) | 42.57 | (2293) |  | 51.15 | (3944) | 49.31 | (503) |
| Ethnicity**^a^** | Kinh | 40.02 | (3494) | 34.31 | (1848) |  | 40.44 | (3118) | 36.86 | (376) |
|  | Êđê | 28.26 | (2467) | 31.95 | (1721) |  | 28.37 | (2187) | 27.45 | (280) |
|  | Dao | 2.29 | (200) | 2.53 | (136) |  | 2.23 | (172) | 2.75 | (28) |
|  | Gia-rai | 3.88 | (339) | 3.16 | (170) |  | 3.18 | (245) | 9.22 | (94) |
|  | Hmông | 3.32 | (290) | 4.81 | (259) |  | 3.20 | (247) | 4.22 | (43) |
|  | Mnông | 5.10 | (445) | 8.50 | (458) |  | 5.62 | (433) | 1.18 | (12) |
|  | Nùng | 6.11 | (533) | 4.08 | (220) |  | 5.89 | (454) | 7.75 | (79) |
|  | Tày | 5.90 | (515) | 3.99 | (215) |  | 5.97 | (460) | 5.40 | (55) |
|  | Xơ-đăng | 1.20 | (105) | 4.20 | (226) |  | 1.26 | (97) | 0.79 | (8) |
|  | Other | 3.92 | (342) | 2.47 | (133) |  | 3.85 | (297) | 4.42 | (45) |
| School grade | Grade 1 | 25.91 | (2262) | 30.49 | (1642) |  | 26.13 | (2015) | 24.22 | (247) |
|  | Grade 2 | 26.92 | (2350) | 27.81 | (1498) |  | 27.56 | (2125) | 22.06 | (225) |
|  | Grade 3 | 24.10 | (2104) | 22.67 | (1221) |  | 23.97 | (1848) | 25.09 | (256) |
|  | Grade 4 | 23.05 | (2012) | 19.03 | (1025) |  | 22.31 | (1720) | 28.63 | (292) |
|  |  |  |  |  |  |  |  |  |  |  |
| ***Health, hygiene and household sanitation^†^*** | | | | | | | | | | |
|  |  | Baseline stool sample in 2019 | | | |  | qPCR performed on baseline stool sample | | | |
|  |  | Yes | | No | |  | Yes | | No | |
| *Number of participants with data* | | N=8298 | | N=1293 | |  | N=7327 | | N=971 | |
|  |  | % (n) | | % (n) | |  | % (n) | | % (n) | |
| Had deworming in last year | | 46.01 | (3818) | 36.54 | (509) |  | 45.54 | (3332) | 50.10 | (486) |
| Household has latrine | | 74.98 | (6222) | 73.51 | (1024) |  | 75.34 | (5520) | 72.30 | (702) |
| Household latrine flushes**^c^** | | 80.07 | (4982) | 86.62 | (887) |  | 80.34 | (4435) | 77.92 | (547) |
| Uses household latrine**^c^** | Always | 60.20 | (4995) | 57.07 | (795) |  | 24.77 | (1815) | 27.60 | (268) |
|  | Sometimes | 13.99 | (1161) | 15.08 | (210) |  | 14.03 | (1028) | 13.70 | (133) |
|  | Never | 0.63 | (52) | 0.57 | (8) |  | 0.60 | (44) | 0.82 | (8) |
| Usual place of defecation at home**^b^** | Toilet/latrine | 75.61 | (6274) | 73.30 | (1021) |  | 76.15 | (5569) | 72.83 | (705) |
|  | On the ground/bushes outside | 21.85 | (1813) | 22.40 | (312) |  | 21.58 | (1578) | 24.28 | (235) |
|  | Neighbour toilet/latrine | 1.02 | (85) | 2.63 | (34) |  | 0.96 | (70) | 1.54 | (15) |
|  | Village toilet/latrine | 0.34 | (28) | 0.54 | (7) |  | 0.31 | (23) | 0.51 | (5) |
|  | Other | 0.71 | (59) | 0.93 | (12) |  | 0.74 | (54) | 0.51 | (5) |
|  | Don't know/refused | 0.27 | (22) | 0.31 | (4) |  | 0.26 | (19) | 0.31 | (3) |
| Ever defecates outside at home**^d^** | | 46.16 | (3830) | 52.44 | (678) |  | 45.87 | (3361) | 48.30 | (469) |
| Usual place of defecation at school | School toilet/latrine | 91.01 | (7552) | 87.08 | (1213) |  | 90.84 | (6656) | 92.28 | (896) |
|  | ground/in the bush/grass | 5.41 | (449) | 8.04 | (112) |  | 5.42 | (397) | 5.36 | (52) |
|  | Other | 2.10 | (174) | 2.66 | (37) |  | 2.17 | (159) | 1.54 | (15) |
|  | Don't know/refused | 1.14 | (95) | 1.55 | (20) |  | 1.20 | (88) | 0.72 | (7) |
| Ever defecates outside at school**^d^** | | 14.04 | (1165) | 21.35 | (276) |  | 14.00 | (1026) | 14.32 | (139) |
| Handwashing station at home | | 90.53 | (7512) | 91.82 | (1279) |  | 91.18 | (6649) | 89.15 | (863) |
| Soap at home | | 68.66 | (5697) | 66.40 | (925) |  | 68.92 | (5041) | 67.70 | (656) |
| Wears shoes outside | Always | 72.79 | (6040) | 70.64 | (984) |  | 72.25 | (5294) | 76.83 | (746) |
|  | Sometimes | 25.20 | (2091) | 26.92 | (375) |  | 25.73 | (1885) | 21.22 | (206) |
|  | Never | 1.72 | (143) | 2.08 | (29) |  | 1.72 | (126) | 1.75 | (17) |
|  | Don't know/refused | 0.13 | (11) | 0.31 | (4) |  | 0.15 | (11) | 0.00 | (0) |
| Wears shoes defecating | Always | 66.57 | (5524) | 65.40 | (911) |  | 66.92 | (4898) | 64.67 | (626) |
|  | Sometimes | 18.87 | (1566) | 22.76 | (317) |  | 18.43 | (1349) | 22.42 | (217) |
|  | Never | 13.68 | (1135) | 11.20 | (156) |  | 13.90 | (1017) | 12.19 | (118) |
|  | Don't know/refused | 0.75 | (62) | 0.62 | (8) |  | 0.75 | (55) | 0.72 | (7) |
| Wash hands after defecating | Always | 60.27 | (5001) | 55.49 | (773) |  | 59.92 | (4383) | 63.78 | (618) |
|  | Sometimes | 33.63 | (2791) | 35.10 | (489) |  | 34.03 | (2489) | 31.17 | (302) |
|  | Never | 5.82 | (483) | 8.69 | (121) |  | 5.95 | (435) | 4.95 | (48) |
|  | Don't know/refused | 0.11 | (9) | 0.06 | (3) |  | 0.11 | (8) | 0.10 | (1) |
| Wash hands before eating | Always | 51.92 | (4308) | 51.47 | (717) |  | 51.88 | (3791) | 53.30 | (517) |
|  | Sometimes | 41.81 | (3469) | 40.92 | (570) |  | 42.01 | (3070) | 41.13 | (399) |
|  | Never | 5.89 | (489) | 7.18 | (100) |  | 5.97 | (436) | 5.46 | (53) |
|  | Don't know/refused | 0.13 | (11) | 0.31 | (4) |  | 0.14 | (10) | 0.10 | (1) |
| ***Household socioeconomic factors and water supply^**^*** | | | | | | | | | | |
|  |  | Baseline stool sample in 2019 | | | |  | qPCR performed on baseline stool sample | | | |
|  |  | Yes | | No | |  | Yes | | No | |
| *Number of participants with data* | | N=8294 | | N=2451 | |  | N=7341 | | N=953 | |
|  |  | % (n) | | % (n) | |  | % (n) | | % (n) | |
| Household income | Less than 20,000,000 VND | 45.49 | (3773) | 45.29 | (1110) |  | 44.91 | (3293) | 50.37 | (480) |
|  | VND 20,000,000 – 50,000,000 | 25.93 | (2151) | 26.44 | (648) |  | 26.20 | (1921) | 24.13 | (230) |
|  | VND 50,000,000 – 200,000,000 | 6.78 | (562) | 8.00 | (196) |  | 7.05 | (517) | 4.72 | (45) |
|  | More than VND 200,000,000 | 0.58 | (48) | 0.90 | (22) |  | 0.64 | (47) | 0.10 | (1) |
|  | Don't know/refused | 21.12 | (1752) | 19.34 | (474) |  | 21.18 | (1555) | 20.67 | (197) |
| Occupation main household income earner**^b^** | Farmer | 91.38 | (7579) | 91.43 | (2241) |  | 91.32 | (6699) | 92.34 | (880) |
|  | Animal keeper | 1.10 | (91) | 0.98 | (24) |  | 1.09 | (80) | 1.15 | (11) |
|  | Clerk/ administration | 1.13 | (94) | 0.98 | (24) |  | 1.10 | (81) | 1.36 | (13) |
|  | Fisherman | 0.25 | (21) | 0.20 | (5) |  | 0.26 | (19) | 0.21 | (2) |
|  | Health worker | 0.54 | (45) | 0.53 | (13) |  | 0.55 | (40) | 0.52 | (5) |
|  | Selling at market | 1.74 | (144) | 1.43 | (35) |  | 1.68 | (123) | 2.20 | (21) |
|  | Unemployed | 0.46 | (38) | 0.41 | (10) |  | 0.49 | (36) | 0.21 | (2) |
|  | Never went to school | 8.34 | (692) | 9.18 | (225) |  | 8.02 | (588) | 10.92 | (104) |
|  | Other | 2.35 | (195) | 3.02 | (74) |  | 2.48 | (182) | 1.36 | (13) |
|  | Don't know/refused | 0.99 | (82) | 0.98 | (24) |  | 1.04 | (76) | 0.63 | (6) |
| Primary female carer's highest education level completed | Attended primary school but did not complete | 14.60 | (1211) | 14.73 | (361) |  | 14.69 | (1078) | 13.97 | (133) |
|  | Completed primary school | 14.82 | (1229) | 15.34 | (376) |  | 14.76 | (1083) | 15.34 | (146) |
|  | Attended secondary school but did not complete | 16.72 | (1387) | 16.69 | (409) |  | 16.90 | (1240) | 15.44 | (147) |
|  | Completed secondary school | 32.78 | (2719) | 31.29 | (767) |  | 32.81 | (2407) | 32.77 | (312) |
|  | Attended university but did not complete | 1.07 | (89) | 0.86 | (21) |  | 1.12 | (82) | 0.74 | (7) |
|  | Completed university | 2.72 | (226) | 3.22 | (79) |  | 2.73 | (200) | 2.73 | (26) |
|  | Completed professional training | 5.12 | (425) | 4.73 | (116) |  | 5.25 | (385) | 4.20 | (40) |
|  | Don't know/refused | 3.74 | (310) | 3.75 | (92) |  | 3.72 | (273) | 3.88 | (37) |
| Household water source | Piped water to house or yard | 6.28 | (521) | 4.98 | (122) |  | 6.04 | (443) | 8.18 | (78) |
|  | Protected dug well | 71.23 | (5908) | 71.15 | (1744) |  | 71.98 | (5281) | 65.79 | (627) |
|  | Public tap | 2.76 | (229) | 2.73 | (67) |  | 2.74 | (201) | 2.94 | (28) |
|  | Rainwater | 1.71 | (142) | 1.39 | (34) |  | 1.68 | (123) | 1.99 | (19) |
|  | Surface water | 2.19 | (182) | 3.30 | (81) |  | 1.89 | (139) | 4.51 | (43) |
|  | Unprotected well | 10.80 | (896) | 12.81 | (314) |  | 10.67 | (783) | 11.86 | (113) |
|  | Other | 3.79 | (314) | 2.33 | (57) |  | 3.79 | (278) | 3.78 | (36) |
|  | Don't know/refused | 1.18 | (98) | 1.26 | (31) |  | 1.21 | (89) | 0.94 | (9) |
| **Notes: ^a^**See Supplementary table 1 for list of ethnicities grouped in 'Other'. **^b^**Responses only recorded for those who responded yes to "Household has latrine". **^c^**Refers to ever reporting defecating outside at home or school, i.e. practices open defecation. | | | | | | | | | | |

| **Supplementary table 4.** Clustered-adjusted STH prevalence stratified by ethnicity | | | | | | | | | | |
| --- | --- | --- | --- | --- | --- | --- | --- | --- | --- | --- |
| **Ethnicity** | **Study sample** | **Any STH^a^** | **Hookworm all** | ***N. americanus*** | ***A. ceylanicum*** | ***A. duodenale*** | ***A. lumbricoides*** | ***T. trichiura*** | ***S. stercoralis*** | **All evaluated STH^b^** |
|  | % | % | % | % | % | % | % | % | % |  |
|  | (n) | 95% CI | 95% CI | 95% CI | 95% CI | 95% CI | 95% CI | 95% CI | 95% CI |  |
| Kinh | 40.45 | 3.32 | 2.63 | 2.60 | 0.32 | 0.033 | 0.10 | 0.28 | 0.06 | 3.39 |
|  | (3119) | (2.46, 4.18) | (1.89, 3.64) | (1.83, 3.37) | (0.12, 0.52) | (0.00, 0.10) | (0.00, 0.24) | (0.06, 0.50) | (0.00, 0.15) | (2.52, 4.26) |
| Êđê | 28.36 | 24.22 | 22.73 | 22.50 | 0.87 | 0.18 | 0.40 | 1.05 | 0.73 | 24.24 |
|  | (2187) | (20.43, 28.02) | (18.81, 27.19) | (18.64, 26.36) | (0.47, 1.27) | (0.00, 0.38) | (0.02, 0.78) | (0.21, 1.90) | (0.37, 1.09) | (20.47, 28.00) |
| Dao | 2.23 | 15.31 | 11.63 | 13.63 | 1.71 | 0 | 0 | 2.31 | 0 | 15.17 |
|  | (172) | (8.58, 22.03) | (6.98, 18.75) | (7.12, 20.14) | (0.00, 3.67) |  |  | (0.00, 6.00) |  | (8.51, 21.83) |
| Gia-rai | 3.18 | 26.45 | 38.37 | 24.31 | 0.42 | 0 | 0.56 | 2.12 | 2.45 | 27.37 |
|  | (245) | (16.51, 36.38) | (18.83, 62.65) | (14.45, 34.17) | (0.00, 1.24) |  | (0.00, 2.01) | (0.00, 5.33) | (0.51, 4.38) | (17.30, 37.44) |
| Hmông | 3.20 | 45.50 | 51.01 | 43.17 | 1.63 | 0 | 0.28 | 0 | 1.62 | 46.54 |
|  | (247) | (3.54, 55.64) | (44.12, 57.87) | (32.82, 53.52) | (0.00, 3.27) |  | (0.00, 0.96) |  | (0.045, 3.19) | (36.40, 56.68) |
| Mnông | 5.62 | 24.16 | 29.10 | 19.74 | 1.84 | 0 | 1.68 | 1.46 | 1.85 | 25.83 |
|  | (433) | (16.08, 32.23) | (19.27, 41.37) | (12.06, 27.43) | (0.53, 3.16) |  | (0.00, 4.03) | (0.00, 3.07) | (0.58, 3.12) | (17.54, 34.11) |
| Nùng | 5.89 | 10.41 | 10.13 | 9.54 | 0.43 | 0 | 0 | 0.65 | 0.22 | 10.44 |
|  | (454) | (6.95, 13.87) | (7.11, 14.25) | (6.16, 12.92) | (0.00, 1.04) |  |  | (0.00, 1.67) | (0.00, 0.65) | (6.98 13.89) |
| Tày | 5.97 | 6.06 | 5.22 | 5.45 | 0.65 | 0 | 0 | 0.27 | 0.43 | 6.62 |
|  | (460) | (3.41, 8.70) | (3.62, 7.46) | (2.88, 8.02) | (0.00, 1.39) |  |  | (0.00, 0.84) | (0.00, 1.04) | (3.84, 9.41) |
| Xơ-đăng | 1.26 | 63.17 | 53.61 | 64.44 | 0 | 0 | 0 | 0 | 2.06 | 62.62 |
|  | (97) | (48.78, 77.57) | (40.21, 66.51) | (49.73, 79.15) |  |  |  |  | (0.00, 4.89) | (48.18, 77.05) |
| Other**^c^** | 3.85 | 8.49 | 8.08 | 8.27 | 0.67 | 0 | 0 | 0.21 | 0.34 | 8.63 |
|  | (297) | (4.79, 12.20) | (5.74, 11.27) | (4.50, 12.05) | (0.00, 1.60) |  |  | (0.00, 0.64) | (0.00, 1.00) | (4.92, 12.34) |
| **Total** | 7711 | 14.91 | 14.15 | 13.73 | 0.67 | 0.066 | 0.25 | 0.75 | 0.54 | 15.15 |
|  |  | (11.39, 18.42) | (11.03, 17.98) | (10.24, 17.22) | (0.47, 0.88) | (0.00, 0.14) | (0.13, 0.46) | (0.46, 1.24) | (0.38, 0.78) | (11.59, 18.71) |
| **Notes:**  95% CI = 95% confidence interval. Prevalence calculated amongst participants who provided a stool sample at baseline in 2019 that had qPCR performed on that sample, N=7710. **^a^** ‘Any STH’ defined as the detection of at least one following STH species the hookworms (*N. americanus*, *A. ceylanicum, A. duodenale), A. lumbricoides or T. trichiura* in stool by qPCR. This definition of ‘Any STH’ is to align with previous published estimates of STH prevalence that relied only upon microscopic techniques and thus could not effectively evaluate for the presence of *S. stercoralis*. **^b^** ‘All evaluated STH’ defined as the detection of at least one of all the evaluated STH species the hookworms (*N. americanus*, *A. ceylanicum, A. duodenale), A. lumbricoides,  T. trichiura*or *S. stercoralis* in stool by qPCR. **^c^**See Supplementary table 1 for list of ethnicities included under ‘Other’. | | | | | | | | | | |

| **Supplementary table 5.** Clustered-adjusted STH prevalence stratified by district | | | | | | | | | | |
| --- | --- | --- | --- | --- | --- | --- | --- | --- | --- | --- |
| **District** | **Study sample** | **Any STH^a^** | **Hookworm all** | ***N. americanus*** | ***A. ceylanicum*** | ***A. duodanle*** | ***A. lumbricoides*** | ***T. trichiura*** | ***S. stercoralis*** | **All evaluated STH^b^** |
|  | % | % | % | % | % | % | % | % | % |  |
|  | (n) | 95% CI | 95% CI | 95% CI | 95% CI | 95% CI | 95% CI | 95% CI | 95% CI |  |
| Buon Don | 6.65 | 18.29 | 18.19 | 17.27 | 1.23 | 0 | 0 | 0.62 | 0.82 | 18.67 |
|  | (939) | (4.67, 31.91) | (3.95, 32.42) | (3.37, 31.17) | (0.25, 2.22) |  |  | (0.00, 1.31) | (0.01, 1.63) | (4.84, 32.49) |
| Cu Kuin | 9.15 | 8.64 | 8.22 | 7.92 | 0.50 | 0 | 0 | 0.33 | 0.33 | 8.64 |
|  | (1291) | (1.50, 15.77) | (0.98, 15.45) | (0.81, 15.03) | (0.00, 1.06) |  |  | (0.00, 0.79) | (0.00, 0.79) | (1.49, 15.79) |
| Cu M’gar | 10.18 | 26.22 | 26.01 | 25.70 | 0.43 | 0.43 | 0.85 | 0.14 | 0.43 | 26.34 |
|  | (1437) | (12.28, 40.17) | (11.47, 40.55) | (11.11, 40.29) | (0.00, 0.91) | (0.00, 0.91) | (0.15, 1.56) | (0.00, 0.42) | (0.00, 0.91) | (12.35, 40.34) |
| Ea Hleo | 9.63 | 14.61 | 14.36 | 14.04 | 0.40 | 0 | 0.13 | 0.66 | 0.79 | 15.03 |
|  | (1360) | (4.97, 24.25) | (4.37, 24.35) | (4.10, 23.98) | (0.00, 0.84) |  | (0.00, 0.39) | (0.08, 1.24) | (0.16, 1.43) | (5.19, 24.88) |
| Ea Kar | 7.73 | 12.48 | 12.04 | 11.31 | 0.83 | 0 | 0 | 0.69 | 0.69 | 12.71 |
|  | (1091) | (3.95, 21.02) | (3.31, 20.76) | (2.88, 19.74) | (0.17, 1.50) |  |  | (0.09, 1.30 | (0.084, 1.31) | (4.05, 21.36) |
| Ea Sup | 6.09 | 10.24 | 9.96 | 8.58 | 1.32 | 0.26 | 0 | 0.26 | 0.53 | 10.81 |
|  | (859) | (0.00, 20.57) | (0.00, 20.60) | (0.00, 18.19) | (0.17, 2.47) | (0.00, 0,78) |  | (0.00, 0.78) | (0.00, 1.26) | (0.049, 21.58) |
| Krong Ana | 8.22 | 8.36 | 8.38 | 7.30 | 1.13 | 0 | 0 | 0.32 | 0 | 8.37 |
|  | (1160) | (1.40, 15.33) | (1.05, 15.71) | (0.63, 13.97) | (0.30, 1.96) |  |  | (0.00, 0.77) |  | (1.39, 15.35) |
| Krong Bong | 6.62 | 11.50 | 11.80 | 11.73 | 0.33 | 0 | 0 | 0.41 | 0.50 | 11.58 |
|  | (935) | (2.62, 20.39) | (2.30, 21.31) | (2.17, 21.30) | (0.00, 0.79) |  |  | (0.00, 0.98) | (0.00, 1.06) | (2.64, 20.52) |
| Krong Buk | 5.59 | 7.93 | 8.03 | 8.08 | 0.21 | 0 | 0 | 0.15 | 0 | 7.94 |
|  | (789) | (4.69, 15.40) | (0.10, 15.95) | (0.026, 16.13) | (0.00, 0.61) |  |  | (0.00, 0.44) |  | (4.62, 15.41) |
| Krong Nang | 9.02 | 8.35 | 7.91 | 7.99 | 0 | 0 | 0.30 | 1.10 | 0.15 | 8.36 |
|  | (1273) | (2.03, 14.68) | (1.54, 14.29) | (1.49, 14.49) |  |  | (0.00, 0.71) | (0.34, 1.87) | (0.00, 0.44) | (2.03, 14.70) |
| Krong Pak | 8.86 | 17.25 | 16.40 | 16.03 | 0.69 | 0.14 | 0.14 | 6.46 | 0.69 | 17.70 |
|  | (1251) | (6.48, 28.02 | (5.48, 27.32) | (5.16, 26.90) | (0.088, 1.29) | (0.00, 0.41) | (0.00, 0.41) | (3.91, 9.01) | (0.083, 1.30) | (6.72, 28.67) |
| Lak | 5.19 | 28.27 | 17.81 | 17.65 | 1.69 | 0 | 1.69 | 0.82 | 1.40 | 28.96 |
|  | (733) | (7.82, 48.72) | (1.28, 34.34) | (7.63, 37.48) | (0.35, 3.02) |  | (0.25, 3.13) | (0.10, 1.54) | (0.17, 2.64) | (8.25, 49.66) |
| M’Drak | 7.07 | 23.45 | 22.91 | 22.55 | 0.82 | 0 | 0.49 | 0.21 | 0.99 | 23.93 |
|  | (998) | (9.01, 37.90) | (8.00, 37.83) | (7.63, 37.48) | (0.10, 1.54) |  | (0.00, 1.06) | (0.00, 0.64) | (0.19, 1.78) | (9.30, 38.56) |
| **Total** | 7711 | 14.91 | 14.15 | 13.73 | 0.67 | 0.066 | 0.25 | 0.75 | 0.54 | 15.15 |
|  |  | (11.39, 18.42) | (11.03, 17.98) | (10.24, 17.22) | (0.47, 0.88) | (0.00, 0.14) | (0.13, 0.46) | (0.46, 1.24) | (0.38, 0.78) | (11.59, 18.71) |
| **Notes:**  95% CI = 95% confidence interval. Prevalence calculated amongst participants who provided a stool sample at baseline in 2019 that had qPCR performed on that sample, N=7710. **^a^** ‘Any STH’ defined as the detection of at least one following STH species the hookworms (*N. americanus*, *A. ceylanicum, A. duodenale), A. lumbricoides or T. trichiura* in stool by qPCR. This definition of ‘Any STH’ is to align with previous published estimates of STH prevalence that relied only upon microscopic techniques and thus could not effectively evaluate for the presence of *S. stercoralis*. **^b^** ‘All evaluated STH’ defined as the detection of at least one of all the evaluated STH species the hookworms (*N. americanus*, *A. ceylanicum, A. duodenale), A. lumbricoides,  T. trichiura*or *S. stercoralis* in stool by qPCR. | | | | | | | | | | |

| **Supplementary table 6.** Factors associated with all hookworm infection univariate analysis and multivariate analysis by domain | | | | | | | | | | | | |  |
| --- | --- | --- | --- | --- | --- | --- | --- | --- | --- | --- | --- | --- | --- |
|  |  | **qPCR**  **positive**  **n=1091^†^** | **qPCR**  **negative n=6619^†^** | **Univariate analysis** | | | |  | **Domain Multivariate analysis^a^** | | | | |
| ***Variables*** |  | **% (n)** | **% (n)** | **OR** | **95% CI** | | **p-value** |  | **aOR** | **95% CI** | | **p-value** | |
| ***Domain: Student demographics*** | | | | | | | | | | | | |  |
| Age (years) |  | 8.2 ± 1.36 | 7.8 ± 1.20 | 1.28 | 1.21 | 1.37 | <0.01* |  | 1.59 | 1.40 | 1.79 | <0.01^# | |
| Female |  | 43.9 (479) | 52.4 (3465) | 0.62 | 0.54 | 0.72 | <0.01* |  | 0.60 | 0.51 | 0.69 | <0.01^ | |
| Ethnicity**^b^** | Kinh | 7.5 (82) | 45.9 (3036) | Reference |  |  | * |  | Reference |  |  | ^ | |
|  | Dao | 1.8 (20) | 2.3(152) | 5.46 | 2.99 | 9.99 | <0.01 |  | 4.37 | 2.35 | 8.12 | <0.01 | |
|  | Gia-rai | 8.6 (94) | 2.3 (151) | 11.67 | 6.35 | 21.44 | <0.01 |  | 10.92 | 5.87 | 20.33 | <0.01 | |
|  | Hmông | 11.6 (126) | 1.8 (121) | 29.92 | 18.02 | 49.68 | <0.01 |  | 28.89 | 17.23 | 48.44 | <0.01 | |
|  | Mnông | 11.6 (126) | 4.6 (307) | 9.40 | 5.35 | 16.52 | <0.01 |  | 8.92 | 5.02 | 15.84 | <0.01 | |
|  | Nùng | 46 (4.2) | 6.2 (408) | 3.69 | 2.36 | 5.78 | <0.01 |  | 3.65 | 2.32 | 5.73 | <0.01 | |
|  | Tày | 2.2 (24) | 6.6 (436) | 2.11 | 1.24 | 3.58 | <0.01 |  | 2.09 | 1.23 | 3.55 | <0.01 | |
|  | Xơ-đăng | 4.8 (52) | 0.68 (45) | 68.34 | 33.19 | 140.70 | <0.01 |  | 68.14 | 32.64 | 142.23 | <0.01 | |
|  | Êđê | 45.6 (497) | 25.5 (1690) | 10.82 | 7.77 | 15.06 | <0.01 |  | 10.52 | 7.51 | 14.73 | <0.01 | |
|  | Other | 2.2 (24) | 4.1 (273) | 3.16 | 1.85 | 5.42 | <0.01 |  | 2.74 | 1.59 | 4.73 | <0.01 | |
| School graded | Grade 1 | 23.0 (251) | 26.7 (1764) | Reference |  |  | * |  | Reference |  |  | ^# | |
|  | Grade 2 | 24.7 (269) | 28.0 (1856) | 1.11 | 0.91 | 1.36 | 0.289 |  | 0.70 | 0.55 | 0.89 | <0.01 | |
|  | Grade 3 | 26.0 (284) | 23.6 (1564) | 1.33 | 1.09 | 1.62 | <0.01 |  | 0.52 | 0.38 | 0.73 | <0.01 | |
|  | Grade 4 | 26.3 (287) | 21.7 (1433) | 1.52 | 1.25 | 1.86 | <0.01 |  | 0.36 | 0.24 | 0.56 | <0.01 | |
| ***Domain: co-infection with other STH species*** | | | | | | | | | | | | |  |
| *A. lumbricoides* | | 0.5 (5) | 0.2 (14) | 1.94 | 0.62 | 6.11 | 0.257 |  | - | - | - | - | |
| *T. trichiura* |  | 1.0 (11) | 0.7 (47) | 1.03 | 0.49 | 2.15 | 0.932 |  | - | - | - | - | |
| *S. stercoralis* |  | 2.1 (23) | 0.3 (19) | 4.75 | 2.41 | 9.36 | <0.01* |  | 4.56 | 2.29 | 9.07 | <0.01^ | |
|  |  |  |  |  |  |  |  |  |  |  |  |  | |
| ***Domain: Student deworming status, personal hygiene and defecation behaviour*** | | | | | | | | | | | | |  |
| Had deworming within the last year | No | 51.8 (540) | 50.9 (3196) | Reference |  |  |  |  | - | - | - | - | |
|  | Yes | 45.2 (471) | 45.6 (2861) | 0.99 | 0.83 | 1.17 | 0.893 |  | - | - | - | - | |
|  | Don't know/refused | 3.0 (31) | 3.5 (217) | 0.77 | 0.51 | 1.18 | 0.237 |  | - | - | - | - | |
| Usual place of defecation at home**^c^** | Household toilet/latrine | 54.1 (563) | 79.8 (5004) | Reference |  |  | * |  | Reference |  |  | ^ | |
|  | Shared toilet/latrine | 1.7 (18) | 1.2 (75) | 1.11 | 0.62 | 1.99 | 0.726 |  | 1.14 | 0.63 | 2.08 | 0.665 | |
|  | On the ground outside/bushes or grass | 43.3 (450) | 18.0 (1129) | 1.66 | 1.40 | 1.97 | <0.01 |  | 1.37 | 1.11 | 1.70 | <0.01 | |
|  | Other/Don't know/refused | 0.9 (9) | 1.0 (64) | 1.17 | 0.55 | 2.48 | 0.689 |  | 1.08 | 0.49 | 2.36 | 0.856 | |
| Ever defecates outside at home**^c^** | | 66.9 (694) | 42.7 (2668) | 1.50 | 1.28 | 1.76 | <0.01* |  | 1.22 | 1.00 | 1.49 | 0.052^ | |
| Usual place of defecation at school | Toilet/latrine | 83.8 (872) | 92.4 (5783) | Reference |  |  | * |  | Reference |  |  | ^ | |
|  | On the ground outside/bushes or grass | 12.7 (132) | 4.2 (265) | 1.69 | 1.27 | 2.25 | <0.01 |  | 1.46 | 1.02 | 2.10 | 0.041 | |
|  | Doesn't defecate at school | 1.7 (18) | 2.3 (141) | 1.07 | 0.61 | 1.88 | 0.801 |  | 1.03 | 0.57 | 1.84 | 0.930 | |
|  | Don't know/refused | 1.8 (19) | 1.1 (69) | 1.21 | 0.67 | 2.22 | 0.526 |  | 1.16 | 0.59 | 2.29 | 0.667 | |
| Ever defecates outside at school**^c^** | | 24.9 (258) | 12.3 (768) | 1.39 | 1.14 | 1.69 | <0.01 |  | 1.13 | 0.87 | 1.46 | 0.356 | |
| Washes hands after defecating | Always | 46.7 (487) | 62.1 (3895) | Reference |  |  | * |  | Reference |  |  | ^ | |
|  | Sometimes | 43.1 (450) | 32.5 (2039) | 1.23 | 1.05 | 1.45 | 0.010 |  | 1.39 | 0.99 | 1.95 | 0.055 | |
|  | Never | 10.1 (105) | 5.3 (330) | 1.48 | 1.12 | 1.96 | <0.01 |  | 1.24 | 1.02 | 1.51 | 0.031 | |
|  | Don’t know/refused | 0.1 (1) | 0.1 (7) | 0.39 | 0.04 | 3.69 | 0.409 |  | 0.22 | 0.02 | 2.33 | 0.210 | |
| Washes hands before eating | Always | 39.4 (410) | 54.0 (3380) | Reference |  |  | * |  | Reference |  |  |  | |
|  | Sometimes | 52.6 (548) | 40.3 (2522) | 1.19 | 1.01 | 1.39 | 0.016 |  | 1.02 | 0.72 | 1.46 | 0.901 | |
|  | Never | 7.9 (82) | 5.7 (354) | 1.44 | 1.07 | 1.93 | 0.035 |  | 1.07 | 0.87 | 1.30 | 0.531 | |
|  | Don’t know/refused | 0.2 (2) | 0.1 (8) | 1.63 | 0.30 | 8.95 | 0.574 |  | 2.02 | 0.33 | 12.57 | 0.449 | |
| Wears shoes outside | Always | 65.3 (680) | 73.5 (4613) | Reference |  |  | * |  | Reference |  |  |  | |
|  | Sometimes | 32.4 (337) | 24.7 (1548) | 1.20 | 1.01 | 1.43 | 0.038 |  | 1.41 | 0.82 | 2.43 | 0.219 | |
|  | Never | 2.2 (23) | 1.6 (103) | 1.43 | 0.86 | 2.39 | 0.167 |  | 1.17 | 0.96 | 1.42 | 0.114 | |
|  | Don’t know/refused | 0.1 (1) | 0.2 (10) | 0.90 | 0.11 | 7.44 | 0.924 |  | 1.04 | 0.11 | 10.15 | 0.976 | |
| Wears shoes defecating | Always | 73.0 (760) | 65.9 (4137) | Reference |  |  | * |  | Reference |  |  | ^ | |
|  | Sometimes | 16.7 (174) | 18.7 (1175) | 0.90 | 0.73 | 1.10 | 0.307 |  | 0.74 | 0.56 | 0.96 | 0.024 | |
|  | Never | 9.6 (100) | 14.6 (917) | 0.79 | 0.61 | 1.00 | 0.054 |  | 0.79 | 0.62 | 0.99 | 0.039 | |
|  | Don’t know/refused | 0.7 (7) | 0.8 (48) | 0.90 | 0.39 | 2.11 | 0.812 |  | 0.72 | 0.29 | 1.79 | 0.477 | |
| ***Domain: Household WASH factors*** | | | | | | | | | | | | |  |
| Household water supply**^d^** | Improved | 75.0 (784) | 86.8 (5450) | Reference |  |  | * |  | Reference |  |  | ^ | |
|  | Unimproved | 23.7 (247) | 11.3 (708) | 1.52 | 1.26 | 1.84 | <0.01 |  | 1.53 | 1.25 | 1.87 | <0.01 | |
|  | Don't know/refused | 1.3 (13) | 1.9 (120) | 0.98 | 0.53 | 1.82 | 0.950 |  | 0.94 | 0.49 | 1.80 | 0.855 | |
| Household toilet/latrine | No | 47.7 (496) | 20.8 (1305) | Reference |  |  | * |  | Reference |  |  | ^ | |
|  | Yes, but does not flush | 18.0 (187) | 14.2 (889) | 0.81 | 0.65 | 1.00 | 0.051 |  | 0.82 | 0.65 | 1.03 | 0.082 | |
|  | Yes, flushes with water | 34.3 (357) | 65.0 (4069) | 0.48 | 0.40 | 0.58 | <0.01 |  | 0.49 | 0.40 | 0.60 | <0.01 | |
| Handwashing station | No | 17.3 (178) | 7.4 (460) | Reference |  |  | * |  | Reference |  |  | ^ | |
|  | Yes, but no soap available | 35.6 (367) | 23.1 (1440) | 0.85 | 0.65 | 1.09 | 0.201 |  | 0.82 | 0.63 | 1.08 | 0.158 | |
|  | Yes, soap available | 47.1 (486) | 69.5 (4330) | 0.57 | 0.44 | 0.73 | <0.01 |  | 0.67 | 0.52 | 0.88 | <0.01 | |
| ***Domain: Household socioeconomic factors*** | | | | | | | | | | | | |  |
| Mother's highest level of education | Never attended school | 19.0 (198) | 6.2 (390) | Reference |  |  | * |  | Reference |  |  | ^ | |
|  | Completed or attended primary school | 43.0 (449) | 27.2 (1712) | 0.69 | 0.55 | 0.86 | <0.01 |  | 0.73 | 0.58 | 0.92 | <0.01 | |
|  | Completed or attended secondary school | 32.6 (340) | 52.6 (3306) | 0.40 | 0.32 | 0.50 | <0.01 |  | 0.46 | 0.36 | 0.58 | <0.01 | |
|  | Completed or attended tertiary education | 2.6 (27) | 10.2 (640) | 0.19 | 0.12 | 0.30 | <0.01 |  | 0.28 | 0.18 | 0.46 | <0.01 | |
|  | Don't know/refused | 2.9 (30) | 3.9 (243) | 0.41 | 0.26 | 0.65 | <0.01 |  | 0.43 | 0.27 | 0.67 | <0.01 | |
| Occupation main household income earner**^h^** | Farmer/animal keeper | 97.0 (1012) | 91.6 (5766) | Reference |  |  | * |  | Reference |  |  | ^ | |
|  | Fisherman/market seller | 0.7 (7) | 2.2 (135) | 0.55 | 0.25 | 1.22 | 0.141 |  | 0.66 | 0.29 | 1.48 | 0.309 | |
|  | Administration/healthcare worker/Other | 0.8 (8) | 4.7 (295) | 0.26 | 0.12 | 0.53 | <0.01 |  | 0.48 | 0.22 | 1.03 | 0.059 | |
|  | Unemployed | 0.6 (6) | 0.5 (30) | 1.45 | 0.57 | 3.66 | 0.431 |  | 1.43 | 0.55 | 3.73 | 0.467 | |
|  | Don't know/refused | 1.0 (10) | 1.1 (66) | 1.07 | 0.52 | 2.24 | 0.847 |  | 1.17 | 0.54 | 2.50 | 0.694 | |
| Household income | Less than VND 20,000,000 | 55.6 (580) | 43.2 (2713) | Reference |  |  | * |  | Reference |  |  | ^ | |
|  | VND 20,000,000 – 50,000,000 | 15.3 (160) | 28.0 (1760) | 0.64 | 0.52 | 0.79 | <0.01 |  | 0.73 | 0.59 | 0.91 | <0.01 | |
|  | VND 50,000,000 – 200,000,000 | 3.3 (34) | 7.7 (483) | 0.49 | 0.33 | 0.73 | <0.01 |  | 0.66 | 0.44 | 1.00 | 0.050 | |
|  | More than VND 200,000,000 | 0.3 (3) | 0.7 (44) | 0.77 | 0.22 | 2.68 | 0.685 |  | 0.91 | 0.26 | 3.25 | 0.889 | |
|  | Don't know/refused | 25.6 (267) | 20.5 (1288) | 1.01 | 0.85 | 1.21 | 0.889 |  | 0.94 | 0.78 | 1.13 | 0.503 | |
| ***Domain: School location and WASH factors*** | | | | | | | | | | | | |  |
| Location | Rural | 43.2 (471) | 58.8 (3894) | Reference |  |  | * |  | Reference |  |  | ^ | |
|  | Remote | 34.5 (376) | 32.3 (2139) | 1.76 | 0.92 | 3.37 | 0.088 |  | 1.60 | 0.86 | 2.97 | 0.136 | |
|  | Very remote | 22.4 (244) | 8.9 (586) | 4.55 | 1.74 | 11.87 | <0.01 |  | 3.21 | 1.19 | 8.68 | 0.022 | |
| School water source**^d^** | Improved | 34.8 (380) | 43.8 (22902) | Reference |  |  | * |  | Reference |  |  | ^ | |
|  | Unimproved | 22.4 (244) | 13.0 (863) | 2.80 | 1.10 | 7.10 | 0.030 |  | 1.90 | 0.80 | 4.53 | 0.148 | |
|  | No water | 25.0 (273) | 27.7 (1834) | 1.15 | 0.54 | 2.44 | 0.719 |  | 0.71 | 0.35 | 1.42 | 0.329 | |
|  | Don't know/refused | 17.8 (194) | 15.4 (1020) | 1.94 | 0.79 | 4.76 | 0.150 |  | 1.59 | 0.72 | 3.53 | 0.251 | |
| School toilets available to students | | 94.1 (1027) | 97.4 (6447) | 0.31 | 0.05 | 1.81 | 0.193* |  | 0.37 | 0.06 | 2.24 | 0.280 | |
| School sanitation**^e^** | Improved | 95.2 (1039) | 95.2 (6304) | Reference |  |  |  |  | - | - | - | - | |
|  | Unimproved | 4.8 (52) | 4.8 (315) | 0.52 | 0.11 | 2.49 | 0.413 |  | - | - | - | - | |
| School handwahsing station**^l^** | No | 9.6 (103) | 11.8 (753) | Reference |  |  | * |  | Reference |  |  | ^ | |
|  | Yes, but no soap | 70.7 (763) | 53.8 (3441) | 1.97 | 0.74 | 5.22 | 0.173 |  | 3.72 | 1.25 | 11.06 | 0.018 | |
|  | Yes, soap available | 19.7 (213) | 34.4 (2197) | 0.58 | 0.20 | 1.65 | 0.307 |  | 1.17 | 0.37 | 3.67 | 0.785 | |
| Latrine/toilet able to be locked | | 11.5 (125) | 5.4 (354) | 2.34 | 0.64 | 8.46 | 0.197* |  | 1.38 | 0.41 | 4.71 | 0.604 | |
| Latrine/toilet observed to be clean | | 42.9 (468) | 61.7 (4083) | 0.57 | 0.30 | 1.07 | 0.082* |  | 0.98 | 0.53 | 1.82 | 0.942 | |
| Latrine/toilet has water for cleaning self | | 48.9 (533) | 48.5 (3208) | 1.09 | 0.57 | 2.06 | 0.797 |  | - | - | - | - | |
| Latrine/toilet observed to be dirty | | 45.0 (491) | 29.5 (1952) | 1.53 | 0.78 | 3.01 | 0.220 |  | - | - | - | - | |
| Latrine/toilet observed to have flies | | 21.9 (238) | 14.7 (972) | 1.26 | 0.52 | 3.04 | 0.612 |  | - | - | - | - | |
| Number of school toilets | | 5.2 ± 3.56 | 5.2 ± 3.86 | 1.03 | 0.95 | 1.12 | 0.514 |  | - | - | - | - | |
|  |  |  |  |  |  |  |  |  |  |  |  |  | |
| **Notes:** WASH = water, sanitation and hygiene. All hookworm = infection with at least one of *N. americanus, A, duodanale or A. ceylanicum*. aOR = adjusted odds ratio, OR = odds ratio, 95% CI = 95% confidence interval, STH = soil-transmitted helminths. Reference = reference category used for regression for categorical variables. **^†^**Numbers and proportion of qPCR positive and negative participants for each variable, except for continuous variables, reported as mean ± standard deviation. **^a^**Domain multivariable models conducted using covariates with p<0.2 on the Wald test from univariate analysis indicated by *. All domain level multivariate models included the covariates of sex and age. ^Denotes varibales from domain multivariable models with p<0.1 on Wald test and included in backwards stepwise elimination procedure to produce final multivariable models. **^b^**See Supplementary table 1 for list ethnicities grouped in 'Other'. **^c^**Captures students who report ever defecating outside at home or school. **^d,e^**Responses for variables collapsed to align (or approximate where not enough information available) with WHO and United Nations International Children’s Emergency Fund (UNICEF) Joint Monitoring Programme (JMP) for Water Supply and Sanitation definitions. **^d^**Improved = piped water, protected well, rainwater or public tap. Unimproved = unprotected well or surface water. **^e^**Improved = flush toilet or pit latrine with a slab. Unimproved = pit latrine without a slab. (-)Indicates categories not included in regression at that step. #Age and grade level found to be only variables collinear (VIF>5) in initial final multivariable model. Initial models tested with age and grade independently and the model with age had a lower AIC than that with grade. Grade was dropped from the analysis at this stage prior to stepwise regression. | | | | | | | | | | | | |  |

| **Supplementary table 7.** Factors associated with *Necator americanus*, univariate and domain level multivariate analysis | | | | | | | | | | | | | |
| --- | --- | --- | --- | --- | --- | --- | --- | --- | --- | --- | --- | --- | --- |
|  |  | **qPCR positive n=1058^†^** | **qPCR negative n=6652^†^** | **Univariate analysis** | | | |  | **Domain Multivariate analysis^a^** | | | | |
| ***Variables*** |  | **% (n)** | **% (n)** | **OR** | **95% CI** | | **p-value** |  | **aOR** | | **95% CI** | | **p-value** |
| ***Domain: demographics*** | | | | | | | | | | | | | |
| Age (years) |  | 8.2 ± 1.36 | 7.8 ± 1.2 | 1.28 | 1.21 | 1.35 | <0.01* |  | 1.59 | | 1.40 | 1.79 | <0.01^# |
| Female |  | 43.6 (461) | 52.4 (3483) | 0.61 | 0.53 | 0.71 | <0.01* |  | 0.58 | | 0.50 | 0.68 | <0.01^ |
| Ethnicity**^b^** | Kinh | 6.9 (73) | 45.8 (3045) | Reference |  |  | * |  | Reference | |  |  | ^ |
|  | Dao | 1.8 (19) | 2.3 (153) | 6.17 | 3.32 | 11.49 | <0.01 |  | 4.93 | | 2.60 | 9.33 | <0.01 |
|  | Gia-rai | 8.9 (94) | 2.3 (151) | 13.07 | 7.03 | 24.28 | <0.01 |  | 12.35 | | 6.55 | 23.26 | <0.01 |
|  | Hmông | 11.7 (124) | 1.9 (123) | 32.99 | 19.64 | 55.42 | <0.01 |  | 31.91 | | 18.80 | 54.18 | <0.01 |
|  | Mnông | 11.7 (124) | 4.7 (309) | 9.84 | 5.51 | 17.58 | <0.01 |  | 9.31 | | 5.15 | 16.82 | <0.01 |
|  | Nùng | 4.2 (44) | 6.2 (410) | 4.06 | 2.56 | 6.44 | <0.01 |  | 4.01 | | 2.52 | 6.39 | <0.01 |
|  | Tày | 2.1 (22) | 6.6 (438) | 2.18 | 1.26 | 3.79 | <0.01 |  | 2.16 | | 1.24 | 3.75 | <0.01 |
|  | Xơ-đăng | 4.9 (52) | 0.7 (45) | 84.59 | 40.21 | 177.95 | <0.01 |  | 85.21 | | 39.92 | 181.91 | <0.01 |
|  | Êđê | 45.7 (483) | 25.6 (1704) | 11.73 | 8.31 | 16.56 | <0.01 |  | 11.45 | | 8.06 | 16.26 | <0.01 |
|  | Other | 2.2 (23) | 4.1 (274) | 3.46 | 1.99 | 6.00 | <0.01 |  | 2.98 | | 1.70 | 5.22 | <0.01 |
| School graded | Grade 1 | 22.5 (238) | 26.7 (1777) | Reference |  |  | * |  | Reference | |  |  | ^# |
|  | Grade 2 | 24.7 (261) | 28.0 (1864) | 1.15 | 0.94 | 1.41 | 0.181 |  | 0.72 | | 0.56 | 0.93 | 0.010 |
|  | Grade 3 | 26.4 (279) | 23.6 (1569) | 1.39 | 1.13 | 1.70 | <0.01 |  | 0.55 | | 0.39 | 0.77 | <0.01 |
|  | Grade 4 | 26.5 (280) | 21.7 (1440) | 1.58 | 1.29 | 1.94 | <0.01 |  | 0.38 | | 0.25 | 0.59 | <0.01 |
| ***Domain: co-infection with other STH species*** | | | | | | | | | | | | | |
| *A. lumbricoides* | | 0.5 (5) | 0.2 (14) | 2.02 | 0.64 | 6.36 | 0.23 |  | - | | - | - | - |
| *T. trichiura* |  | 1.0 (10) | 0.7 (48) | 0.92 | 0.43 | 1.97 | 0.837 |  | - | | - | - | - |
| *A. ceylanicum* | | 2.2 (23) | 0.4 (29) | 5.05 | 2.75 | 9.28 | <0.01* |  | 4.80 | | 2.57 | 9.00 | <0.01^ |
| *S. stercoralis* | | 2.2 (23) | 0.3 (19) | 4.93 | 2.49 | 9.75 | <0.01* |  | 4.54 | | 2.25 | 9.14 | <0.01^ |
| ***Domain: Health, personal hygiene and defecation behaviour*** | | | | | | | | | | | | | |
| Had deworming within the last year | No | 51.5 (520) | 51.0 (3126) | Reference |  |  |  |  | - | | - | - | - |
|  | Yes | 45.5 (459) | 45.6 (2873) | 1.01 | 0.85 | 1.20 | 0.899 |  | - | | - | - | - |
|  | Don't know/refused | 3.1 (31) | 3.4 (217) | 0.81 | 0.53 | 1.24 | 0.336 |  | | - | - | - | - |
| Usual place of defecation at home**^c^** | Household toilet/latrine | 53.2 (536) | 79.8 (5031) | Reference |  |  | * |  | Reference | |  |  | ^ |
|  | Shared toilet/latrine | 1.8 (18) | 1.2 (75) | 1.16 | 0.65 | 2.09 | 0.619 |  | 1.20 | | 0.65 | 2.19 | 0.558 |
|  | On the ground outside/bushes or grass | 44.2 (445) | 18.0 (1134) | 1.71 | 1.44 | 2.03 | <0.01 |  | 1.40 | | 1.13 | 1.74 | 0.002 |
|  | Other/Don't know/refused | 0.9 (9) | 1.0 (64) | 1.24 | 0.58 | 2.65 | 0.571 |  | 1.13 | | 0.51 | 2.50 | 0.756 |
| Ever defecates outside at home**^c^** |  | 67.7 (680) | 42.7 (2682) | 1.54 | 1.31 | 1.81 | <0.01* |  | 1.23 | | 1.00 | 1.51 | 0.047^ |
| Usual place of defecation at school | Toilet/latrine | 83.6 (843) | 92.4 (5812) | Reference |  |  | * |  | Reference | |  |  |  |
|  | On the ground outside/bushes or grass | 13.0 (131) | 4.2 (266) | 1.74 | 1.31 | 2.33 | <0.01 |  | 1.49 | | 1.03 | 2.15 | 0.034 |
|  | Doesn't defecate at school | 1.8 (18) | 2.2 (141) | 1.12 | 0.64 | 1.96 | 0.693 |  | 1.06 | | 0.59 | 1.91 | 0.844 |
|  | Don't know/refused | 1.7 (17) | 1.1 (71) | 1.07 | 0.57 | 2.02 | 0.823 |  | 0.96 | | 0.47 | 1.97 | 0.916 |
| Ever defecates outside at school**^d^** | | 25.3 (254) | 12.3 (772) | 1.42 | 1.16 | 1.73 | <0.01* |  | 1.14 | | 0.88 | 1.48 | 0.322 |
| Washes hands after defecating | Always | 46.3 (468) | 62.1 (3914) | Reference |  |  | * |  | Reference | |  |  | ^ |
|  | Sometimes | 43.3 (438) | 32.5 (2051) | 1.52 | 1.14 | 2.01 | <0.01 |  | 1.38 | | 0.98 | 1.95 | 0.062 |
|  | Never | 10.3 (104) | 5.3 (331) | 1.23 | 1.05 | 1.45 | 0.012 |  | 1.24 | | 1.01 | 1.52 | 0.036 |
|  | Don’t know/refused | 0.1 (1) | 0.1 (7) | 0.39 | 0.04 | 3.74 | 0.415 |  | 0.22 | | 0.02 | 2.36 | 0.213 |
| Washes hands before eating | Always | 38.9 (393) | 54.0 (3397) | Reference |  |  | * |  | Reference | |  |  |  |
|  | Sometimes | 52.8 (533) | 40.3 (2537) | 1.52 | 1.13 | 2.04 | <0.01 |  | 1.07 | | 0.75 | 1.53 | 0.708 |
|  | Never | 8.1 (82) | 5.6 (354) | 1.19 | 1.01 | 1.40 | 0.036 |  | 1.07 | | 0.87 | 1.31 | 0.511 |
|  | Don’t know/refused | 0.2 (2) | 0.1 (8) | 1.75 | 0.32 | 9.66 | 0.524 |  | 2.14 | | 0.34 | 13.65 | 0.421 |
| Wears shoes outside | Always | 65.2 (658) | 73.5 (4635) | Reference |  |  | * |  | Reference | |  |  |  |
|  | Sometimes | 32.4 (327) | 24.7 (1558) | 1.54 | 0.92 | 2.58 | 0.098 |  | 1.50 | | 0.86 | 2.60 | 0.152 |
|  | Never | 2.3 (23) | 1.6 (103) | 1.20 | 1.01 | 1.43 | 0.041 |  | 1.17 | | 0.96 | 1.43 | 0.123 |
|  | Don’t know/refused | 0.1 (1) | 0.2 (10) | 0.99 | 0.12 | 8.18 | 0.992 |  | 1.07 | | 0.11 | 10.71 | 0.955 |
| Wears shoes defecating | Always | 73.1 (738) | 65.9 (4159) | reference |  |  | * |  | Reference | |  |  | ^ |
|  | Sometimes | 16.7 (168) | 18.7 (1181) | 0.80 | 0.62 | 1.03 | 0.078 |  | 0.74 | | 0.57 | 0.97 | 0.032 |
|  | Never | 9.5 (96) | 14.6 (921) | 0.90 | 0.73 | 1.10 | 0.3 |  | 0.78 | | 0.62 | 0.98 | 0.036 |
|  | Don’t know/refused | 0.7 (7) | 0.8 (48) | 0.96 | 0.41 | 2.26 | 0.93 |  | 0.76 | | 0.30 | 1.90 | 0.556 |
| ***Domain: Household WASH factors*** | | | | | | | | | | | | | |
| Household water supply**^d^** | Improved | 75.1 (761) | 86.8 (5473) | Reference |  |  | * |  | Reference | |  |  | ^ |
|  | Unimproved | 24.0 (239) | 11.4 (716) | 1.49 | 1.23 | 1.81 | <0.01 |  | 1.50 | | 1.22 | 1.83 | <0.01 |
|  | Don't know/refused | 1.3 (13) | 1.9 (120) | 1.03 | 0.55 | 1.92 | 0.927 |  | 0.99 | | 0.51 | 1.90 | 0.971 |
| Household toilet/latrine | No | 48.6 (490) | 20.8 (1311) | Reference |  |  | * |  | Reference | |  |  | ^ |
|  | Yes, but does not flush | 17.6 (177) | 14.3 (899) | 0.77 | 0.62 | 0.96 | 0.021 |  | 0.78 | | 0.62 | 0.99 | 0.04 |
|  | Yes, flushes with water | 33.8 (341) | 64.9 (4085) | 0.47 | 0.39 | 0.57 | <0.01 |  | 0.47 | | 0.39 | 0.58 | <0.01 |
| Handwashing station | No | 17.7 (177) | 7.4 (461) | Reference |  |  | * |  | Reference | |  |  | ^ |
|  | Yes, but no soap available | 35.5 (355) | 23.2 (1452) | 0.85 | 0.65 | 1.09 | 0.201 |  | 0.79 | | 0.61 | 1.04 | 0.095 |
|  | Yes, soap available | 46.8 (468) | 69.5 (4348) | 0.57 | 0.44 | 0.73 | <0.01 |  | 0.66 | | 0.51 | 0.86 | 0.003 |
| ***Domain: Household socioeconomic factors*** | | | | | | | | | | | | | |
| Mother's highest level of education | Never attended school | 19.4 (196) | 6.2 (392) | Reference |  |  | * |  | Reference | |  |  | ^ |
|  | Completed or attended primary school | 43.2 (438) | 27.3 (1723) | 0.68 | 0.55 | 0.85 | <0.01 |  | 0.73 | | 0.91 | 0.91 | <0.01 |
|  | Completed or attended secondary school | 31.8 (322) | 52.6 (3324) | 0.39 | 0.31 | 0.49 | <0.01 |  | 0.44 | | 0.56 | 0.56 | <0.01 |
|  | Completed or attended tertiary education | 2.7 (27) | 10.1 (640) | 0.20 | 0.13 | 0.31 | <0.01 |  | 0.31 | | 0.50 | 0.50 | <0.01 |
|  | Don't know/refused | 3.0 (30) | 3.8 (243) | 0.42 | 0.27 | 0.66 | <0.01 |  | 0.44 | | 0.69 | 0.69 | <0.01 |
| Occupation main household income earner | Farmer/animal keeper | 97.3 (985) | 91.6 (5793) | Reference |  |  | * |  | Reference | |  |  | ^ |
|  | Fisherman/market seller | 0.6 (6) | 2.2 (136) | 0.49 | 0.21 | 1.15 | 0.102 |  | 0.57 | | 0.24 | 1.36 | 0.208 |
|  | Administration/healthcare worker/Other | 0.6 (6) | 4.7 (297) | 0.20 | 0.09 | 0.45 | <0.01 |  | 0.35 | | 0.15 | 0.84 | 0.018 |
|  | Unemployed | 0.6 (6) | 0.5 (30) | 1.52 | 0.60 | 3.85 | 0.372 |  | 1.48 | | 0.57 | 3.89 | 0.422 |
|  | Don't know/refused | 0.9 (9) | 1.1 (67) | 0.97 | 0.45 | 2.08 | 0.934 |  | 1.02 | | 0.46 | 2.26 | 0.96 |
| Household income | Less than VND 20,000,000 | 55.4 (561) | 43.2 (2732) | Reference |  |  | * |  | Reference | |  |  | ^ |
|  | VND 20,000,000 – 50,000,000 | 15.2 (154) | 28.0 (1766) | 0.65 | 0.52 | 0.80 | <0.01 |  | 0.74 | | 0.59 | 0.92 | <0.01 |
|  | VND 50,000,000 – 200,000,000 | 3.3 (33) | 7.7 (484) | 0.50 | 0.34 | 0.75 | <0.01 |  | 0.68 | | 0.44 | 1.03 | 0.069 |
|  | More than VND 200,000,000 | 0.3 (3) | 0.7 (44) | 0.82 | 0.24 | 2.85 | 0.756 |  | 0.97 | | 0.27 | 3.46 | 0.96 |
|  | Don't know/refused | 25.9 (262) | 20.5 (1293) | 1.04 | 0.87 | 1.25 | 0.647 |  | 0.97 | | 0.80 | 1.16 | 0.712 |
| ***Domain: School WASH factors*** | | | | | | | | | | | | | |
| Location | Rural | 42.6 (451) | 58.8 (3914) | Reference |  |  | * |  | Reference | |  |  | ^ |
|  | Remote | 34.5 (365) | 32.3 (2150) | 1.81 | 0.94 | 3.51 | 0.076 |  | 1.66 | | 0.88 | 3.11 | 0.117 |
|  | Very remote | 22.9 (242) | 8.8 (588) | 4.84 | 1.83 | 12.79 | 0.001 |  | 3.42 | | 1.24 | 9.41 | 0.017 |
| School water source**^d^** | Improved | 34.8 (368) | 43.8 (2914) | Reference |  |  | * |  | Reference | |  |  | ^ |
|  | Unimproved | 22.6 (239) | 13.1 (868) | 2.84 | 1.10 | 7.34 | 0.031 |  | 1.88 | | 0.78 | 4.55 | 0.162 |
|  | No water | 25.1 (265) | 27.7 (1842 | 1.18 | 0.55 | 2.55 | 0.67 |  | 0.71 | | 0.35 | 1.45 | 0.348 |
|  | Don't know/refused | 17.6 (186) | 15.5 (1028) | 1.97 | 0.79 | 4.93 | 0.148 |  | 1.61 | | 0.72 | 3.61 | 0.249 |
| School toilets available to students | | 94.1 (995) | 97.4 (6479) | 0.30 | 0.05 | 1.83 | 0.193* |  | 0.34 | | 0.05 | 2.16 | 0.255 |
| School sanitation**^e^** | Improved | 95.2 (1007) | 95.3 (6336) | Reference |  |  |  |  | - | | - | - | - |
|  | Unimproved | 4.8 (51) | 4.8 (316) | 0.46 | 0.09 | 2.34 | 0.351 |  | - | | - | - | - |
| School handwahsing station | No | 9.5 (99) | 11.8 (757) | Reference |  |  | * |  | Reference | |  |  | ^ |
|  | Yes, but no soap | 70.8 (741) | 53.9 (3463) | 2.07 | 0.76 | 5.60 | 0.153 |  | 3.10 | | 1.17 | 8.23 | 0.023 |
|  | Yes, soap available | 19.8 (207) | 34.3 (2203) | 0.61 | 0.21 | 1.78 | 0.366 |  | 0.98 | | 0.34 | 2.84 | 0.972 |
| Latrine/toilet able to be locked | | 11.6 (123) | 5.4 (356) | 2.42 | 0.65 | 8.94 | 0.187* |  | 1.44 | | 0.42 | 5.01 | 0.564 |
| Latrine/toilet observed to be clean | | 42.3 (448) | 61.7 (4103) | 0.57 | 0.30 | 1.09 | 0.091* |  | 1.01 | | 0.53 | 1.89 | 0.987 |
| Latrine/toilet has water for cleaning self | | 48.8 (516) | 48.5 (3225) | 1.10 | 0.57 | 2.10 | 0.784 |  | - | | - | - | - |
| Latrine/toilet observed to be dirty | | 45.5 (481) | 29.5 (1962) | 1.53 | 0.77 | 3.04 | 0.23 |  | - | | - | - | - |
| Latrine/toilet observed to have flies | | 22.1 (234) | 14.7 (976) | 1.25 | 0.51 | 3.08 | 0.624 |  | - | | - | - | - |
| Number of school toilets | | 5.2 ± 3.48 | 5.2 ± 3.87 | 1.02 | 0.94 | 1.11 | 0.592 |  | - | | - | - | - |
| **Notes:** WASH = water, sanitation and hygiene. All hookworm = infection with at least one of *N. americanus, A, duodanale or A. ceylanicum*. aOR = adjusted odds ratio, OR = odds ratio, 95% CI = 95% confidence interval, STH = soil-transmitted helminths. Reference = reference category used for regression for categorical variables. **^†^**Numbers and proportion of qPCR positive and negative participants for each variable, except for continuous variables, reported as mean ± standard deviation. **^a^**Domain multivariable models conducted using covariates with p<0.2 on the Wald test from univariate analysis indicated by *. All domain level multivariate models included the covariates of sex and age. ^Denotes varibales from domain multivariable models with p<0.1 on Wald test and included in backwards stepwise elimination procedure to produce final multivariable models. **^b^**See Supplementary table 1 for list ethnicities grouped in 'Other'. **^c^**Captures students who report ever defecating outside at home or school. **^d,e^**Responses for variables collapsed to align (or approximate where not enough information available) with WHO and United Nations International Children’s Emergency Fund (UNICEF) Joint Monitoring Programme (JMP) for Water Supply and Sanitation definitions. **^d^**Improved = piped water, protected well, rainwater or public tap. Unimproved = unprotected well or surface water. **^e^**Improved = flush toilet or pit latrine with a slab. Unimproved = pit latrine without a slab. (-)Indicates categories not included in regression at that step. #Age and grade level found to be only variables collinear (VIF>5) in initial final multivariable model. Initial models tested with age and grade independently and the model with age had a lower AIC than that with grade. Grade was dropped from the analysis at this stage prior to stepwise regression. | | | | | | | | | | | | | |

| **Supplementary table 8.** Factors associated with moderate-to-heavy intensity *Necator Americanus* infection: univariate analysis and multivariate analysis by domain | | | | | | | | | | | | |
| --- | --- | --- | --- | --- | --- | --- | --- | --- | --- | --- | --- | --- |
|  |  | **Moderate-to-heavy intensity infection  n=251^†^** | **Light intensity infection**  **n=807^†^** | **Univariate analysis** | | | |  | **Domain Multivariate analysis^a^** | | | |
| ***Variable*** |  | **% (n)** | **% (n)** | **OR** | **95% CI** | | **p-value** |  | **aOR** | **95% CI** | | **p-value** |
| ***Domain: demographics*** | | | | | | | | | | | | |
| Age (years) |  | 8.4 ± 1.33 | 8.2 ± 1.37 | 1.13 | 1.01 | 1.26 | 0.032 |  | 1.21 | 1.00 | 1.48 | 0.055 |
| Female |  | 46.6 (117) | 42.6 (344) | 1.13 | 0.84 | 1.53 | 0.410 |  | 1.13 | 0.83 | 1.53 | 0.443 |
| Ethnicity**^b^** | Kinh | 3.2 (8) | 8.1 (65) | Reference |  |  | * |  | Reference |  |  | ^ |
|  | Dao | 0.4 (1) | 2.2 (18) | 0.30 | 0.03 | 2.86 | 0.293 |  | 0.27 | 0.03 | 2.62 | 0.257 |
|  | Gia-rai | 11.2 (28) | 8.2 (66) | 4.21 | 1.30 | 13.65 | 0.016 |  | 4.25 | 1.29 | 14.02 | 0.017 |
|  | Hmông | 13.6 (34) | 11.2 (90) | 3.46 | 1.17 | 10.24 | 0.025 |  | 3.26 | 1.10 | 9.72 | 0.034 |
|  | Mnông | 12.8 (32) | 11.4 (92) | 2.86 | 0.93 | 8.81 | <0.01 |  | 2.80 | 0.90 | 8.64 | 0.074 |
|  | Nùng | 2.8 (7) | 4.6 (37) | 1.52 | 0.46 | 5.03 | 0.490 |  | 1.55 | 0.47 | 5.16 | 0.474 |
|  | Tày | 1.6 (4) | 2.2 (18) | 2.03 | 0.49 | 8.46 | 0.331 |  | 2.05 | 0.49 | 8.58 | 0.328 |
|  | Xơ-đăng | 6.8 (17) | 4.3 (35) | 4.34 | 1.22 | 15.44 | 0.023 |  | 4.05 | 1.13 | 14.51 | 0.032 |
|  | Êđê | 45.4 (114) | 45.7 (369) | 2.84 | 1.19 | 6.77 | 0.018 |  | 2.78 | 1.16 | 6.67 | 0.022 |
|  | Other | 2.4 (6) | 2.1 (17) | 2.77 | 0.75 | 10.25 | 0.127 |  | 2.44 | 0.65 | 9.20 | 0.187 |
| School grade**^b^** | Grade 1 | 19.5 (49) | 23.4 (189) | Reference |  |  | * |  | Reference |  |  |  |
|  | Grade 2 | 27.1 (68) | 23.9 (193) | 1.42 | 0.92 | 2.19 | 0.118 |  | 1.17 | 0.70 | 1.95 | 0.540 |
|  | Grade 3 | 24.3 (61) | 27.0 (218) | 1.08 | 0.69 | 1.68 | 0.735 |  | 0.71 | 0.38 | 1.35 | 0.299 |
|  | Grade 4 | 29.1 (73) | 25.7 (207) | 1.44 | 0.93 | 2.24 | 0.100 |  | 0.80 | 0.37 | 1.76 | 0.584 |
| ***Domain: co-infection with other STH species*** | | | | | | | | | | | | |
| *A. lumbricoides* |  | 0.4 (1) | 0.5 (4) | 1.36 | 0.14 | 13.65 | 0.793 |  | ***-*** | ***-*** | ***-*** | ***-*** |
| *T. trichiura* |  | 1.6 (4) | 0.7 (6) | 2.00 | 0.52 | 7.65 | 0.313 |  | ***-*** | ***-*** | ***-*** | ***-*** |
| *S. stercoralis* |  | 4.4 (11) | 1.5 (12) | 2.50 | 1.05 | 5.98 | 0.039* |  | 2.39 | 1.00 | 5.73 | 0.050 |
| *A. ceylanicum* |  | 2.4 (6) | 2.1 (17) | 1.43 | 0.53 | 3.90 | 0.480 |  |  |  |  |  |
| ***Domain: Deworming, personal hygiene and defecation behaviour*** | | | | | | | | | | | | |
| Had deworming within the last year | No | 50.6 (123) | 51.8 (397) | Reference |  |  |  |  | ***-*** | ***-*** | ***-*** | ***-*** |
|  | Yes | 45.7 (111) | 45.4 (348) | 0.97 | 0.70 | 1.36 | 0.870 |  | ***-*** | ***-*** | ***-*** | ***-*** |
|  | Don't know/refused | 3.7 (9) | 2.9 (22) | 1.26 | 0.53 | 3.00 | 0.599 |  | ***-*** | ***-*** | ***-*** | ***-*** |
| Usual place of defecation at home**^d^** | Household toilet/latrine | 53.3 (129) | 53.1 (407) | Reference |  |  |  |  | ***-*** | ***-*** | ***-*** | ***-*** |
|  | Shared toilet/latrine | 2.5 (6) | 1.6 (12) | 1.26 | 0.44 | 3.58 | 0.671 |  | ***-*** | ***-*** | ***-*** | ***-*** |
|  | On the ground outside/bushes or grass | 43.8 (106) | 44.3 (339) | 0.93 | 0.67 | 1.30 | 0.676 |  | ***-*** | ***-*** | ***-*** | ***-*** |
|  | Don't know/refused | 0.4 (1) | 1.0 (8) | 0.39 | 0.05 | 3.38 | 0.394 |  | ***-*** | ***-*** | ***-*** | ***-*** |
| Defecates outside at home**^e^** | | 68.9 (166) | 67.3 (514) | 0.98 | 0.69 | 1.39 | 0.919 |  | ***-*** | ***-*** | ***-*** | ***-*** |
| Usual place of defecation at school | Toilet/latrine | 79.0 (192) | 85.0 (651) | Reference |  |  | * |  | Reference |  |  | ^ |
|  | On the ground outside/bushes or grass | 17.7 (43) | 11.5 (88) | 1.77 | 1.10 | 2.84 | 0.019 |  | 1.86 | 1.15 | 3.02 | 0.012 |
|  | Doesn't defecate at school | 1.7 (4) | 1.8 (14) | 1.08 | 0.31 | 3.70 | 0.904 |  | 1.03 | 0.30 | 3.54 | 0.965 |
|  | Don't know/refused | 1.7 (4) | 1.7 (13) | 0.93 | 0.28 | 3.10 | 0.906 |  | 0.88 | 0.26 | 2.96 | 0.836 |
| Defecates outside at school**^e^** | | 28.4 (69) | 24.3 (185) | 1.17 | 0.80 | 1.71 | 0.423 |  | ***-*** | ***-*** | ***-*** | ***-*** |
| Washes hands after defecating | Always | 41.6 (101) | 47.8 (367) | Reference |  |  | * |  |  |  |  | ^ |
|  | Sometimes | 49.4 (120) | 41.4 (318) | 1.28 | 0.92 | 1.79 | 0.144 |  | 1.36 | 0.96 | 1.92 | 0.086 |
|  | Never | 9.1 (22) | 10.7 (82) | 0.92 | 0.52 | 1.61 | 0.762 |  | 0.94 | 0.53 | 1.69 | 0.842 |
|  | Don’t know/refused | 0 (0) | 0.1 (1) | - | - | - | - |  | - | - | - | - |
| Washes hands before eating | Always | 36.4 (88) | 39.7 (305) | Reference |  |  |  |  | ***-*** | ***-*** | ***-*** | ***-*** |
|  | Sometimes | 55.8 (135) | 51.8 (398) | 1.06 | 0.75 | 1.49 | 0.737 |  | - | - | - | - |
|  | Never | 7.4 (18) | 8.3 (64) | 0.94 | 0.51 | 1.72 | 0.830 |  | ***-*** | ***-*** | ***-*** | ***-*** |
|  | Don’t know/refused | 0.4 (1) | 0.1 (1) | 2.86 | 0.16 | 51.77 | 0.476 |  | - | - | - | - |
| Wears shoes outside | Always | 65.8 (16) | 65.0 (498) | Reference |  | 0.00 | * |  | ***-*** | ***-*** | ***-*** | ***-*** |
|  | Sometimes | 30.9 (75) | 32.9 (252) | 0.94 | 0.66 | 1.34 | 0.747 |  | 0.95 | 0.66 | 1.36 | 0.788 |
|  | Never | 3.3 (8) | 2.0 (15) | 1.91 | 0.74 | 4.93 | 0.179 |  | 1.82 | 0.70 | 4.73 | 0.218 |
|  | Don’t know/refused | 0 (0) | 0.1 (1) | - | - | - | - |  | - | - | - | - |
| Wears shoes defecating | Always | 73.0 (176) | 73.2 (562) | Reference |  |  |  |  | - | - | - | - |
|  | Sometimes | 15.8 (38) | 16.9 (130) | 0.92 | 0.60 | 1.43 | 0.717 |  | ***-*** | ***-*** | ***-*** | ***-*** |
|  | Never | 10.4 (25) | 9.2 (71) | 1.21 | 0.72 | 2.04 | 0.475 |  | - | - | - | - |
|  | Don’t know/refused | 0.8 (2) | 0.7 (5) | 1.19 | 0.21 | 6.72 | 0.845 |  | ***-*** | ***-*** | ***-*** | ***-*** |
| ***Domain: Household WASH factors*** | | | | | | | | | | | | |
| Household water supply**^f^** | Improved | 76.8 (182) | 74.6 (579) | Reference |  |  | * |  | Reference |  |  |  |
|  | Unimproved | 22.8 (54) | 23.8 (185) | 0.94 | 0.64 | 1.37 | 0.741 |  | 0.96 | 0.65 | 1.41 | 0.823 |
|  | Don't know/refused | 0.4 (1) | 1.6 (12) | 0.26 | 0.03 | 2.04 | 0.198 |  | - | - | - | - |
| Household toilet/latrine**^g^** | No | 48.8 (118) | 48.6 (372) | Reference |  |  |  |  | Reference |  |  |  |
|  | Yes, but does not flush | 18.6 (45) | 17.2 (132) | 1.17 | 0.76 | 1.80 | 0.469 |  | - | - | - | - |
|  | Yes, flushes with water | 32.6 (79) | 34.2 (262) | 1.02 | 0.70 | 1.47 | 0.933 |  | ***-*** | ***-*** | ***-*** | ***-*** |
| Handwashing station**^h^** | No | 23.3 (56) | 15.9 (121) | Reference |  |  | * |  | Reference |  |  | ^ |
|  | Yes, but no soap available | 32.5 (78) | 36.5 (277) | 0.57 | 0.35 | 0.92 | 0.020 |  | 0.54 | 0.33 | 0.89 | 0.015 |
|  | Yes, soap available | 44.2 (106) | 47.6 (362) | 0.60 | 0.37 | 0.96 | 0.034 |  | 0.64 | 0.40 | 1.03 | 0.063 |
| ***Domain: Household socioeconomic factors*** | | | | | | | | | | | | |
| Mother's highest of level education | Never attended school | 21.9 (52) | 18.6 (144) | Reference |  |  | * |  | Reference |  |  |  |
|  | Completed or attended primary school | 43.0 (102) | 43.3 (336) | 0.82 | 0.54 | 1.25 | 0.364 |  | 0.84 | 0.55 | 1.27 | 0.407 |
|  | Completed or attended secondary school | 30.0 (71) | 32.4 (251) | 0.81 | 0.52 | 1.27 | 0.364 |  | 0.87 | 0.55 | 1.37 | 0.552 |
|  | Completed or attended tertiary education | 1.3 (3) | 3.1 (24) | 0.38 | 0.11 | 1.35 | 0.135 |  | 0.39 | 0.11 | 1.42 | 0.154 |
|  | Don't know/refused | 3.8 (9) | 2.7 (21) | 1.14 | 0.47 | 2.76 | 0.775 |  | 1.15 | 0.47 | 2.79 | 0.764 |
| Occupation main household income earner**^i^** | Farmer/animal keeper | 97.1 (230) | 97.4 (755) | Reference |  |  |  |  | - | - | - | - |
|  | Fisherman/market seller | 0.8 (2) | 0.5 (4) | 1.51 | 0.26 | 8.81 | 0.646 |  | ***-*** | ***-*** | ***-*** | ***-*** |
|  | Administration/healthcare worker/Other | 0.4 (1) | 0.7 (5) | 0.57 | 0.06 | 5.71 | 0.634 |  | - | - | - | - |
|  | Unemployed | 0.4 (1) | 0.7 (5) | 0.74 | 0.08 | 7.19 | 0.798 |  | ***-*** | ***-*** | ***-*** | ***-*** |
|  | Don't know/refused | 1.3 (3) | 0.8 (6) | 1.33 | 0.30 | 5.77 | 0.706 |  | - | - | - | - |
| Household income | Less than VND 20,000,000 | 56.1 (133) | 55.2 (428) | Reference |  |  |  |  | ***-*** | ***-*** | ***-*** | ***-*** |
|  | VND 20,000,000 – 50,000,000 | 14.8 (35) | 15.3 (119) | 0.94 | 0.60 | 1.47 | 0.781 |  | - | - | - | - |
|  | VND 50,000,000 – 200,000,000 | 3.0 (7) | 3.4 (26) | 0.67 | 0.27 | 1.66 | 0.389 |  | ***-*** | ***-*** | ***-*** | ***-*** |
|  | More than VND 200,000,000 | 0.4 (1) | 0.3 (2) | 1.26 | 0.10 | 15.11 | 0.857 |  | - | - | - | - |
|  | Don't know/refused | 25.7 (61) | 25.9 (201) | 1.06 | 0.73 | 1.54 | 0.749 |  | ***-*** | ***-*** | ***-*** | ***-*** |
| ***Domain: School location and WASH factors*** | | | | | | | | | | | | |
| Location**^c^** | Rural | 39.0 (98) | 43.7 (353) | Reference |  |  |  |  | ***-*** | ***-*** | ***-*** | ***-*** |
|  | Remote | 39.0 (98) | 33.1 (267) | 1.25 | 0.73 | 2.13 | 0.410 |  | - | - | - | - |
|  | Very remote | 21.9 (55) | 23.2 (187) | 0.94 | 0.46 | 1.89 | 0.859 |  | ***-*** | ***-*** | ***-*** | ***-*** |
| School water source**^j^** | Improved | 43.0 (108) | 32.2 (260) | Reference |  |  | * |  | Reference |  |  | ^ |
|  | Unimproved | 21.1 (53) | 23.1 (186) | 0.64 | 0.34 | 1.24 | 0.186 |  | 0.58 | 0.31 | 1.07 | 0.083 |
|  | No water | 21.9 (55) | 26.0 (210) | 0.66 | 0.37 | 1.19 | 0.165 |  | 0.75 | 0.42 | 1.34 | 0.327 |
|  | Don't know/refused | 13.9 (35) | 18.7 (151) | 0.56 | 0.29 | 1.09 | 0.090 |  | 0.64 | 0.33 | 1.21 | 0.165 |
| School toilets available to students | | 92.0 (231) | 94.7 (764) | 0.50 | 0.17 | 1.49 | 0.214 |  | ***-*** | ***-*** | ***-*** | ***-*** |
| School sanitation**^k^** | Improved | 94.0 (236) | 95.5 (771) | Reference |  |  |  |  | - | - | - | - |
|  | Unimproved | 6.0 (15) | 4.5 (36) | 1.81 | 0.48 | 6.83 | 0.383 |  | ***-*** | ***-*** | ***-*** | ***-*** |
| School handwahsing station**^l^** | No | 15.2 (38) | 7.7 (61) | Reference |  |  | * |  | Reference |  |  | ^ |
|  | Yes, but no soap | 66.0 (165) | 72.3 (576) | 0.37 | 0.17 | 0.77 | 0.008 |  | 0.37 | 0.18 | 0.79 | 0.010 |
|  | Yes, soap available | 18.8 (47) | 20.1 (160) | 0.32 | 0.13 | 0.76 | 0.010 |  | 0.34 | 0.15 | 0.78 | 0.011 |
| Latrine/toilet able to be locked | | 10.8 (27) | 11.9 (96) | 0.91 | 0.37 | 2.23 | 0.842 |  | ***-*** | ***-*** | ***-*** | ***-*** |
| Latrine/toilet observed to be clean | | 45.0 (1130 | 41.5 (335) | 1.18 | 0.72 | 1.93 | 0.509 |  | - | - | - | - |
| Latrine/toilet has water for cleaning self | | 44.2 (111) | 50.2 (405) | 0.75 | 0.46 | 1.22 | 0.239 |  | ***-*** | ***-*** | ***-*** | ***-*** |
| Latrine/toilet observed to be dirty | | 43.8 (110) | 46.0 (371) | 0.91 | 0.55 | 1.53 | 0.735 |  | ***-*** | ***-*** | ***-*** | ***-*** |
| Latrine/toilet observed to have flies | | 19.9 (50) | 22.8 (184) | 0.91 | 0.47 | 1.76 | 0.775 |  | - | - | - | - |
| Number of school toilets | | 5.3 ± 3.60 | 5.2 ± 3.44 | 1.00 | 0.94 | 1.07 | 0.883 |  | ***-*** | ***-*** | ***-*** | ***-*** |
| **Notes:** WASH = water, sanitation and hygiene. All hookworm = infection with at least one of *N. americanus, A, duodanale or A. ceylanicum*. aOR = adjusted odds ratio, OR = odds ratio, 95% CI = 95% confidence interval, STH = soil-transmitted helminths. Reference = reference category used for regression for categorical variables. **^†^**Numbers and proportion of qPCR positive and negative participants for each variable, except for continuous variables, reported as mean ± standard deviation. **^a^**Domain multivariable models conducted using covariates with p<0.2 on the Wald test from univariate analysis indicated by *. All domain level multivariate models included the covariates of sex and age. ^Denotes varibales from domain multivariable models with p<0.1 on Wald test and included in backwards stepwise elimination procedure to produce final multivariable models. **^b^**See Supplementary table 1 for list ethnicities grouped in 'Other'. **^c^**Captures students who report ever defecating outside at home or school. **^d,e^**Responses for variables collapsed to align (or approximate where not enough information available) with WHO and United Nations International Children’s Emergency Fund (UNICEF) Joint Monitoring Programme (JMP) for Water Supply and Sanitation definitions. **^d^**Improved = piped water, protected well, rainwater or public tap. Unimproved = unprotected well or surface water. **^e^**Improved = flush toilet or pit latrine with a slab. Unimproved = pit latrine without a slab. (-)Indicates categories not included in regression at that step. | | | | | | | | | | | | |

| **Supplementary table 9.** Sensitivity analysis adjusting for clustering at school and hamlet level: Factors associated with all hookworm and *Necator americanus* infection | | | | | | | | | |  |  |
| --- | --- | --- | --- | --- | --- | --- | --- | --- | --- | --- | --- |
|  |  |  | **All hookworm (N=6924^†^)** | | | |  | ***N. americanus* (N=6924^†^)** | | | |
| **Variable** |  | **% (n)*** | **aOR** | **95% CI** | | **p-value** |  | **aOR** | **95% CI** | | **p-value** |
| Age (years) |  | 7.8 ± 1.24 | 1.26 | 1.19 | 1.34 | <0.01 |  | 1.28 | 1.20 | 1.36 | <0.01 |
| Female |  | 51.4 (3578) | 0.55 | 0.47 | 0.65 | <0.01 |  | 0.54 | 0.46 | 0.64 | <0.01 |
| Ethnicity**^a^** | Kinh | 39.6 (2762) | Reference |  |  |  |  | Reference |  |  |  |
|  | Dao | 2.0 (140) | 3.19 | 1.57 | 6.50 | <0.01 |  | 3.58 | 1.70 | 7.53 | <0.01 |
|  | Gia-rai | 3.2 (225) | 8.88 | 4.49 | 17.59 | <0.01 |  | 10.58 | 5.23 | 21.43 | <0.01 |
|  | Hmông | 3.4 (235) | 22.87 | 12.64 | 41.37 | <0.01 |  | 26.50 | 14.32 | 49.05 | <0.01 |
|  | Mnông | 5.7 (399) | 8.02 | 4.45 | 14.45 | <0.01 |  | 8.59 | 4.67 | 15.82 | <0.01 |
|  | Nùng | 5.8 (494) | 3.34 | 2.00 | 5.56 | <0.01 |  | 3.80 | 2.23 | 6.48 | <0.01 |
|  | Tày | 5.8 (406) | 1.99 | 1.10 | 3.61 | 0.023 |  | 2.23 | 1.20 | 4.14 | 0.015 |
|  | Xơ-đăng | 1.2 (84) | 47.08 | 18.71 | 118.51 | <0.01 |  | 60.25 | 22.82 | 159.06 | <0.01 |
|  | Êđê | 29.4 (2048) | 9.46 | 6.50 | 13.77 | <0.01 |  | 10.54 | 7.10 | 15.65 | <0.01 |
|  | Other | 3.8 (265) | 3.25 | 1.81 | 5.81 | <0.01 |  | 3.66 | 2.00 | 6.70 | <0.01 |
| A. ceylanicum co-infection |  | 0.7 (49) | - | - | - | - |  | 4.56 | 2.30 | 9.05 | <0.01 |
| S. stercoralis co-infection |  | 0.5 (35) | 2.69 | 1.24 | 5.84 | 0.013 |  | 2.57 | 1.16 | 5.69 | 0.014 |
| Household water supply**^b^** | Improved | 85.1 (5916) | Reference |  |  |  |  | Reference |  |  |  |
|  | Unimproved | 13.1 (910) | 1.33 | 1.08 | 1.64 | <0.01 |  | 1.29 | 1.05 | 1.60 | 0.022 |
|  | Don't know/refused | 1.8 (126) | 0.94 | 0.47 | 1.87 | 0.863 |  | 0.97 | 0.49 | 1.95 | 0.902 |
| Household toilet available | No | 24.9 (1729) | Reference |  |  |  |  | Reference |  |  |  |
|  | Yes, but no flush | 14.9 (1034) | 0.84 | 0.67 | 1.06 | 0.148 |  | 0.80 | 0.63 | 1.02 | 0.115 |
|  | Yes, flushes | 60.2 (4183) | 0.56 | 0.46 | 0.68 | <0.01 |  | 0.55 | 0.45 | 0.67 | <0.01 |
| Mother/primary female carer's highest level of education | Never attended school | 8.0 (556) | Reference |  |  |  |  | Reference |  |  |  |
|  | Completed or attended primary school | 29.5 (2055) | 0.86 | 0.67 | 1.09 | 0.216 |  | 0.85 | 0.67 | 1.09 | 0.260 |
|  | Completed or attended secondary school | 49.6 (3452) | 0.67 | 0.52 | 0.86 | <0.01 |  | 0.65 | 0.50 | 0.84 | <0.01 |
|  | Completed or attended tertiary education | 9.2 (638) | 0.36 | 0.22 | 0.60 | <0.01 |  | 0.39 | 0.24 | 0.64 | <0.01 |
|  | Don't know/refused | 3.8 (262) | 0.54 | 0.33 | 0.88 | 0.013 |  | 0.57 | 0.35 | 0.92 | 0.023 |
| **Notes:** Models constructed for all hookworm (*N. americanus, A, duodanale and A. ceylanicum)* and *N. americanus* only, due to prevalence of all other soil-transmitted helminth species <1%. *Proportion out of total sample size that had data from all sources (demographic and school questionnaire) and were considered for multivariate analysis, N=6968. **^†^**Number of observations included in each multivariable model, not all 6968 observations included in analysis due to missingness of specific variables (0.6% of cases had incomplete data for the predictors in these models and therefore not included in the analyses). aOR = adjusted odds ratio, 95% CI = 95% confidence interval, Reference = reference category for categorical variable. **^a^**Response for variable collapsed to align with WHO and United Nations International Children’s Emergency Fund (UNICEF) Joint Monitoring Programme (JMP) for Water Supply and Sanitation definitions. Improved = piped water, protected well, rainwater or public tap. Unimproved = unprotected well or surface water. | | | | | | | | | | | |

| **Supplementary table 10.** Sensitivity analysis adjusting for clustering at school and hamlet level: Factors associated with moderate-to-heavy intensity *Necator americanus* infection | | | | | | |
| --- | --- | --- | --- | --- | --- | --- |
|  | |  |  | **Multivariate model (N=1047^†^)** | |  |
| **Variable** | | **% (n)*** | **aOR** | **95% CI** | | **p-value** |
| Age (years) |  | 8.2 +/- 1.36 | 1.13 | 1.01 | 1.26 | 0.030 |
| Female |  | 43.5 (438) | 1.11 | 0.82 | 1.51 | 0.504 |
| Ethnicity**^a^** | Kinh | 6.8 (72) | Reference |  |  |  |
|  | Dao | 1.8 (19) | 0.29 | 0.03 | 3.01 | 0.299 |
|  | Gia-rai | 8.9 (94) | 4.27 | 1.32 | 13.80 | 0.015 |
|  | Hmông | 11.8 (124) | 4.12 | 1.31 | 12.95 | 0.015 |
|  | Mnông | 11.7 (123) | 2.29 | 0.75 | 6.95 | 0.145 |
|  | Nùng | 3.9 (41) | 2.19 | 0.62 | 7.66 | 0.221 |
|  | Tày | 2.1 (22) | 2.57 | 0.57 | 11.59 | 0.218 |
|  | Xơ-đăng | 4.9 (52) | 5.09 | 1.36 | 19.07 | 0.016 |
|  | Êđê | 45.8 (482) | 3.24 | 1.30 | 8.07 | 0.012 |
|  | Other | 2.2 (23) | 3.40 | 0.84 | 13.70 | 0.085 |
| School handwashing station | No | 9.5 (99) | Reference |  |  |  |
|  | Yes, but no soap | 70.9 (738) | 0.29 | 0.14 | 0.61 | <0.01 |
|  | Yes, soap available | 19.6 (204) | 0.30 | 0.12 | 0.76 | 0.012 |
| **Notes:** Factors investigated comparing moderate or heavy intensity infection to light intensity infection. *Proportion out of total sample size that had data from all sources (demographic and school questionnaire) and were considered for multivariate analysis, N=1052. **^†^**Number of observations included in each multivariable model, not all 1052 observations included in analysis due to missingness of specific variables (0.5% of cases had incomplete data for the predictors in these models and therefore not included in the analyses). Infection intensity classes are according to WHO guidelines for classifying infection intensity based on EPG values; Light intensity infection: N. americanus (1 – 1999 epg) moderate: N. americanus (2000 - 3999 epg); heavy: N. americanus (≥4000 epg). Moderate and heavy intensity analysed as a single group to align with WHO 2030 targets STH control program monitoring targets. **^a^**Appendix 4 for list ethnicities grouped in 'Other'. | | | | | | |

| **Supplementary table 11.** Sensitivity analysis adjusting for clustering at school and hamlet level: Factors associated with all hookworm, univariate and domain level multivariate analysis | | | | | | | | | | | | |
| --- | --- | --- | --- | --- | --- | --- | --- | --- | --- | --- | --- | --- |
|  |  | **qPCR positive n=1085^†^** | **qPCR negative n=6550^†^** | **Univariate analysis** | | | |  | **Domain Multivariate analysis^a^** | | | |
| ***Variables*** |  | **% (n)** | **% (n)** | **OR** | **95% CI** | | **p-value** |  | **aOR** | **95% CI** | | **p-value** |
| ***Domain: demographics*** | | | | | | | | | | | | |
| Age (years) |  | 8.2 ± 1.36 | 7.8 ± 1.20 | 1.25 | 1.18 | 1.32 | <0.01* |  | 1.56 | 1.38 | 1.76 | <0.01^# |
| Female |  | 43.9 (476) | 52.4 (3430) | 0.60 | 0.52 | 0.69 | <0.01* |  | 0.59 | 0.51 | 0.68 | <0.01^ |
| Ethnicity**^b^** | Kinh | 7.5 (81) | 45.8 (3002) | Reference |  |  | * |  | Reference |  |  | ^ |
|  | Dao | 1.8 (20) | 2.3 (148) | 5.39 | 2.84 | 10.24 | <0.01 |  | 4.31 | 2.24 | 8.29 | <0.01 |
|  | Gia-rai | 8.7 (94) | 2.3 (151) | 11.94 | 6.06 | 23.55 | <0.01 |  | 11.46 | 5.79 | 22.67 | <0.01 |
|  | Hmông | 11.6 (126) | 1.9 (121) | 29.57 | 16.71 | 52.32 | <0.01 |  | 29.12 | 16.41 | 51.67 | <0.01 |
|  | Mnông | 11.5 (125) | 4.6 (304) | 9.97 | 5.55 | 17.90 | <0.01 |  | 9.42 | 5.20 | 17.07 | <0.01 |
|  | Nùng | 4.0 (43) | 6.1 (402) | 3.64 | 2.25 | 5.88 | <0.01 |  | 3.63 | 2.24 | 5.87 | <0.01 |
|  | Tày | 2.2 (24) | 6.5 (427) | 2.23 | 1.29 | 3.85 | <0.01 |  | 2.23 | 1.28 | 3.86 | <0.01 |
|  | Xơ-đăng | 4.8 (52) | 0.7 (45) | 71.22 | 29.08 | 174.39 | <0.01 |  | 71.78 | 29.55 | 174.34 | <0.01 |
|  | Êđê | 45.7 (496) | 25.7 (1685) | 11.38 | 7.98 | 16.23 | <0.01 |  | 11.10 | 7.76 | 15.89 | <0.01 |
|  | Other | 2.2 (24) | 4.1 (265) | 3.42 | 1.94 | 6.00 | <0.01 |  | 2.96 | 1.68 | 5.23 | <0.01 |
| School graded | Grade 1 | 23.1 (251) | 26.8 (1752) | Reference |  |  | * |  | Reference |  |  | ^# |
|  | Grade 2 | 24.6 (267) | 28.0 (1831) | 1.17 | 0.96 | 1.44 | 0.127 |  | 0.72 | 0.57 | 0.93 | 0.011 |
|  | Grade 3 | 26.0 (282) | 23.7 (1552) | 1.37 | 1.11 | 1.68 | <0.01 |  | 0.55 | 0.39 | 0.76 | <0.01 |
|  | Grade 4 | 26.3 (285) | 21.6 (1415) | 1.55 | 1.26 | 1.91 | <0.01 |  | 0.39 | 0.25 | 0.60 | <0.01 |
| ***Domain: co-infection with other STH species*** | | | | | | | | | | | | |
| *A. lumbricoides* | | 0.5 (5) | 0.2 (14) | 1.88 | 0.56 | 6.39 | 0.309 |  | - | - | - | - |
| *T. trichiura* |  | 1.0 (11) | 0.7 (47) | 0.88 | 0.41 | 1.87 | 0.736 |  | - | - | - | - |
| *S. stercoralis* |  | 2.1 (23) | 0.3 (19) | 3.70 | 1.85 | 7.42 | <0.01* |  | 3.59 | 1.76 | 7.30 | <0.01^ |
| ***Domain: Health, personal hygiene and defecation behaviour*** | | | | | | | | | | | | |
| Had deworming within the last year | No | 52.0 (539) | 51.0 (3166) | Reference |  |  |  |  | - | - | - | - |
|  | Yes | 45.0 (467) | 45.6 (2830) | 0.96 | 0.81 | 1.15 | 0.669 |  | - | - | - | - |
|  | Don't know/refused | 3.0 (31) | 3.5 (217) | 0.75 | 0.48 | 1.17 | 0.201 |  | - | - | - | - |
| Usual place of defecation at home**^c^** | Household toilet/latrine | 54.0 (559) | 79.7 (4952) | Reference |  |  | * |  | Reference |  |  | ^ |
|  | Shared toilet/latrine | 1.7 (18) | 1.2 (75) | 1.12 | 0.61 | 2.04 | 0.716 |  | 1.17 | 0.63 | 2.17 | 0.615 |
|  | On the ground outside/bushes or grass | 43.4 (449) | 18.0 (1120) | 1.60 | 1.34 | 1.92 | 0.000 |  | 1.39 | 1.12 | 1.74 | <0.01 |
|  | Other/Don't know/refused | 0.9 (9) | 1.0 (64) | 1.29 | 0.60 | 2.79 | 0.519 |  | 1.20 | 0.53 | 2.68 | 0.664 |
| Ever defecates outside at home**^d^** | | 67.1 (692) | 42.8 (2643) | 1.44 | 1.22 | 1.70 | <0.01* |  | 1.18 | 0.96 | 1.45 | 0.110 |
| Usual place of defecation at school | Toilet/latrine | 83.7 (867) | 92.4 (5726) | Reference |  |  | * |  | Reference |  |  |  |
|  | On the ground outside/bushes or grass | 12.7 (132) | 4.3 (264) | 1.48 | 1.06 | 2.07 | 0.021 |  | 1.37 | 0.91 | 2.06 | 0.131 |
|  | Doesn't defecate at school | 1.8 (18) | 2.3 (140) | 1.11 | 0.61 | 2.00 | 0.738 |  | 1.05 | 0.57 | 1.94 | 0.875 |
|  | Don't know/refused | 1.8 (19) | 1.1 (67) | 1.33 | 0.70 | 2.52 | 0.390 |  | 1.38 | 0.68 | 2.81 | 0.376 |
| Ever defecates outside at school**^d^** | | 25.0 (258) | 12.3 (762) | 1.25 | 1.01 | 1.55 | 0.040 |  | 1.09 | 0.84 | 1.42 | 0.521 |
| Washes hands after defecating | Always | 46.8 (486) | 62.1 (3855) | Reference |  |  | * |  | Reference |  |  | ^ |
|  | Sometimes | 43.0 (446) | 32.6 (2023) | 1.12 | 0.95 | 1.32 | 0.189 |  | 1.14 | 0.93 | 1.40 | 0.210 |
|  | Never | 10.1 (105) | 5.3 (326) | 1.41 | 1.05 | 1.90 | 0.023 |  | 1.37 | 0.96 | 1.94 | 0.084 |
|  | Don’t know/refused | 0.1 (1) | 0.1 (7) | 0.35 | 0.04 | 3.39 | 0.367 |  | 0.24 | 0.02 | 2.53 | 0.237 |
| Washes hands before eating | Always | 39.5 (410) | 53.8 (3340) | Reference |  |  | * |  | Reference |  |  |  |
|  | Sometimes | 52.4 (543) | 40.4 (2503) | 1.12 | 0.95 | 1.33 | 0.173 |  | 1.06 | 0.86 | 1.29 | 0.606 |
|  | Never | 7.9 (82) | 5.7 (352) | 1.38 | 1.01 | 1.88 | 0.040 |  | 1.04 | 0.72 | 1.50 | 0.840 |
|  | Don’t know/refused | 0.2 (2) | 0.1 (8) | 1.52 | 0.27 | 8.64 | 0.638 |  | 1.82 | 0.28 | 12.04 | 0.532 |
| Wears shoes outside | Always | 64.4 (677) | 73.6 (4573) | Reference |  |  | * |  | Reference |  |  |  |
|  | Sometimes | 32.3 (335) | 24.6 (1529) | 1.19 | 1.00 | 1.43 | 0.053 |  | 1.21 | 0.98 | 1.48 | 0.073 |
|  | Never | 2.2 (23) | 1.6 (101) | 1.22 | 0.71 | 2.09 | 0.472 |  | 1.27 | 0.72 | 2.24 | 0.414 |
|  | Don’t know/refused | 0.1 (1) | 0.2 (10) | 0.76 | 0.09 | 6.28 | 0.795 |  | 0.91 | 0.09 | 9.09 | 0.935 |
| Wears shoes defecating | Always | 73.1 (757) | 66.0 (4102) | Reference |  |  | * |  | Reference |  |  | ^ |
|  | Sometimes | 16.6 (172) | 18.7 (1161) | 0.87 | 0.71 | 1.08 | 0.204 |  | 0.79 | 0.62 | 1.00 | 0.047 |
|  | Never | 9.7 (100) | 14.6 (906) | 0.76 | 0.59 | 0.98 | 0.036 |  | 0.72 | 0.55 | 0.95 | 0.021 |
|  | Don’t know/refused | 0.7 (7) | 0.8 (47) | 0.85 | 0.36 | 2.02 | 0.714 |  | 0.72 | 0.28 | 1.84 | 0.496 |
| ***Domain: Household WASH factors*** | | | | | | | | | | | | |
| Household water supply**^e^** | Improved | 75.1 (779) | 86.8 (5396) | Reference |  |  |  |  | Reference |  |  | ^ |
|  | Unimproved | 23.7 (246) | 11.3 (701) | 1.42 | 1.16 | 1.74 | <0.01 |  | 1.46 | 1.18 | 1.80 | <0.01 |
|  | Don't know/refused | 1.3 (13) | 1.9 (118) | 1.04 | 0.55 | 1.96 | 0.904 |  | 0.96 | 0.49 | 1.88 | 0.915 |
| Household toilet/latrine**^f^** | No | 47.8 (495) | 20.9 (1296) | Reference |  |  | * |  | Reference |  |  | ^ |
|  | Yes, but does not flush | 18.1 (187) | 14.2 (882) | 0.82 | 0.65 | 1.04 | 0.104 |  | 0.83 | 0.66 | 1.05 | 0.121 |
|  | Yes, flushes with water | 34.1 (353) | 64.9 (4024) | 0.49 | 0.40 | 0.60 | <0.01 |  | 0.49 | 0.40 | 0.60 | <0.01 |
| Handwashing station**^g^** | No | 17.4 (178) | 7.4 (457) | Reference |  |  | * |  | Reference |  |  | ^ |
|  | Yes, but no soap available | 35.7 (366) | 1424 (23.1) | 0.87 | 0.66 | 1.15 | 0.338 |  | 0.88 | 0.66 | 1.16 | 0.355 |
|  | Yes, soap available | 47.0 (482) | 69.5 (4288) | 0.75 | 0.57 | 0.99 | 0.040 |  | 0.75 | 0.57 | 0.99 | 0.042 |
| ***Domain: Household socioeconomic factors*** | | | | | | | | | | | | |
| Mother's highest level of education | Never attended school | 19.1 (198) | 6.2 (384) | Reference |  |  | * |  | Reference |  |  | ^ |
|  | Completed or attended primary school | 43.1 (447) | 27.2 (1693) | 0.74 | 0.58 | 0.93 | 0.010 |  | 0.77 | 0.61 | 0.98 | 0.032 |
|  | Completed or attended secondary school | 32.5 (337) | 52.6 (3276) | 0.47 | 0.37 | 0.60 | <0.01 |  | 0.52 | 0.40 | 0.66 | <0.01 |
|  | Completed or attended tertiary education | 2.5 (26) | 10.2 (632) | 0.22 | 0.14 | 0.34 | <0.01 |  | 0.31 | 0.19 | 0.50 | <0.01 |
|  | Don't know/refused | 2.9 (30) | 3.9 (243) | 0.46 | 0.29 | 0.72 | <0.01 |  | 0.47 | 0.29 | 0.75 | <0.01 |
| Occupation main household income earner**^h^** | Farmer/animal keeper | 97.1 (1007) | 91.6 (5706) | Reference |  |  | * |  | Reference |  |  | ^ |
|  | Fisherman/market seller | 0.7 (7) | 2.2 (134) | 0.61 | 0.27 | 1.38 | 0.236 |  | 0.70 | 0.31 | 1.61 | 0.402 |
|  | Administration/healthcare worker/Other | 0.7 (7) | 4.7 (293) | 0.25 | 0.12 | 0.55 | <0.01 |  | 0.43 | 0.19 | 0.99 | 0.047 |
|  | Unemployed | 0.6 (6) | 0.5 (30) | 1.48 | 0.57 | 3.84 | 0.418 |  | 1.46 | 0.55 | 3.92 | 0.449 |
|  | Don't know/refused | 1.0 (10) | 1.1 (66) | 1.00 | 0.46 | 2.16 | 0.993 |  | 1.09 | 0.49 | 2.41 | 0.840 |
| Household income | Less than VND 20,000,000 | 55.8 (579) | 43.2 (2689) | Reference |  |  | * |  | Reference |  |  | ^ |
|  | VND 20,000,000 – 50,000,000 | 15.1 (157) | 27.9 (1737) | 0.70 | 0.56 | 0.86 | <0.01 |  | 0.78 | 0.62 | 0.97 | 0.023 |
|  | VND 50,000,000 – 200,000,000 | 3.3 (34) | 7.7 (481) | 0.59 | 0.39 | 0.89 | 0.012 |  | 0.75 | 0.49 | 1.16 | 0.195 |
|  | More than VND 200,000,000 | 0.3 (3) | 0.7 (43) | 0.76 | 0.21 | 2.76 | 0.672 |  | 0.95 | 0.26 | 3.53 | 0.945 |
|  | Don't know/refused | 25.5 (265) | 20.5 (1275) | 1.02 | 0.85 | 1.23 | 0.804 |  | 0.96 | 0.80 | 1.17 | 0.709 |
| ***Domain: School location and WASH factors*** | | | | | | | | | | | | |
| Location**^i^** | Rural | 42.9 (465) | 58.8 (3849) | Reference |  |  | * |  | Reference |  |  | ^ |
|  | Remote | 34.7 (376) | 32.4 (2124) | 1.82 | 0.94 | 3.56 | 0.078 |  | 1.78 | 0.93 | 3.39 | 0.081 |
|  | Very remote | 22.5 (244) | 8.8 (577) | 5.07 | 1.89 | 13.64 | <0.01 |  | 4.88 | 1.72 | 13.85 | <0.01 |
| School water source**^j^** | Improved | 35.0 (380) | 43.9 (2875) | Reference |  |  | * |  | Reference |  |  | ^ |
|  | Unimproved | 22.2 (241) | 12.9 (846) | 2.72 | 1.03 | 7.15 | 0.043 |  | 2.04 | 0.83 | 5.01 | 0.118 |
|  | No water | 25.0 (271) | 27.7 (1814) | 1.21 | 0.55 | 2.64 | 0.640 |  | 0.70 | 0.34 | 1.45 | 0.341 |
|  | Don't know/refused | 17.8 (193) | 15.5 (1015) | 1.89 | 0.74 | 4.82 | 0.183 |  | 1.60 | 0.70 | 3.66 | 0.261 |
| School toilets available to students | | 94.1 (1021) | 97.4 (6380) | 0.31 | 0.05 | 1.86 | 0.202 |  | - | - | - | - |
| School sanitation**^k^** | Improved | 95.2 (1033) | 95.3 (6239) | Reference |  |  |  |  | - | - | - | - |
|  | Unimproved | 4.8 (52) | 4.8 (311) | 0.58 | 0.11 | 2.96 | 0.509 |  | - | - | - | - |
| School handwashing station**^l^** | No | 9.6 (103) | 11.8 (746) | Reference |  |  | * |  | Reference |  |  | ^ |
|  | Yes, but no soap | 70.8 (760) | 53.9 (3411) | 1.97 | 0.74 | 5.22 | 0.173 |  | 3.10 | 1.17 | 8.23 | 0.023 |
|  | Yes, soap available | 19.6 (210) | 34.3 (2167) | 0.58 | 0.20 | 1.65 | 0.307 |  | 0.98 | 0.34 | 2.84 | 0.972 |
| Latrine/toilet able to be locked | | 11.5 (125) | 5.3 (350) | 2.06 | 0.54 | 7.87 | 0.291 |  | - | - | - | - |
| Latrine/toilet observed to be clean | | 42.9 (465) | 61.7 (4044) | 0.52 | 0.27 | 0.99 | 0.048* |  | 0.61 | 0.28 | 1.36 | 0.229 |
| Latrine/toilet has water for cleaning self | | 48.9 (531) | 48.5 (3174) | 1.15 | 0.59 | 2.22 | 0.687 |  | - | - | - | - |
| Latrine/toilet observed to be dirty | | 45.2 (490) | 29.5 (1930) | 1.59 | 0.79 | 3.20 | 0.197* |  | 0.59 | 0.25 | 1.39 | 0.228 |
| Latrine/toilet observed to have flies | | 21.8 (237) | 14.8 (967) | 1.26 | 0.52 | 3.04 | 0.612 |  | - | - | - | - |
| Number of school toilets | | 5.3 ± 3.56 | 5.2 ± 3.87 | 1.02 | 0.94 | 1.11 | 0.635 |  | - | - | - | - |
| **Notes:** WASH = water, sanitation and hygiene. All hookworm = infection with at least one of *N. americanus, A, duodanale or A. ceylanicum*. aOR = adjusted odds ratio, OR = odds ratio, 95% CI = 95% confidence interval, STH = soil-transmitted helminths. Reference = reference category used for regression for categorical variables. **^†^**Numbers and proportion of qPCR positive and negative participants for each variable, except for continuous variables, reported as mean ± standard deviation. **^a^**Domain multivariable models conducted using covariates with p<0.2 on the Wald test from univariate analysis indicated by *. All domain level multivariate models included the covariates of sex and age. ^Denotes varibales from domain multivariable models with p<0.1 on Wald test and included in backwards stepwise elimination procedure to produce final multivariable models. **^b^**See Supplementary table 1 for list ethnicities grouped in 'Other'. **^c^**Captures students who report ever defecating outside at home or school. **^d,e^**Responses for variables collapsed to align (or approximate where not enough information available) with WHO and United Nations International Children’s Emergency Fund (UNICEF) Joint Monitoring Programme (JMP) for Water Supply and Sanitation definitions. **^d^**Improved = piped water, protected well, rainwater or public tap. Unimproved = unprotected well or surface water. **^e^**Improved = flush toilet or pit latrine with a slab. Unimproved = pit latrine without a slab. (-)Indicates categories not included in regression at that step. #Age and grade level found to be only variables collinear (VIF>5) in initial final multivariable model. Initial models tested with age and grade independently and the model with age had a lower AIC than that with grade. Grade was dropped from the analysis at this stage prior to stepwise regression. | | | | | | | | | | | | |

| **Supplementary table 12.** Sensitivity analysis adjusting for clustering at school and hamlet level: Factors associated with *Necator americanus*, univariate and domain level multivariate analysis | | | | | | | | | | | | | | |
| --- | --- | --- | --- | --- | --- | --- | --- | --- | --- | --- | --- | --- | --- | --- |
|  |  | **qPCR positive n=1052^†^** | | **qPCR negative n=6583^†^** | | **Univariate analysis** | | | |  | **Domain Multivariate analysis^a^** | | | |
| ***Variables*** |  | **% (n)** | | **% (n)** | | **OR** | **95% CI** | | **p-value** |  | **aOR** | **95% CI** | | **p-value** |
| ***Domain: demographics*** | | | | | | | | | | | | | | |
| Age (years) |  | 8.2 | (1) | 7.8 | (1) | 1.27 | 1.20 | 1.34 | <0.01* |  | 1.56 | 1.37 | 1.76 | <0.01^# |
| Female |  | 43.54 | (458) | 52.38 | (3448) | 0.59 | 0.50 | 0.68 | <0.01* |  | 0.57 | 0.49 | 0.67 | <0.01^ |
| Ethnicity**^b^** | Kinh | 6.84 | (72) | 45.74 | (3011) | Reference |  |  | * |  | Reference |  |  | ^ |
|  | Dao | 1.81 | (19) | 2.26 | (149) | 6.33 | 3.26 | 12.28 | <0.01 |  | 5.03 | 2.56 | 9.88 | <0.01 |
|  | Gia-rai | 8.94 | (94) | 2.29 | (151) | 13.40 | 6.66 | 26.95 | <0.01 |  | 13.03 | 6.45 | 26.34 | <0.01 |
|  | Hmông | 11.79 | (124) | 1.87 | (123) | 32.30 | 17.92 | 58.24 | <0.01 |  | 31.98 | 17.66 | 57.92 | <0.01 |
|  | Mnông | 11.69 | (123) | 4.65 | (306) | 10.54 | 5.75 | 19.31 | <0.01 |  | 9.92 | 5.36 | 18.36 | <0.01 |
|  | Nùng | 3.9 | (41) | 6.14 | (404) | 4.00 | 2.43 | 6.59 | <0.01 |  | 4.00 | 2.43 | 6.59 | <0.01 |
|  | Tày | 2.09 | (22) | 6.52 | (429) | 2.31 | 1.30 | 4.10 | <0.01 |  | 2.30 | 1.29 | 4.10 | <0.01 |
|  | Xơ-đăng | 4.94 | (52) | 0.68 | (45) | 85.72 | 33.76 | 217.68 | <0.01 |  | 87.56 | 34.65 | 221.25 | <0.01 |
|  | Êđê | 45.82 | (482) | 25.81 | (1699) | 12.42 | 8.57 | 18.02 | <0.01 |  | 12.18 | 8.37 | 17.74 | <0.01 |
|  | Other | 2.19 | (23) | 4.04 | (266) | 3.76 | 2.10 | 6.72 | <0.01 |  | 3.26 | 1.81 | 5.85 | <0.01 |
| School graded | Grade 1 | 23 | (238) | 26.81 | (1765) | Reference |  |  | * |  | Reference |  |  | ^# |
|  | Grade 2 | 24.62 | (259) | 27.94 | (1839) | 1.22 | 0.99 | 1.50 | 0.069 |  | 0.75 | 0.59 | 0.97 | 0.028 |
|  | Grade 3 | 26.33 | (277) | 23.65 | (1557) | 1.43 | 1.16 | 1.77 | <0.01 |  | 0.58 | 0.41 | 0.81 | <0.01 |
|  | Grade 4 | 26.43 | (278) | 21.6 | (1422) | 1.62 | 1.31 | 2.01 | <0.01 |  | 0.41 | 0.26 | 0.64 | <0.01 |
| ***Domain: co-infection with other STH species*** | | | | | | | | | | | | | | |
| *A. lumbricoides* | | 0.48 | (5) | 0.21 | (14) | 1.95 | 0.57 | 6.64 | 0.288 |  | - | - | - | - |
| *T. trichiura* |  | 0.95 | (10) | 0.73 | (48) | 0.78 | 0.35 | 1.70 | 0.528 |  | - | - | - | - |
| *S. stercoralis* |  | 2.19 | (23) | 0.29 | (19) | 3.77 | 1.87 | 7.60 | <0.01* |  | 3.47 | 1.68 | 7.16 | <0.01^ |
| *A. ceylanicum* | | 2.19 | (23) | 0.44 | (29) | 5.29 | 2.80 | 9.97 | <0.01* |  | 5.03 | 2.62 | 9.66 | <0.01^ |
| ***Domain: Health, personal hygiene and defecation behaviour*** | | | | | | | | | | | | | | |
| Had deworming within the last year | No | 51.64 | (519) | 51.02 | (3186) | Reference |  |  |  |  | - | - | - | - |
|  | Yes | 45.27 | (455) | 45.51 | (2842) | 0.99 | 0.82 | 1.18 | 0.893 |  | - | - | - | - |
|  | Don't know/refused | 3.08 | (31) | 3.47 | (217) | 0.79 | 0.51 | 1.23 | 0.304 |  | - | - | - | - |
| Usual place of defecation at home**^c^** | Household toilet/latrine | 53.04 | (532) | 79.75 | (4979) | Reference |  |  | * |  | Reference |  |  | ^ |
|  | Shared toilet/latrine | 1.79 | (18) | 1.2 | (75) | 1.18 | 0.64 | 2.16 | 0.598 |  | 1.24 | 0.67 | 2.32 | 0.494 |
|  | On the ground outside/bushes or grass | 44.27 | (444) | 18.02 | (1125) | 1.65 | 1.38 | 1.98 | <0.01 |  | 1.43 | 1.14 | 1.79 | <0.01 |
|  | Other/Don't know/refused | 0.9 | (9) | 1.03 | (64) | 1.38 | 0.64 | 3.00 | 0.415 |  | 1.27 | 0.56 | 2.86 | 0.567 |
| Ever defecates outside at home**^d^** | | 67.8 | (678) | 42.76 | (2657) | 1.47 | 1.24 | 1.74 | <0.01* |  | 1.19 | 0.97 | 1.47 | 0.102 |
| Usual place of defecation at school | Toilet/latrine | 83.47 | (838) | 92.39 | (5755) | Reference |  |  | * |  | Reference |  |  | ^ |
|  | On the ground outside/bushes or grass | 13.05 | (131) | 4.25 | (265) | 1.56 | 1.11 | 2.19 | 0.010 |  | 1.43 | 0.95 | 2.17 | 0.089 |
|  | Doesn't defecate at school | 1.79 | (18) | 2.25 | (140) | 1.17 | 0.64 | 2.13 | 0.606 |  | 1.10 | 0.59 | 2.05 | 0.758 |
|  | Don't know/refused | 1.69 | (17) | 1.11 | (69) | 1.17 | 0.60 | 2.30 | 0.646 |  | 1.16 | 0.55 | 2.46 | 0.697 |
| Ever defecates outside at school**^d^** | | 25.37 | (254) | 12.33 | (766) | 1.28 | 1.03 | 1.60 | 0.025* |  | 1.10 | 0.84 | 1.44 | 0.491 |
| Washes hands after defecating | Always | 46.42 | (467) | 62.05 | (3874) | Reference |  |  | * |  | Reference |  |  |  |
|  | Sometimes | 43.14 | (434) | 32.6 | (2035) | 1.12 | 0.95 | 1.32 | 0.189 |  | 1.13 | 0.91 | 1.39 | 0.261 |
|  | Never | 10.34 | (104) | 5.24 | (327) | 1.41 | 1.05 | 1.90 | 0.023 |  | 1.35 | 0.94 | 1.92 | 0.105 |
|  | Don’t know/refused | 0.1 | (1) | 0.11 | (7) | 0.35 | 0.04 | 3.39 | 0.367 |  | 0.24 | 0.02 | 2.48 | 0.231 |
| Washes hands before eating | Always | 39.1 | (393) | 53.84 | (3357) | Rreference |  |  | * |  | Referece |  |  |  |
|  | Sometimes | 52.54 | (528) | 40.38 | (2518) | 1.12 | 0.95 | 1.33 | 0.188 |  | 1.06 | 0.86 | 1.31 | 0.575 |
|  | Never | 8.16 | (82) | 5.65 | (352) | 1.46 | 1.07 | 1.99 | 0.017 |  | 1.10 | 0.76 | 1.59 | 0.630 |
|  | Don’t know/refused | 0.2 | (2) | 0.13 | (8) | 1.64 | 0.28 | 9.45 | 0.581 |  | 1.92 | 0.28 | 13.08 | 0.504 |
| Wears shoes outside | Always | 65.24 | (655) | 73.58 | (4595) | Reference |  |  | * |  | Reference |  |  | ^ |
|  | Sometimes | 32.37 | (325) | 24.64 | (1539) | 1.19 | 0.99 | 1.43 | 0.066 |  | 1.20 | 0.97 | 1.48 | 0.089 |
|  | Never | 2.29 | (23) | 1.62 | (101) | 1.31 | 0.76 | 2.26 | 0.325 |  | 1.35 | 0.76 | 2.41 | 0.304 |
|  | Don’t know/refused | 0.1 | (1) | 0.16 | (10) | 0.83 | 0.10 | 6.95 | 0.866 |  | 0.93 | 0.09 | 9.49 | 0.950 |
| Wears shoes defecating | Always | 73.21 | (735) | 66.01 | (4124) | Reference |  |  | * |  | Reference |  |  | ^ |
|  | Sometimes | 16.53 | (166) | 18.68 | (1167) | 0.86 | 0.70 | 1.07 | 0.187 |  | 0.78 | 0.61 | 0.99 | 0.045 |
|  | Never | 9.56 | (96) | 14.56 | (910) | 0.77 | 0.59 | 1.00 | 0.050 |  | 0.73 | 0.55 | 0.96 | 0.026 |
|  | Don’t know/refused | 0.7 | (7) | 0.75 | (47) | 0.92 | 0.39 | 2.18 | 0.845 |  | 0.77 | 0.30 | 1.97 | 0.583 |
| ***Domain: Household WASH factors*** | | | | | | | | | | | | | | |
| Household water supply**^e^** | Improved | 75.07 | (756) | 86.76 | (5419) | Reference |  |  |  |  | Reference |  |  | ^ |
|  | Unimproved | 23.63 | (238) | 11.35 | (709) | 1.38 | 1.13 | 1.69 | <0.01 |  | 1.41 | 1.14 | 1.75 | <0.01 |
|  | Don't know/refused | 1.29 | (13) | 1.89 | (118) | 1.10 | 0.58 | 2.07 | 0.777 |  | 1.02 | 0.52 | 2.00 | 0.959 |
| Household toilet/latrine**^f^** | No | 49 | (489) | 21 | (1302) | Reference |  |  | * |  | Reference |  |  | ^ |
|  | Yes, but does not flush | 17.65 | (177) | 14.31 | (892) | 0.80 | 0.64 | 1.00 | 0.054 |  | 0.79 | 0.62 | 1.01 | 0.055 |
|  | Yes, flushes with water | 33.6 | (337) | 64.81 | (4040) | 0.50 | 0.41 | 0.60 | <0.01 |  | 0.47 | 0.38 | 0.59 | <0.01 |
| Handwashing station**^g^** | No | 17.79 | (177) | 7.39 | (458) | Reference |  |  | * |  | Reference |  |  | ^ |
|  | Yes, but no soap available | 35.58 | (354) | 23.16 | (1436) | 0.88 | 0.67 | 1.16 | 0.376 |  | 0.84 | 0.63 | 1.12 | 0.236 |
|  | Yes, soap available | 46.63 | (464) | 69.45 | (4306) | 0.65 | 0.50 | 0.84 | 0.040 |  | 0.74 | 0.56 | 0.98 | 0.037 |
| ***Domain: Household socioeconomic factors*** | | | | | | | | | | | | | | |
| Mother's highest level of education | Never attended school | 19.46 | (196) | 6.17 | (386) | Reference |  |  | * |  | Reference |  |  | ^ |
|  | Completed or attended primary school | 43.3 | (436) | 27.22 | (1704) | 0.73 | 0.58 | 0.92 | <0.01 |  | 0.77 | 0.60 | 0.97 | 0.029 |
|  | Completed or attended secondary school | 31.68 | (319) | 52.63 | (3294) | 0.46 | 0.36 | 0.58 | <0.01 |  | 0.51 | 0.39 | 0.65 | <0.01 |
|  | Completed or attended tertiary education | 2.58 | (26) | 10.1 | (632) | 0.23 | 0.14 | 0.36 | <0.01 |  | 0.34 | 0.21 | 0.55 | <0.01 |
|  | Don't know/refused | 2.98 | (30) | 3.88 | (243) | 0.47 | 0.30 | 0.75 | <0.01 |  | 0.48 | 0.30 | 0.78 | <0.01 |
| Occupation main household income earner**^h^** | Farmer/animal keeper | 97.42 | (980) | 91.58 | (5733) | Reference |  |  | * |  | Reference |  |  | ^ |
|  | Fisherman/market seller | 0.6 | (6) | 2.16 | (135) | 0.55 | 0.23 | 1.32 | 0.180 |  | 0.62 | 0.26 | 1.51 | 0.295 |
|  | Administration/healthcare worker/Other | 0.5 | (5) | 4.71 | (295) | 0.19 | 0.08 | 0.47 | <0.01 |  | 0.30 | 0.12 | 0.78 | 0.014 |
|  | Unemployed | 0.6 | (6) | 0.48 | (30) | 1.54 | 0.59 | 4.02 | 0.374 |  | 1.50 | 0.56 | 4.05 | 0.421 |
|  | Don't know/refused | 0.89 | (9) | 1.07 | (67) | 0.89 | 0.40 | 2.02 | 0.785 |  | 0.95 | 0.41 | 2.18 | 0.897 |
| Household income | Less than VND 20,000,000 | 55.61 | (560) | 43.29 | (2708) | Reference |  |  | * |  | Reference |  |  | ^ |
|  | VND 20,000,000 – 50,000,000 | 15 | (151) | 27.86 | (1743) | 0.70 | 0.57 | 0.88 | <0.01 |  | 0.78 | 0.63 | 0.98 | 0.033 |
|  | VND 50,000,000 – 200,000,000 | 3.28 | (33) | 7.7 | (482) | 0.61 | 0.40 | 0.92 | 0.019 |  | 0.78 | 0.50 | 1.20 | 0.255 |
|  | More than VND 200,000,000 | 0.3 | (3) | 0.69 | (43) | 0.79 | 0.21 | 2.89 | 0.716 |  | 1.00 | 0.27 | 3.73 | 0.995 |
|  | Don't know/refused | 25.82 | (260) | 20.46 | (1280) | 1.06 | 0.88 | 1.28 | 0.545 |  | 1.00 | 0.82 | 1.21 | 0.970 |
| ***Domain: School WASH factors*** | | | | | | | | | | | | | | |
| Location**^i^** | Rural | 42.3 | (445) | 58.77 | (3869) | Reference |  |  | * |  | Reference |  |  | ^ |
|  | Remote | 34.7 | (365) | 32.43 | (2135) | 1.91 | 0.97 | 3.76 | 0.063 |  | 1.79 | 0.92 | 3.48 | 0.085 |
|  | Very remote | 23 | (242) | 8.8 | (579) | 5.51 | 2.01 | 15.05 | <0.01 |  | 4.49 | 1.60 | 12.57 | <0.01 |
| School water source**^j^** | Improved | 34.98 | (368) | 43.86 | (2887) | Reference |  |  | * |  | Reference |  |  | ^ |
|  | Unimproved | 22.43 | (236) | 12.93 | (851) | 2.73 | 1.01 | 7.35 | 0.048 |  | 1.94 | 0.77 | 4.90 | 0.159 |
|  | No water | 25 | (263) | 27.68 | (1822) | 1.26 | 0.56 | 2.80 | 0.579 |  | 0.69 | 0.33 | 1.46 | 0.331 |
|  | Don't know/refused | 17.59 | (185) | 15.54 | (1023) | 1.92 | 0.74 | 5.03 | 0.181 |  | 1.58 | 0.68 | 3.70 | 0.290 |
| School toilets available to students | | 94.01 | (989) | 97.4 | (6412) | 0.30 | 0.05 | 1.88 | 0.201 |  | - | - | - | - |
| School sanitation**^k^** | Improved | 95.15 | (1001) | 95.26 | (6217) | Reference |  |  |  |  | - | - | - | - |
|  | Unimproved | 4.85 | (51) | 4.74 | (312) | 0.52 | 0.09 | 2.84 | 0.450 |  | - | - | - | - |
| School handwashing station**^l^** | No | 9.51 | (99) | 11.8 | (750) | Reference |  |  | * |  | Reference |  |  | ^ |
|  | Yes, but no soap | 70.89 | (738) | 54.01 | (3433) | 2.26 | 0.80 | 6.41 | 0.125 |  | 3.09 | 1.13 | 8.45 | 0.028 |
|  | Yes, soap available | 19.6 | (204) | 34.19 | (2173) | 0.63 | 0.21 | 1.90 | 0.408 |  | 0.98 | 0.33 | 2.91 | 0.964 |
| Latrine/toilet able to be locked | | 11.69 | (123) | 5.35 | (352) | 2.06 | 0.52 | 8.13 | 0.301 |  | - | - | - | - |
| Latrine/toilet observed to be clean | | 42.3 | (445) | 61.73 | (4064) | 0.51 | 0.27 | 1.00 | 0.050 |  | 0.83 | 0.44 | 1.60 | 0.587 |
| Latrine/toilet has water for cleaning self | | 48.86 | (514) | 48.47 | (3191) | 1.16 | 0.59 | 2.27 | 0.675 |  | - | - | - | - |
| Latrine/toilet observed to be dirty | | 45.63 | (480) | 29.47 | (1940) | 1.60 | 0.78 | 3.28 | 0.201 |  | - | - | - | - |
| Latrine/toilet observed to have flies | | 22.15 | (233) | 14.75 | (971) | 1.28 | 0.50 | 3.26 | 0.605 |  | - | - | - | - |
| Number of school toilets | | 5.2 | (3) | 5.2 | (4) | 1.02 | 0.93 | 1.11 | 0.716 |  | - | - | - | - |
| **Notes:** WASH = water, sanitation and hygiene. All hookworm = infection with at least one of *N. americanus, A, duodanale or A. ceylanicum*. aOR = adjusted odds ratio, OR = odds ratio, 95% CI = 95% confidence interval, STH = soil-transmitted helminths. Reference = reference category used for regression for categorical variables. **^†^**Numbers and proportion of qPCR positive and negative participants for each variable, except for continuous variables, reported as mean ± standard deviation. **^a^**Domain multivariable models conducted using covariates with p<0.2 on the Wald test from univariate analysis indicated by *. All domain level multivariate models included the covariates of sex and age. ^Denotes varibales from domain multivariable models with p<0.1 on Wald test and included in backwards stepwise elimination procedure to produce final multivariable models. **^b^**See Supplementary table 1 for list ethnicities grouped in 'Other'. **^c^**Captures students who report ever defecating outside at home or school. **^d,e^**Responses for variables collapsed to align (or approximate where not enough information available) with WHO and United Nations International Children’s Emergency Fund (UNICEF) Joint Monitoring Programme (JMP) for Water Supply and Sanitation definitions. **^d^**Improved = piped water, protected well, rainwater or public tap. Unimproved = unprotected well or surface water. **^e^**Improved = flush toilet or pit latrine with a slab. Unimproved = pit latrine without a slab. (-)Indicates categories not included in regression at that step. #Age and grade level found to be only variables collinear (VIF>5) in initial final multivariable model. Initial models tested with age and grade independently and the model with age had a lower AIC than that with grade. Grade was dropped from the analysis at this stage prior to stepwise regression. | | | | | | | | | | | | | | |

| **Supplementary table 13.** Sensitivity analysis adjusting for clustering at school and hamlet level: Factors associated with moderate-to-heavy intensity *N. americanus* infection, univariate and domain level multivariate analysis | | | | | | | | | | | | | | |
| --- | --- | --- | --- | --- | --- | --- | --- | --- | --- | --- | --- | --- | --- | --- |
|  |  | **Moderate-to-heavy intensity infection, n=251** | | **Light intensity infection, n =801** | | **Univariate analysis** | | | |  | **Domain Multivariate analysis^a^** | | | |
| ***Variables*** |  | **% (n)** | | **% (n)** | | **OR** | **95% CI** | | **p-value** |  | **aOR** | **95% CI** | | **p-value** |
| ***Domain: demographics*** | | | | | | | | | | | | | | |
| Age (years) |  | 8.4 | (1) | 8.2 | (1) | 1.13 | 1.01 | 1.26 | 0.03* |  | 1.21 | 0.98 | 1.48 | 0.07^ |
| Female |  | 46.61 | (117) | 42.57 | (341) | 1.12 | 0.82 | 1.51 | 0.48* |  | 1.11 | 0.81 | 1.51 | 0.53^ |
| Ethnicity**^b^** | Kinh | 3.19 | (8) | 7.99 | (64) | Reference |  |  | * |  | Reference |  |  | ^ |
|  | Dao | 0.4 | (1) | 2.25 | (18) | 0.29 | 0.03 | 2.93 | 0.297 |  | 0.26 | 0.03 | 2.65 | 0.256 |
|  | Gia-rai | 11.16 | (28) | 8.24 | (66) | 3.94 | 1.19 | 13.13 | 0.025 |  | 4.02 | 1.19 | 13.62 | 0.025 |
|  | Hmông | 13.55 | (34) | 11.24 | (90) | 3.80 | 1.25 | 11.57 | 0.019 |  | 3.61 | 1.17 | 11.07 | 0.025 |
|  | Mnông | 12.75 | (32) | 11.36 | (91) | 2.86 | 0.93 | 8.76 | 0.067 |  | 2.78 | 0.90 | 8.58 | 0.075 |
|  | Nùng | 2.79 | (7) | 4.24 | (34) | 1.66 | 0.49 | 5.65 | 0.416 |  | 1.70 | 0.50 | 5.83 | 0.399 |
|  | Tày | 1.59 | (4) | 2.25 | (18) | 1.98 | 0.47 | 8.35 | 0.353 |  | 2.00 | 0.47 | 8.50 | 0.347 |
|  | Xơ-đăng | 6.77 | (17) | 4.37 | (35) | 4.27 | 1.12 | 16.35 | 0.034 |  | 4.03 | 1.04 | 15.53 | 0.043 |
|  | Êđê | 45.42 | (114) | 45.94 | (368) | 2.92 | 1.21 | 7.04 | 0.017 |  | 2.86 | 1.18 | 6.94 | 0.020 |
|  | Other | 2.39 | (6) | 2.12 | (17) | 2.78 | 0.73 | 10.63 | 0.134 |  | 2.45 | 0.63 | 9.53 | 0.197 |
| School graded | Grade 1 | 19.52 | (49) | 23.6 | (189) | Reference |  |  | * |  | Reference |  |  |  |
|  | Grade 2 | 27.09 | (68) | 23.85 | (191) | 1.47 | 0.94 | 2.29 | 0.088 |  | 1.23 | 0.73 | 2.06 | 0.439 |
|  | Grade 3 | 24.3 | (61) | 26.97 | (216) | 1.11 | 0.71 | 1.73 | 0.654 |  | 0.74 | 0.39 | 1.42 | 0.371 |
|  | Grade 4 | 29.08 | (73) | 25.59 | (205) | 1.49 | 0.96 | 2.33 | 0.077 |  | 0.85 | 0.38 | 1.89 | 0.689 |
| ***Domain: co-infection with other STH species*** | | | | | | | | | | | | | | |
| *A. lumbricoides* | | 0.4 | (1) | 0.5 | (4) | 1.33 | 0.13 | 13.80 | 0.812 |  | - | - | - | - |
| *T. trichiura* |  | 1.59 | (4) | 0.75 | (6) | 1.90 | 0.48 | 7.46 | 0.359 |  | - | - | - | - |
| *S. stercoralis* |  | 4.38 | (11) | 1.5 | (12) | 2.36 | 0.97 | 5.73 | 0.058* |  | 2.255384^ | 0.93 | 5.48 | 0.073 |
| *A. ceylanicum* | | 2.39 | (6) | 2.12 | (17) | 1.45 | 0.53 | 3.97 | 0.472 |  | - | - | - | - |
| ***Domain: Health, personal hygiene and defecation behaviour*** | | | | | | | | | | | | | | |
| Had deworming within the last year | No | 50.62 | (123) | 51.97 | (396) | Reference |  |  |  |  | - | - | - | - |
|  | Yes | 45.68 | (111) | 45.14 | (344) | 0.98 | 0.69 | 1.37 | 0.887 |  | - | - | - | - |
|  | Don't know/refused | 3.7 | (9) | 2.89 | (22) | 1.32 | 0.54 | 3.18 | 0.542 |  | - | - | - | - |
| Usual place of defecation at home**^c^** | Household toilet/latrine | 53.31 | (129) | 52.96 | (403) | Reference |  |  |  |  | - | - | - | - |
|  | Shared toilet/latrine | 2.48 | (6) | 1.58 | (12) | 1.24 | 0.43 | 3.64 | 0.691 |  | - | - | - | - |
|  | On the ground outside/bushes or grass | 43.8 | (106) | 44.42 | (338) | 0.92 | 0.66 | 1.30 | 0.646 |  | - | - | - | - |
|  | Other/Don't know/refused | 0.41 | (1) | 1.05 | (8) | 0.37 | 0.04 | 3.20 | 0.363 |  | - | - | - | - |
| Ever defecates outside at home**^d^** | | 68.88 | (166) | 67.46 | (512) | 0.98 | 0.69 | 1.39 | 0.921 |  | - | - | - | - |
| Usual place of defecation at school | Toilet/latrine | 79.01 | (192) | 84.89 | (646) | Reference |  |  | * |  | Reference |  |  | ^ |
|  | On the ground outside/bushes or grass | 17.7 | (43) | 11.56 | (88) | 1.63 | 0.97 | 2.73 | 0.067 |  | 1.74 | 1.02 | 2.97 | 0.041 |
|  | Doesn't defecate at school | 1.65 | (4) | 1.84 | (14) | 1.05 | 0.30 | 3.61 | 0.944 |  | 1.04 | 0.30 | 3.57 | 0.954 |
|  | Don't know/refused | 1.65 | (4) | 1.71 | (13) | 0.94 | 0.27 | 3.22 | 0.918 |  | 0.89 | 0.26 | 3.09 | 0.855 |
| Ever defecates outside at school**^d^** | | 28.40 | (69) | 24.41 | (185) | 1.09 | 0.73 | 1.62 | 0.664 |  | - | - | - | - |
| Washes hands after defecating | Always | 41.56 | (101) | 47.97 | (366) | Reference |  |  | * |  | Reference |  |  |  |
|  | Sometimes | 49.38 | (120) | 41.15 | (314) | 1.27 | 0.91 | 1.79 | 0.160 |  | 1.36 | 0.96 | 1.93 | 0.083 |
|  | Never | 9.05 | (22) | 10.75 | (82) | 0.92 | 0.52 | 1.64 | 0.786 |  | 0.95 | 0.53 | 1.71 | 0.868 |
|  | Don’t know/refused | 0 | (0) | 0.13 | (1) | - | - | - | - |  | - | - | - | - |
| Washes hands before eating | Always | 36.36 | (88) | 39.97 | (305) | Reference |  |  |  |  | - | - | - | - |
|  | Sometimes | 55.79 | (135) | 51.51 | (393) | 1.07 | 0.76 | 1.52 | 0.692 |  | - | - | - | - |
|  | Never | 7.44 | (18) | 8.39 | (64) | 0.95 | 0.51 | 1.76 | 0.870 |  | - | - | - | - |
|  | Don’t know/refused | 0.41 | (1) | 0.13 | (1) | 2.76 | 0.15 | 51.37 | 0.496 |  | - | - | - | - |
| Wears shoes outside | Always | 65.84 | (160) | 65.05 | (495) | Reference |  |  | * |  | Reference |  |  |  |
|  | Sometimes | 30.86 | (75) | 32.85 | (250) | 0.94 | 0.66 | 1.34 | 0.726 |  | 0.91 | 0.63 | 1.32 | 0.629 |
|  | Never | 3.29 | (8) | 1.97 | (15) | 1.97 | 0.75 | 5.15 | 0.166 |  | 1.87 | 0.71 | 4.93 | 0.208 |
|  | Don’t know/refused | 0 | (0) | 0.13 | (1) | - | - | - | - |  | - | - | - | - |
| Wears shoes defecating | Always | 73.03 | (176) | 73.26 | (559) | Reference |  |  |  |  | - | - | - | - |
|  | Sometimes | 15.77 | (38) | 16.78 | (128) | 0.94 | 0.60 | 1.45 | 0.769 |  | - | - | - | - |
|  | Never | 10.37 | (25) | 9.31 | (71) | 1.21 | 0.71 | 2.06 | 0.472 |  | - | - | - | - |
|  | Don’t know/refused | 0.83 | (2) | 0.66 | (5) | 1.20 | 0.21 | 6.95 | 0.837 |  | - | - | - | - |
| ***Domain: Household WASH factors*** | | | | | | | | | | | | | | |
| Household water supply**^e^** | Improved | 76.79 | (182) | 74.55 | (574) | Reference |  |  | * |  | Reference |  |  |  |
|  | Unimproved | 22.78 | (54) | 23.9 | (184) | 0.88 | 0.59 | 1.31 | 0.525 |  | 0.91 | 0.61 | 1.36 | 0.649 |
|  | Don't know/refused | 0.42 | (1) | 1.56 | (12) | 0.25 | 0.03 | 2.02 | 0.194 |  | - | - | - | - |
| Household toilet/latrine**^f^** | No | 48.76 | (118) | 48.75 | (371) | Reference |  |  |  |  | - | - | - | - |
|  | Yes, but does not flush | 18.6 | (45) | 17.35 | (132) | 1.20 | 0.77 | 1.85 | 0.421 |  | - | - | - | - |
|  | Yes, flushes with water | 32.64 | (79) | 33.9 | (258) | 1.02 | 0.70 | 1.48 | 0.921 |  | - | - | - | - |
| Handwashing station**^g^** | No | 23.33 | (56) | 16.03 | (121) | Reference |  |  | * |  | Reference |  |  | ^ |
|  | Yes, but no soap available | 32.5 | (78) | 36.56 | (276) | 0.59 | 0.36 | 0.96 | 0.035 |  | 0.57 | 0.34 | 0.93 | 0.025 |
|  | Yes, soap available | 44.17 | (106) | 47.42 | (358) | 0.63 | 0.39 | 1.02 | 0.059 |  | 0.67 | 0.41 | 1.08 | 0.101 |
| ***Domain: Household socioeconomic factors*** | | | | | | | | | | | | | | |
| Mother's highest level of education | Never attended school | 21.94 | (52) | 18.7 | (144) | Reference |  |  | * |  | Reference |  |  |  |
|  | Completed or attended primary school | 43.04 | (102) | 43.38 | (334) | 0.87 | 0.57 | 1.33 | 0.512 |  | 0.88 | 0.58 | 1.35 | 0.567 |
|  | Completed or attended secondary school | 29.96 | (71) | 32.21 | (248) | 0.86 | 0.54 | 1.36 | 0.521 |  | 0.92 | 0.58 | 1.47 | 0.737 |
|  | Completed or attended tertiary education | 1.27 | (3) | 2.99 | (23) | 0.41 | 0.11 | 1.48 | 0.172 |  | 0.43 | 0.12 | 1.56 | 0.197 |
|  | Don't know/refused | 3.8 | (9) | 2.73 | (21) | 1.22 | 0.50 | 3.02 | 0.661 |  | 1.23 | 0.50 | 3.05 | 0.651 |
| Occupation main household income earner**^h^** | Farmer/animal keeper | 97.05 | (230) | 97.53 | (750) | Reference |  |  |  |  | - | - | - | - |
|  | Fisherman/market seller | 0.84 | (2) | 0.52 | (4) | 1.62 | 0.27 | 9.68 | 0.597 |  | - | - | - | - |
|  | Administration/healthcare worker/Other | 0.42 | (1) | 0.52 | (4) | 0.68 | 0.06 | 7.62 | 0.756 |  | - | - | - | - |
|  | Unemployed | 0.42 | (1) | 0.65 | (5) | 0.74 | 0.08 | 7.30 | 0.800 |  | - | - | - | - |
|  | Don't know/refused | 1.27 | (3) | 0.78 | (6) | 1.24 | 0.28 | 5.47 | 0.773 |  | - | - | - | - |
| Household income | Less than VND 20,000,000 | 56.12 | (133) | 55.45 | (427) | Reference |  |  |  |  | - | - | - | - |
|  | VND 20,000,000 – 50,000,000 | 14.77 | (35) | 15.06 | (116) | 0.98 | 0.62 | 1.55 | 0.935 |  | - | - | - | - |
|  | VND 50,000,000 – 200,000,000 | 2.95 | (7) | 3.38 | (26) | 0.73 | 0.29 | 1.84 | 0.505 |  | - | - | - | - |
|  | More than VND 200,000,000 | 0.42 | (1) | 0.26 | (2) | 1.24 | 0.10 | 15.15 | 0.868 |  | - | - | - | - |
|  | Don't know/refused | 25.74 | (61) | 25.84 | (199) | 1.07 | 0.74 | 1.56 | 0.719 |  | - | - | - | - |
| ***Domain: School location and WASH factors*** | | | | | | | | | | | | | | |
| Location**^i^** | Rural | 39.04 | (98) | 43.32 | (347) | Reference |  |  |  |  | - | - | - | - |
|  | Remote | 39.04 | (98) | 33.33 | (267) | 1.22 | 0.72 | 2.07 | 0.471 |  | - | - | - | - |
|  | Very remote | 21.91 | (55) | 23.35 | (187) | 0.92 | 0.46 | 1.85 | 0.823 |  | - | - | - | - |
| School water source**^j^** | Improved | 43.03 | (108) | 32.46 | (260) | Reference |  |  | * |  | Reference |  |  |  |
|  | Unimproved | 21.12 | (53) | 22.85 | (183) | 0.69 | 0.36 | 1.32 | 0.259 |  | 0.69 | 0.36 | 1.32 | 0.259 |
|  | No water | 21.91 | (55) | 25.97 | (208) | 0.67 | 0.37 | 1.20 | 0.180 |  | 0.67 | 0.37 | 1.20 | 0.180 |
|  | Don't know/refused | 13.94 | (35) | 18.73 | (150) | 0.57 | 0.29 | 1.11 | 0.097 |  | 0.57 | 0.29 | 1.11 | 0.097 |
| School toilets available to students | | 92.03 | (231) | 94.63 | (758) | 0.49 | 0.17 | 1.42 | 0.189* |  | 1.64 | 0.47 | 5.72 | 0.439 |
| School sanitation**^k^** | Improved | 94.02 | (236) | 95.51 | (765) | Reference |  |  |  |  | - | - | - | - |
|  | Unimproved | 5.98 | (15) | 4.49 | (36) | 1.84 | 0.51 | 6.58 | 0.350 |  | - | - | - | - |
| School handwahsing station**^l^** | No | 15.2 | (38) | 7.71 | (61) | Reference |  |  | * |  | Reference |  |  | ^ |
|  | Yes, but no soap | 66 | (165) | 72.44 | (573) | 0.36 | 0.17 | 0.75 | <0.01 |  | 0.29 | 0.11 | 0.76 | 0.012 |
|  | Yes, soap available | 18.8 | (47) | 19.85 | (157) | 0.33 | 0.14 | 0.78 | 0.012 |  | 0.28 | 0.10 | 0.77 | 0.014 |
| Latrine/toilet able to be locked | | 10.76 | (27) | 11.99 | (96) | 0.93 | 0.38 | 2.25 | 0.870 |  | - | - | - | - |
| Latrine/toilet observed to be clean | | 45.02 | (113) | 41.45 | (332) | 1.20 | 0.74 | 1.94 | 0.468 |  | 0.83 | 0.44 | 1.60 | 0.587 |
| Latrine/toilet has water for cleaning self | | 44.22 | (111) | 50.31 | (403) | 0.75 | 0.46 | 1.22 | 0.251 |  | - | - | - | - |
| Latrine/toilet observed to be dirty | | 43.82 | (110) | 46.19 | (370) | 0.90 | 0.54 | 1.49 | 0.671 |  | - | - | - | - |
| Latrine/toilet observed to have flies | | 19.92 | (50) | 22.85 | (183) | 0.91 | 0.48 | 1.75 | 0.788 |  | - | - | - | - |
| Number of school toilets | | 5.3 | (4) | 5.2 | (3) | 1.00 | 0.94 | 1.07 | 0.960 |  | - | - | - | - |
| **Notes:** WASH = water, sanitation and hygiene. All hookworm = infection with at least one of *N. americanus, A, duodanale or A. ceylanicum*. aOR = adjusted odds ratio, OR = odds ratio, 95% CI = 95% confidence interval, STH = soil-transmitted helminths. Reference = reference category used for regression for categorical variables. **^†^**Numbers and proportion of qPCR positive and negative participants for each variable, except for continuous variables, reported as mean ± standard deviation. **^a^**Domain multivariable models conducted using covariates with p<0.2 on the Wald test from univariate analysis indicated by *. All domain level multivariate models included the covariates of sex and age. ^Denotes varibales from domain multivariable models with p<0.1 on Wald test and included in backwards stepwise elimination procedure to produce final multivariable models. **^b^**See Supplementary table 1 for list ethnicities grouped in 'Other'. **^c^**Captures students who report ever defecating outside at home or school. **^d,e^**Responses for variables collapsed to align (or approximate where not enough information available) with WHO and United Nations International Children’s Emergency Fund (UNICEF) Joint Monitoring Programme (JMP) for Water Supply and Sanitation definitions. **^d^**Improved = piped water, protected well, rainwater or public tap. Unimproved = unprotected well or surface water. **^e^**Improved = flush toilet or pit latrine with a slab. Unimproved = pit latrine without a slab. (-)Indicates categories not included in regression at that step. | | | | | | | | | | | | | | |
