# Supplementary material for: Epidemiology of soil-transmitted helminths using quantitative PCR and risk factors for hookworm and Necator americanus infection in school children in Dak Lak province, Vietnam
Source: Parasit Vectors. 2023 Jun 27;16:213. doi: 10.1186/s13071-023-05809-x (PMC10304358; doi:10.1186/s13071-023-05809-x)
Supplement: Supplementary file 2 — Additional file 2. STROBE checklist—reporting checklist for cross-sectional studies. [file 13071_2023_5809_MOESM2_ESM.docx]

STROBE Statement—Checklist of items that should be included in reports of ***cross-sectional studies***

|  | Item No | Recommendation |  |
| --- | --- | --- | --- |
| **Title and abstract** | 1 | (*a*) Indicate the study’s design with a commonly used term in the title or the abstract | 🗸  Ttitle and abstract (page 1 and 2) |
|  |  | (*b*) Provide in the abstract an informative and balanced summary of what was done and what was found | 🗸  Abstract and author summary (page 2 and 3) |
| Introduction | | | |
| Background/rationale | 2 | Explain the scientific background and rationale for the investigation being reported | 🗸  Introduction (page 4 and 5) |
| Objectives | 3 | State specific objectives, including any prespecified hypotheses | 🗸  Introduction (page 5) |
| Methods | | | |
| Study design | 4 | Present key elements of study design early in the paper | 🗸  Methods (page 5) - Study setting, design and participants |
| Setting | 5 | Describe the setting, locations, and relevant dates, including periods of recruitment, exposure, follow-up, and data collection | 🗸  Methods (page 5, 6) – Study setting, design and participants  Methods (page 6, 7) – Demographic, socioeconomic and wash data collection, and Specimen collection and analysis |
| Participants | 6 | (*a*) Give the eligibility criteria, and the sources and methods of selection of participants | 🗸  Methods (page 5, 6) – Study setting, design and participants  Methods (page 6, 7) – Demographic, socioeconomic and wash data collection, and Specimen collection and analysis |
| Variables | 7 | Clearly define all outcomes, exposures, predictors, potential confounders, and effect modifiers. Give diagnostic criteria, if applicable | 🗸  Methods (page 6,7,8) – Specimen collection and analysis and Statistical analysis |
| Data sources/ measurement | 8* | For each variable of interest, give sources of data and details of methods of assessment (measurement). Describe comparability of assessment methods if there is more than one group | 🗸  Methods (pages 5 to 9)  Study setting, design and participants, Demographic, socioeconomic and wash data collection, and Specimen collection and analysis, Georeferencing of schools, Statistical analysis |
| Bias | 9 | Describe any efforts to address potential sources of bias | 🗸  Methods (page 7, 8) – statistical analysis  Discussion (page 22) |
| Study size | 10 | Explain how the study size was arrived at | 🗸  Methods (page 5) - Study setting, design and participants |
| Quantitative variables | 11 | Explain how quantitative variables were handled in the analyses. If applicable, describe which groupings were chosen and why | 🗸  Methods (page 7, 8) – statistical analysis |
| Statistical methods | 12 | (*a*) Describe all statistical methods, including those used to control for confounding | 🗸  Methods (page 7, 8) – statistical analysis |
|  |  | (*b*) Describe any methods used to examine subgroups and interactions | 🗸  Methods (page 7, 8) – statistical analysis |
|  |  | (*c*) Explain how missing data were addressed | 🗸  Methods (page 7, 8) – statistical analysis |
|  |  | (*d*) If applicable, describe analytical methods taking account of sampling strategy | 🗸  Methods (page 7, 8) – statistical analysis |
|  |  | (*e*) Describe any sensitivity analyses | 🗸  Methods (page 8, 9) – statistical analysis |
| Results | | | |
| Participants | 13* | (a) Report numbers of individuals at each stage of study—eg numbers potentially eligible, examined for eligibility, confirmed eligible, included in the study, completing follow-up, and analysed | 🗸  Results (page 9, 10) – Participant characteristics, table 1 and S1 supplementary figure 1 and supplementary tables 1, 2 and 3 |
|  |  | (b) Give reasons for non-participation at each stage | 🗸  Results (page 9, 10) – Participant characteristics and S1 supplementary table 3 |
|  |  | (c) Consider use of a flow diagram | 🗸  S1 – supplementary figure 1 |
| Descriptive data | 14* | (a) Give characteristics of study participants (eg demographic, clinical, social) and information on exposures and potential confounders | 🗸  Results (page 9, 10, 11) Table 1 S1supplementary tables 1, 2 and 3 |
|  |  | (b) Indicate number of participants with missing data for each variable of interest | 🗸  Results (page 11 to 14), Prevalence and STH intensity, table 2 and 3 and fig 1.  S1supplementary tables 3 to 13 |
| Outcome data | 15* | Report numbers of outcome events or summary measures | 🗸  S1supplementary tables 4 to 13 |
| Main results | 16 | (*a*) Give unadjusted estimates and, if applicable, confounder-adjusted estimates and their precision (eg, 95% confidence interval). Make clear which confounders were adjusted for and why they were included | 🗸  Results (page 11 to 19), Prevalence and STH intensity, Risk factors for hookworm and N. americanus infection and Risk factors for moderate-to-heavy N. americanus infection Tables 4, 5 and S1 Supplementary tables 3-13 |
|  |  | (*b*) Report category boundaries when continuous variables were categorized | 🗸  S1 Supplementary tables 3-13 |
|  |  | (*c*) If relevant, consider translating estimates of relative risk into absolute risk for a meaningful time period | NA – cross-sectional study |
| Other analyses | 17 | Report other analyses done—eg analyses of subgroups and interactions, and sensitivity analyses | 🗸  Results (page 19) – Sensitivity analysis and S1 supplementary tables 9-13 |
| Discussion | | | |
| Key results | 18 | Summarise key results with reference to study objectives | 🗸  Discussion (pages 19 to 21) |
| Limitations | 19 | Discuss limitations of the study, taking into account sources of potential bias or imprecision. Discuss both direction and magnitude of any potential bias | 🗸  Discussion (page 22) |
| Interpretation | 20 | Give a cautious overall interpretation of results considering objectives, limitations, multiplicity of analyses, results from similar studies, and other relevant evidence | 🗸  Discussion (page 22) |
| Generalisability | 21 | Discuss the generalisability (external validity) of the study results | 🗸  Discussion (page 22) |
| Other information | | | |
| Funding | 22 | Give the source of funding and the role of the funders for the present study and, if applicable, for the original study on which the present article is based | 🗸  Methods (page 9) – Ethics approval and consent  Funding statement/disclosure |

*Give information separately for exposed and unexposed groups.

**Note:** An Explanation and Elaboration article discusses each checklist item and gives methodological background and published examples of transparent reporting. The STROBE checklist is best used in conjunction with this article (freely available on the Web sites of PLoS Medicine at http://www.plosmedicine.org/, Annals of Internal Medicine at http://www.annals.org/, and Epidemiology at http://www.epidem.com/). Information on the STROBE Initiative is available at www.strobe-statement.org.
